# Supplementary material for: In-situ cryo-immune engineering of tumor microenvironment with cold-responsive nanotechnology for cancer immunotherapy
Source: Nat Commun. 2023 Jan 24;14:392. doi: 10.1038/s41467-023-36045-7 (PMC9873931; doi:10.1038/s41467-023-36045-7)
Supplement: Supplementary file 1 — Supplementary Information [file 41467_2023_36045_MOESM1_ESM.pdf]

Supplementary Information

(Supplementary Figures 1-27)

In-situ cryo-immune engineering of tumor microenvironment with cold-responsive nanotechnology for cancer immunotherapy

Wenquan Ou[1], Samantha Stewart[1], Alisa White[1], Elyahb A. Kwizera[1], Jiangsheng Xu[1], Yuanzhang Fang[2], James G. Shamul[1], Changqing Xie[3], Suliat Nurudeen[4], Nikki P. Tirada[4], Xiongbin Lu[2], Katherine H.R. Tkaczuk[4], Xiaoming He[1,4]*

[1]Fischell Department of Bioengineering, University of Maryland, College Park, MD 20742, USA.

[2]Department of Medical and Molecular Genetics and Melvin and Bren Simon Cancer Center, Indiana University School of Medicine, Indianapolis, Indiana 46202, USA

[3]Thoracic and Gastrointestinal Malignancies Branch, Center for Cancer Research, National Cancer Institute, National Institutes of Health, Bethesda, MD 20892, USA.

[4]Marlene and Stewart Greenebaum Comprehensive Cancer Center, University of Maryland, Baltimore, MD 21201, USA.

*Correspondence should be addressed to: Xiaoming He: shawnhe@umd.edu

Supplementary Figures

a

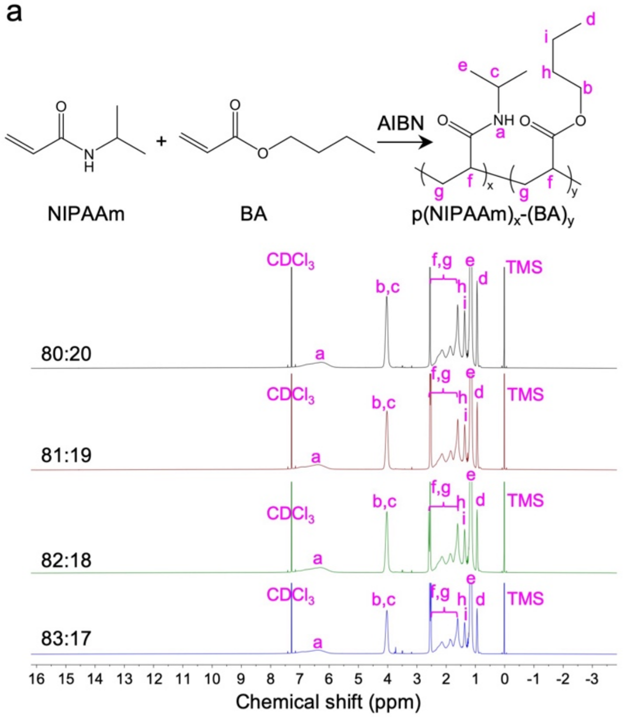

b

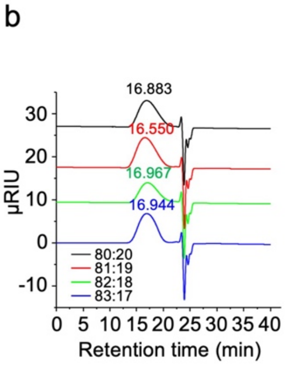

d

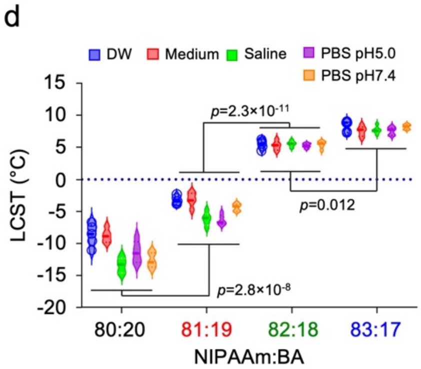

c

NIPAAm:BA	80:20	81:19	82:18	83:17
M_n (Daltons)	66,556 ± 1872	82,967 ± 1251	82,718 ± 2004	78,192 ± 1017
PDI	1.7 ± 0.3	1.7 ± 0.2	1.6 ± 0.2	1.6 ± 0.2
x	473 ± 13	598 ± 8	603 ± 17	577 ± 7
y	101 ± 4	119 ± 3	113 ± 3	100 ± 2
x/y	4.7 ± 0.1	5.0 ± 0.1	5.3 ± 0.1	5.8 ± 0.1

Supplementary Fig. 1 | Synthesis and characterization of cold-responsive pNIPAAm-BA polymers. **a**, Synthetic route and proton-nuclear magnetic resonance (^{1}H-NMR) spectra of pNIPAAm-BA polymers with different NIPAAm to BA ratios, showing successful synthesis of pNIPAAm-BA polymers with typical bonds labeled as a-i. NIPAAm: N-isopropylacrylamide, BA: butyl acrylate, AIBN: 2,20-Azobis(2-methylpropionitrile), $CDCl_3$: deuterated chloroform, and TMS: tetramethylsilane (as the internal reference). **b**, Gel permeation chromatography (GPC) spectra of the synthesized pNIPAAm-BA polymers with different NIPAAm to BA ratios. The data of retention time (t) labeled on the figure was used to calculate the number-averaged molecular weight (M_n) of polymer based on a calibration curve for which the method is detailed in the Methods section. **c**, Molecular weight (M_n), polydispersity index (PDI), and number of each monomer (x for NIPAAm and y for BA) in the different pNIPAAm-BA polymers calculated from ^{1}H-NMR and GPC spectra (n=3 independent experiments). **d**, Higher content (judged by the ratio of x to y) of NIPAAm in the resultant pNIPAAm-BA polymers leads to higher LCST regardless of the solvent/solution where they are dissolved, including deionized water (DW), cell culture medium (medium), saline, and phosphate buffer saline (PBS) at both pH7.4 and 5.0 (n=3 independent experiments). Two-way analysis of variance (ANOVA) with Sidak's post-test and correction for multiple comparisons was used for statistical analyses. Data are presented as mean ± SD (**c**-**d**). Source data are provided as a Source Data file.

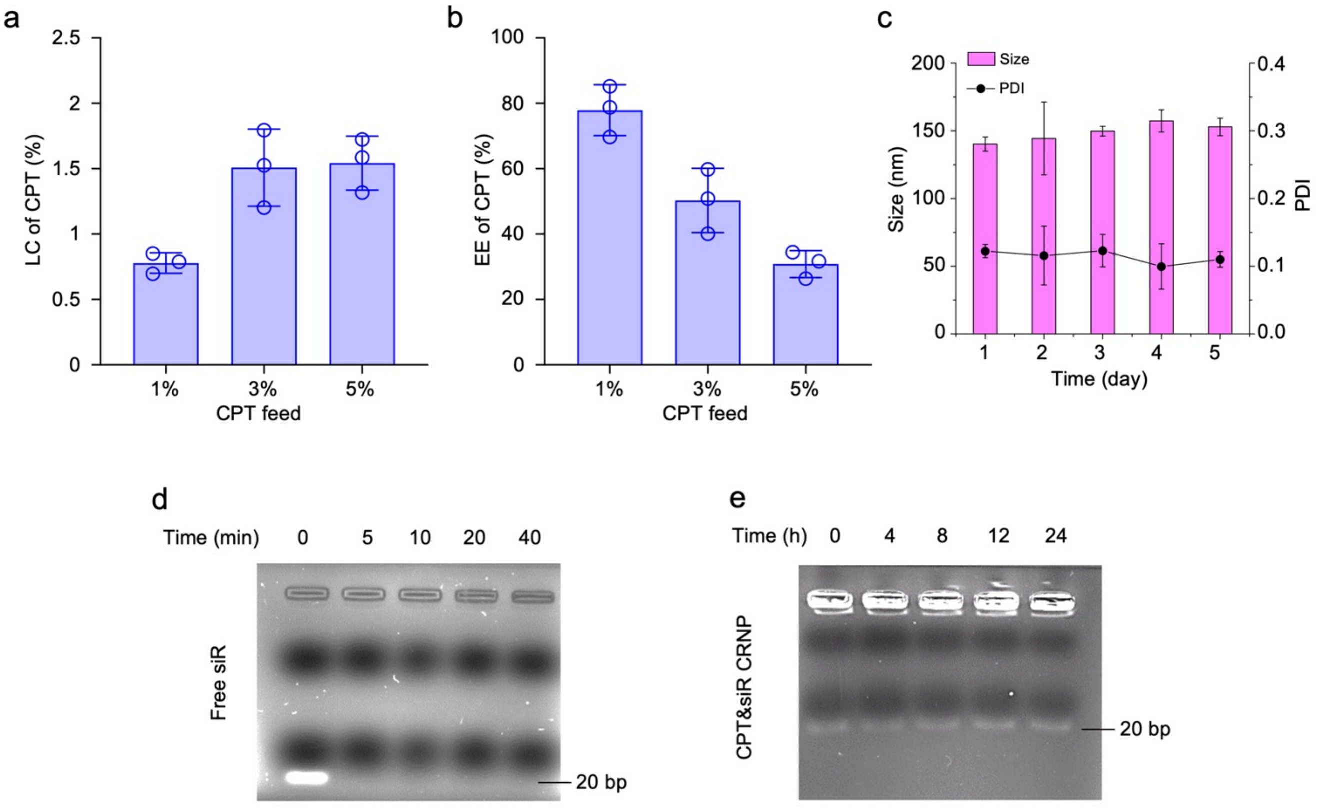

Supplementary Fig. 2 | CPT&siR CRNPs with optimized CPT loading show good stability and prevent siR from fast degradation. a-b, The loading capacity (LC, **a**) and encapsulation efficiency (EE, **b**) of CPT in CRNPs after feeding CPT at 1, 3, and 5%wt of the polymers during the preparation (n=3 independent experiments). **c**, CPT&siR CRNPs display no significant changes in their size and size distribution (indicated by PDI) after incubating with PBS for 5 days (n=3 independent experiments). **d**, Free siR shows fast degradation (within 5 min of incubation with PBS at 22 °C) as determined by the agarose gel electrophoresis. The black bands are due to the background of the 6X loading buffer. **e**, CPT&siR CRNPs prevent siR from degradation after at least 24 h of incubation with PBS at 22 °C, indicated by the evident siR band (white) in agarose gel well. The experiments for **d**-**e** were repeated three times independently with similar results. Data are presented as mean ± SD (**a**-**c**). Source data are provided as a Source Data file.

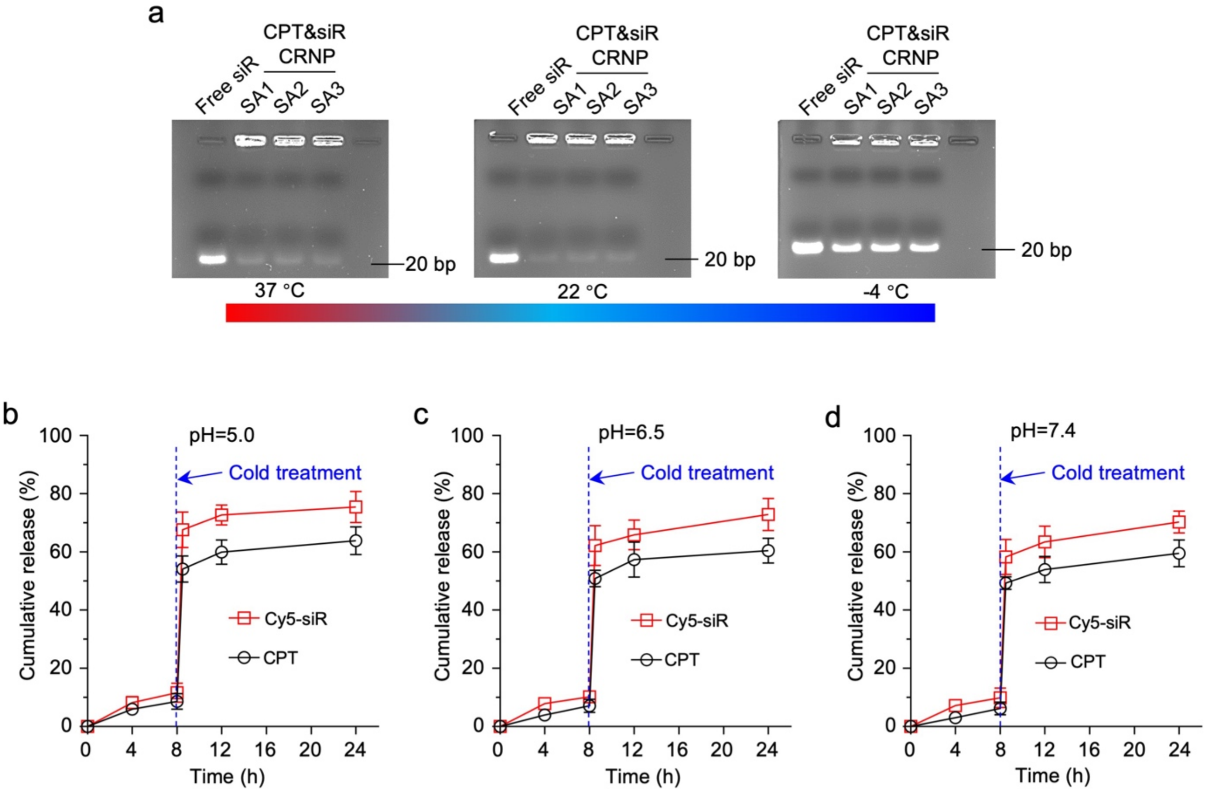

Supplementary Fig. 3 | CPT&siR CRNPs release payload in a cold-triggered manner. a, CPT&siR CRNPs release more siR at -4 °C, but not at 22 °C and 37°C, as shown by the agarose gel electrophoresis. SA: sample. The experiments were repeated three times independently with similar results. **b-d**, CPT&siR CRNPs show cold-triggered release of CPT and siR at pH 5.0 (acetate buffer), pH 6.5 (phosphate buffer), and pH 7.4 (phosphate buffer) (n=3 independent experiments). Cold treatment was performed by incubating the samples at -4 °C for 10 min. Data are presented as mean ± SD (**b-d**). Source data are provided as a Source Data file.

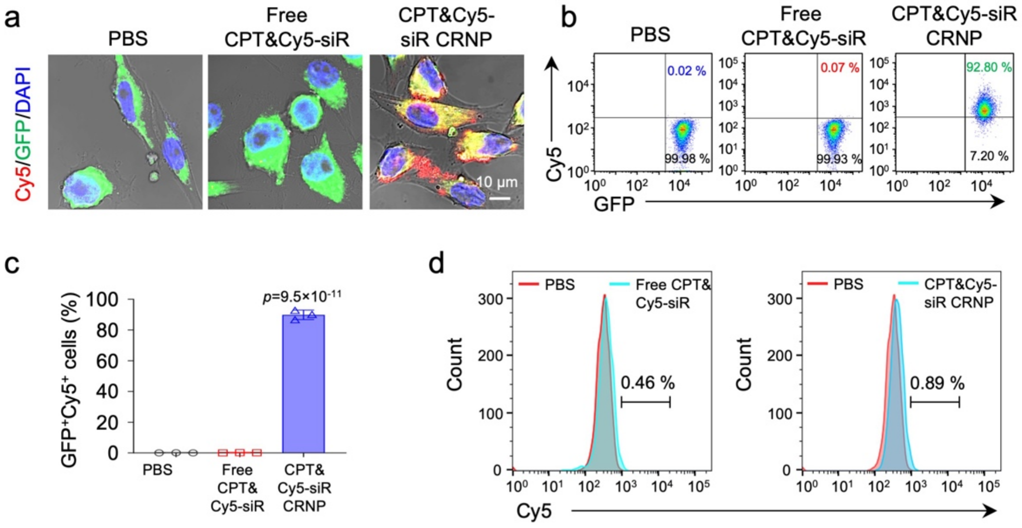

Supplementary Fig. 4 | CPT&Cy5-siR CRNPs enhance cellular uptake of siR in EO771 cancer cells with negligible uptake in lymphocytes. **a**, Cellular uptake of free CPT&Cy5-siR and CPT&Cy5-siR CRNPs in GFP^+ EO771 cells after incubation for 8 h. Green: green fluorescent protein (GFP), blue: 4′,6-diamidino-2-phenylindole (DAPI), and red: cyanine 5 (Cy5). **b-c**, Further determination of cellular uptake in GFP^+ EO771 cells by flow cytometry (**b**) and the corresponding quantitative analysis (**c**) after incubating the cells with PBS, free CPT&Cy5-siR, and CPT&Cy5-siR CRNPs for 8 h (n=3 independent experiments). **d**, Flow cytometry analysis of cellular uptake of free CPT&Cy5-siR and CPT&Cy5-siR CRNPs in normal spleen lymphocytes after incubation for 8 h. The experiments were repeated three times independently with similar results. One-way ANOVA with Tukey's multiple comparisons and correction was used for statistical analyses. Data are presented as mean ± SD (**c**). Source data are provided as a Source Data file.

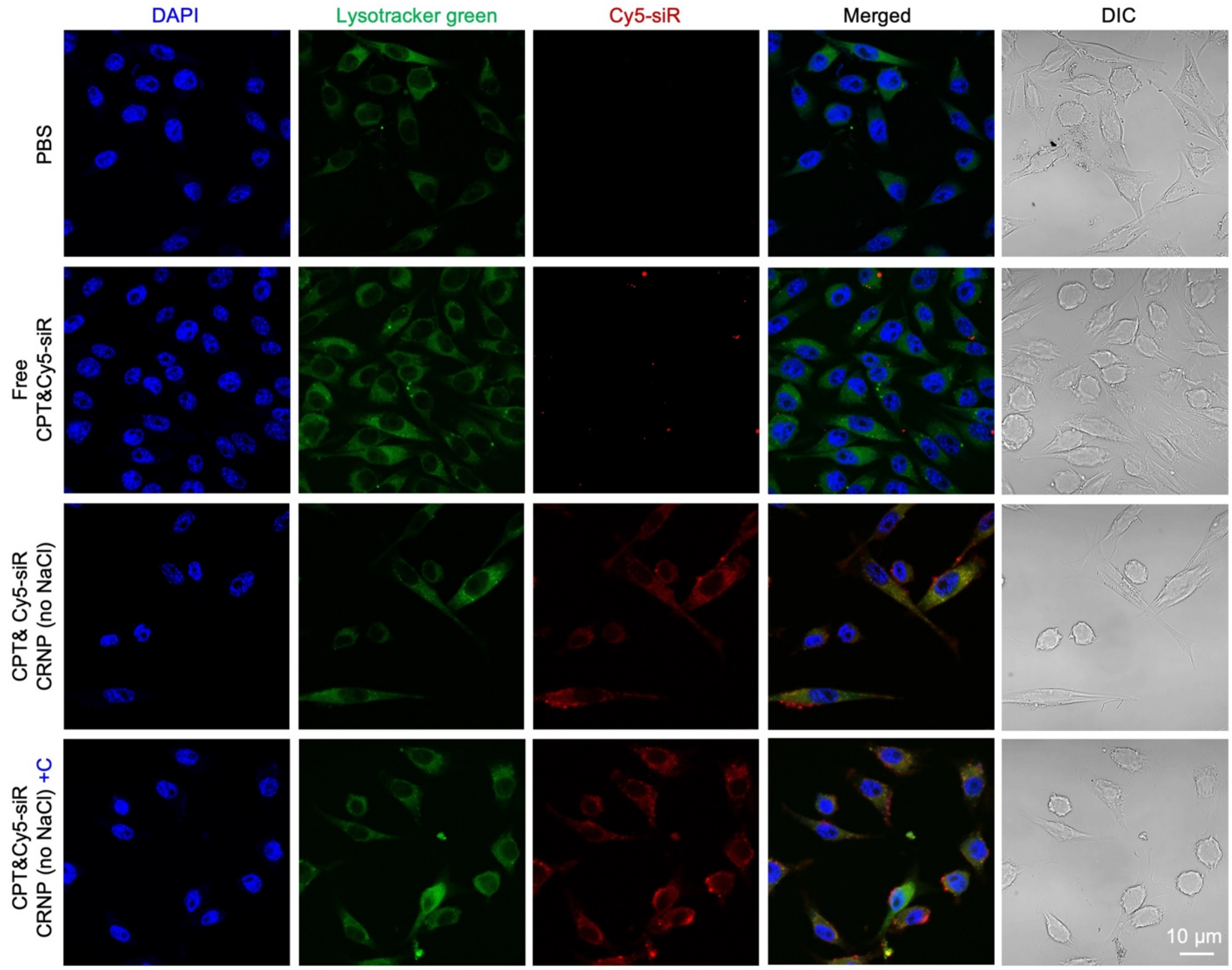

Supplementary Fig. 5 | CPT&Cy5-siR CRNPs with no NaCl induce minimal endo/lysosomal escape. Representative confocal images showing minimal endo/lysosomal escape of Cy-5-siR in EO771 cells treated with free CPT&Cy5-siR, CPT&Cy5-siR CRNPs (no NaCl), CPT&Cy5-siR CRNPs (no NaCl) +C. "+C" indicates the groups with cold treatment at -4 °C for 10 min. NaCl: sodium chloride. The experiments were repeated three times independently with similar results.

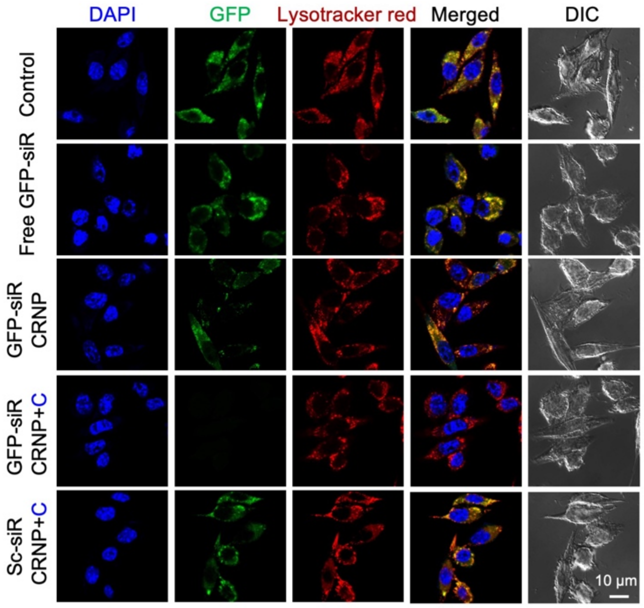

Supplementary Fig. 6 | The treatment of CRNPs+C shows strong GFP silencing effect in GFP⁺ EO771 cells. Representative confocal images of GFP⁺ EO771 cells treated with free GFP-siR, GFP-siR CRNPs, GFP-siR CRNPs+C and Sc-siR CRNPs+C. "+C" indicates the groups with cold treatment at -4 °C for 10 min. Sc-siR: scrambled siR (as negative control). The experiments were repeated three times independently with similar results.

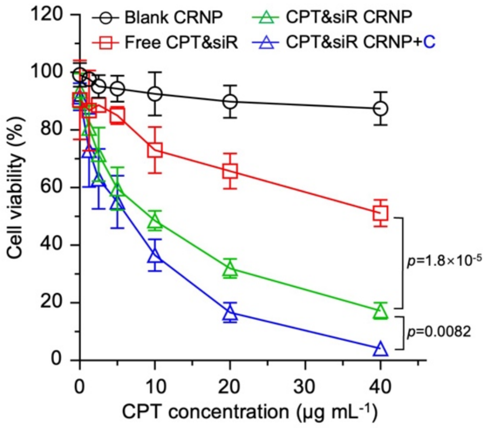

Supplementary Fig. 7 | Cold treatment enhances the in vitro cancer cell killing effect of CPT&siR CRNPs. EO771 cells were treated with blank CRNPs, free CPT&siR, CPT&siR CRNPs, and CPT&siR CRNPs+C. “+C” indicates the groups with cold treatment at -4 °C for 10 min (n=6 independent experiments). Two-way ANOVA with Sidak’s post-test and correction for multiple comparisons was used for statistical analyses. Data are presented as mean ± SD. Source data are provided as a Source Data file.

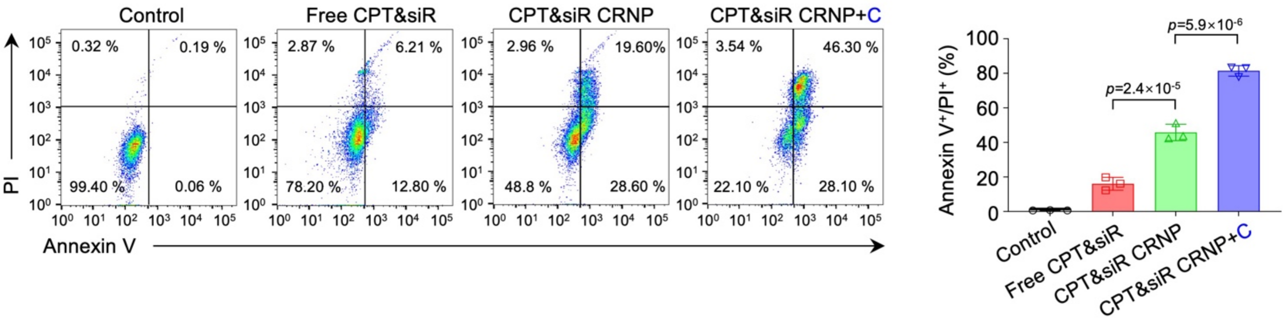

Supplementary Fig. 8 | CPT&siR CRNPs+C induce more apoptosis in cancer cells. EO771 cells treated with PBS control, free CPT&siR, CPT&siR CRNPs, and CPT&siR CRNPs+C were stained with annexin V (for early and late apoptosis) and propidium iodide (PI, for apoptosis/necrosis) (n=3 independent experiments). “+C” indicates the groups with cold treatment at -4 °C for 10 min. One-way ANOVA with Tukey's multiple comparisons and correction was used for statistical analyses. Data are presented as mean ± SD. Source data are provided as a Source Data file.

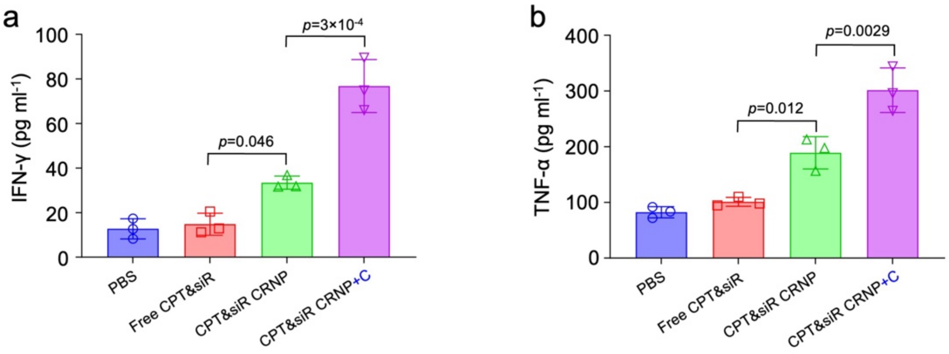

Supplementary Fig. 9 | Promotion of the secretion of pro-inflammatory cytokines for boosting the antitumor immune response by CPT&siR CRNPs with cold treatment. a-b, Production of IFN-γ (**a**) and TNF-α (**b**) measured by ELISA after co-culturing T cells with bone marrow dendritic cells (BMDCs, $CD11c^{+}CD86^{+}$) matured by EO771-OVA cells for 24 h (n=3 independent experiments). The EO771-OVA cells were pretreated with PBS, free CPT&siR, CPT&siR CRNPs, or CPT&siR CRNPs+C. "+C" represents cold treatment at -20 °C for 10 min. Statistical analyses were performed using one-way ANOVA with Tukey's multiple comparisons and correction. Data are presented as mean ± SD (**a-b**). Source data are provided as a Source Data file.

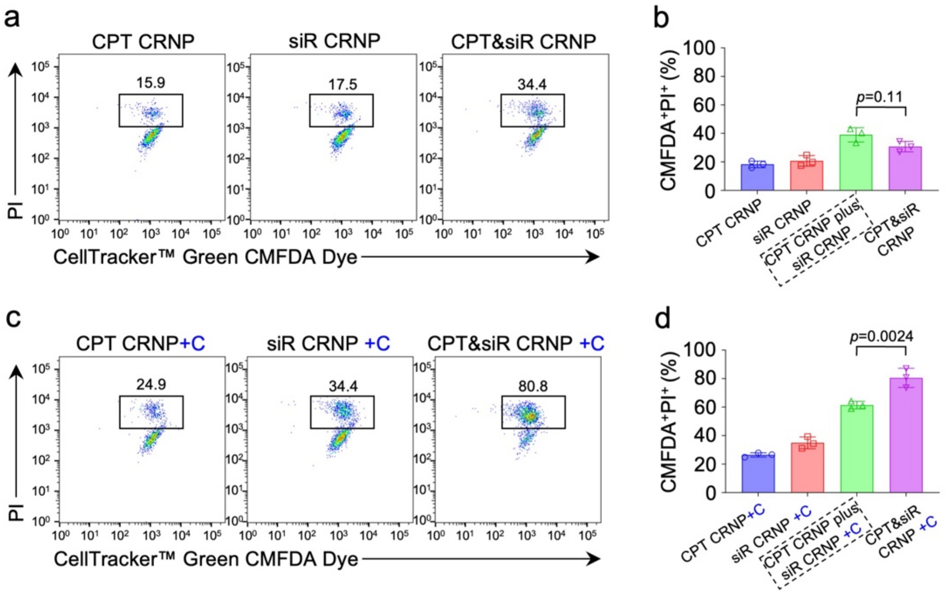

Supplementary Fig. 10 | Synergistic effect of CPT and siR co-delivered in CPT&siR CRNPs with cold treatment. a-b, Typical flow cytometry plots (**a**) and quantitative data (**b**) of dead EO771-OVA cells after T cell attacking for experiment without cold treatment. T cells were activated by BMDCs co-cultured with EO771-OVA cells with treatment of CPT CRNPs, siR CRNPs, or CPT&siR CRNPs (n=3 independent experiments). PI: propidium iodide. The group labeled with a dotted box represents the sum value (i.e., simple additive effect on cancer cell death) of CPT CRNPs and siR CRNPs. **c-d**, Typical flow cytometry plots (**c**) and quantitative data (**d**) of dead EO771-OVA cells after T cell attacking for experiment with cold treatment (+C) (n=3 independent experiments). T cells were activated by BMDCs co-cultured with EO771-OVA cells with treatment of CPT CRNPs+C, siR CRNPs+C, or CPT&siR CRNPs+C. The group labeled with dotted box represents the sum value (i.e., simple additive effect on cancer cell death) of CPT CRNPs+C and siR CRNPs+C. Statistical analyses were done using one-way ANOVA with Tukey's multiple comparisons and correction. Data are presented as mean ± SD (**b**, **d**). Source data are provided as a Source Data file.

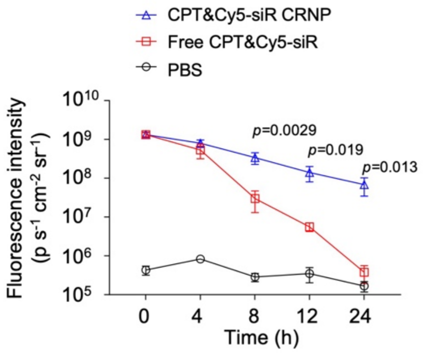

Supplementary Fig. 11 | CPT&Cy5-siR CRNPs extend the blood circulation time after injection. Blood was collected at 0, 4, 8, 12, and 24 h after intravenously injecting the mice with PBS, free CPT&Cy5-siR, and CPT&Cy5-siR CRNPs (n=3 mice). One-way ANOVA with Tukey's multiple comparisons and correction was used for statistical analyses. Data are presented as mean ± SD. Source data are provided as a Source Data file.

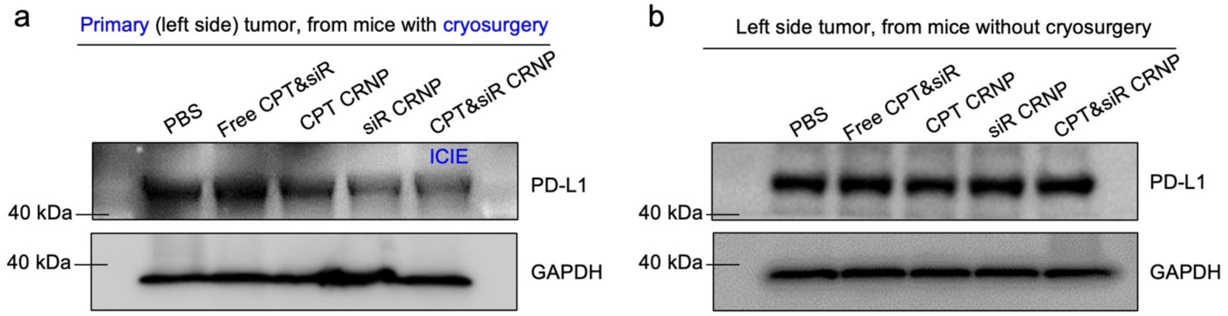

Supplementary Fig. 12 | ICIE enhances the in vivo PD-L1 silencing efficacy. a, Representative western blot results showing that ICIE induces better PD-L1 silencing in vivo among all primary tumors of mice receiving cryosurgery and injections of one of the various formulations including PBS, free CPT&siR, CPT CRNPs, siR CRNPs, and CPT&siR CRNPs. **b**, PD-L1 silencing in left tumors is not evident in the absence of cryosurgery. PD-L1: programmed death-ligand 1. Source data are provided as a Source Data file. All the experiments were repeated three times independently with similar results.

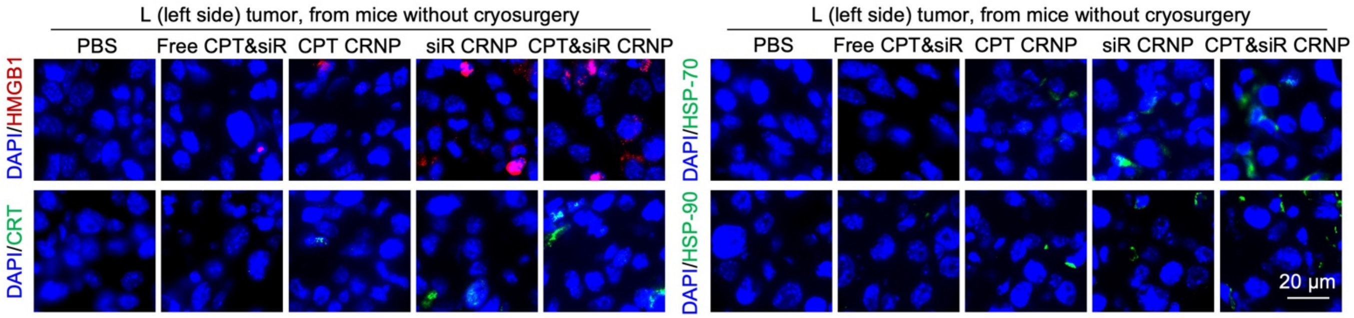

Supplementary Fig. 13 | CPT&siR CRNPs without cryosurgery induce minimal expression of DAMPs in TME. Representative immunofluorescence images of HMGB1, CRT, HSP-70, and HSP-90 expression in left tumors harvested from mice following treatments with PBS, free CPT&siR, CPT CRNPs, siR CRNPs, and CPT&siR CRNPs in the absence of cryosurgery. DAMP: damage-associated molecular patterns, TME: tumor microenvironment, HMGB1: high mobility group box protein 1, CRT: calreticulin, and HSP: heat shock protein. The experiments were repeated three times independently (n=3 mice) with similar results.

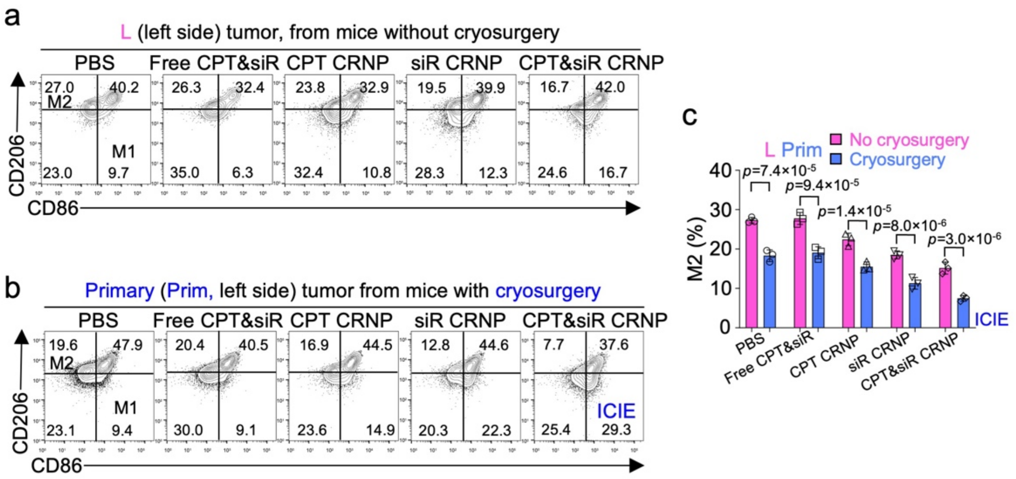

Supplementary Fig. 14 | ICIE reverses the immunologically "cold" TME by decreasing the frequency of M2 and increasing the M1 frequency. a-b, Representative flow cytometry plots of tumor associated macrophages (TAMs) M1 (F4/80$^+$CD206$^-$CD86$^+$) and M2 (F4/80$^+$CD206$^+$CD86$^-$) in left (**a**) or primary (**b**) tumors harvested from mice following treatments with PBS, free CPT&siR, CPT CRNPs, siR CRNPs, and CPT&siR CRNPs without (a) or with (b) cryosurgery (n=3 mice). **c-d**, Percentage of M2 (**c**) and M1 (**d**) in left/primary tumors from mice injected with various formulations without/with cryosurgery (n=3 mice). Two-way ANOVA with Sidak's post-test and correction for multiple comparisons was used for statistical analyses. Data are presented as mean ± SD (**c-d**). Source data are provided as a Source Data file.

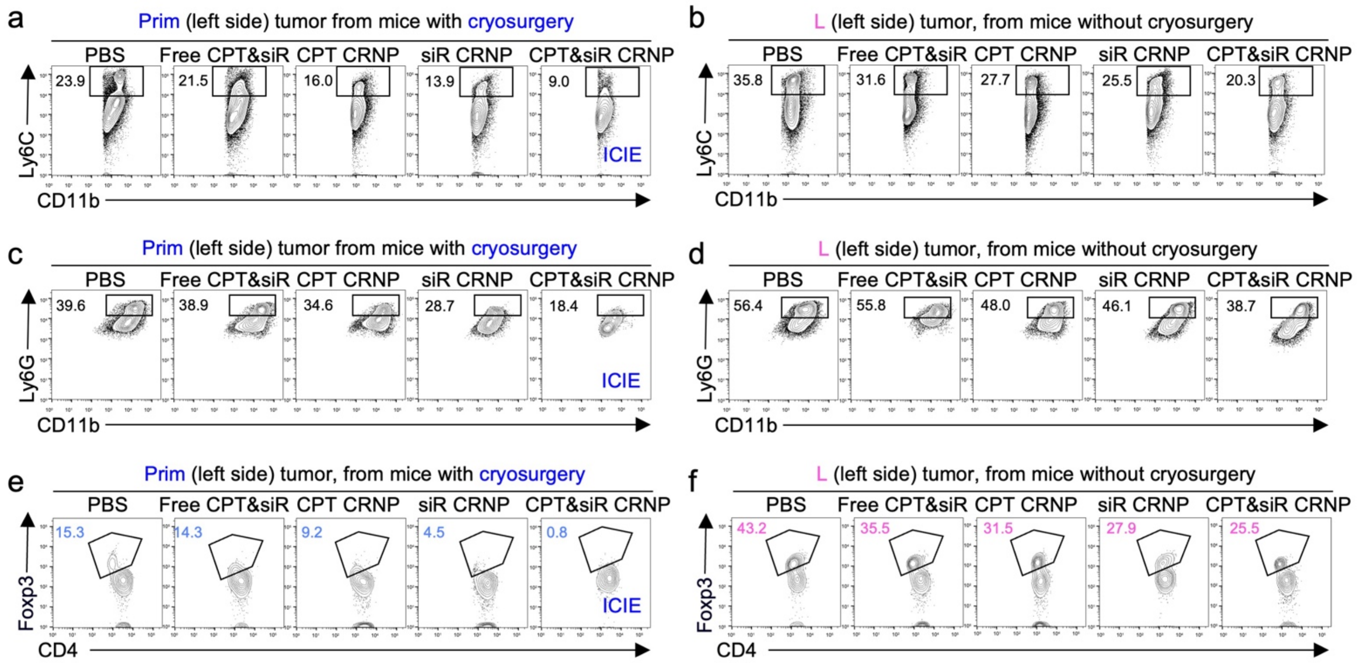

Supplementary Fig. 15 | ICIE reverses the immunologically "cold" TME by decreasing the frequency of M-MDSCs, PMN-MDSCs, and Treg cells in primary tumors. a-b, Representative flow cytometry plots of monocytic myeloid-derived suppressor cells (M-MDSC, $CD11b^+Ly6C^+Ly6G^-$) in primary (**a**) or left (**b**) tumors harvested from mice injected with PBS, free CPT&siR, CPT CRNPs, siR CRNPs, and CPT&siR CRNPs in the presence (**a**) or absence (**b**) of cryosurgery (n=3 mice). **c-d**, Representative flow cytometry plots of polymorphonuclear MDSCs (PMN-MDSC, $CD11b^+Ly6C^-Ly6G^+$) in primary (**c**) or left (**d**) tumors harvested from mice injected with the various formulations mentioned above in the presence (c) or absence (d) of cryosurgery (n=3 mice). **e-f**, Representative flow cytometry plots of regulatory T (Treg) cells ($CD4^+Foxp3^+$) in primary (**e**) or left (**f**) tumors harvested from mice injected with the various formulations mentioned above in the presence (**e**) or absence (**f**) of cryosurgery (n=3 mice).

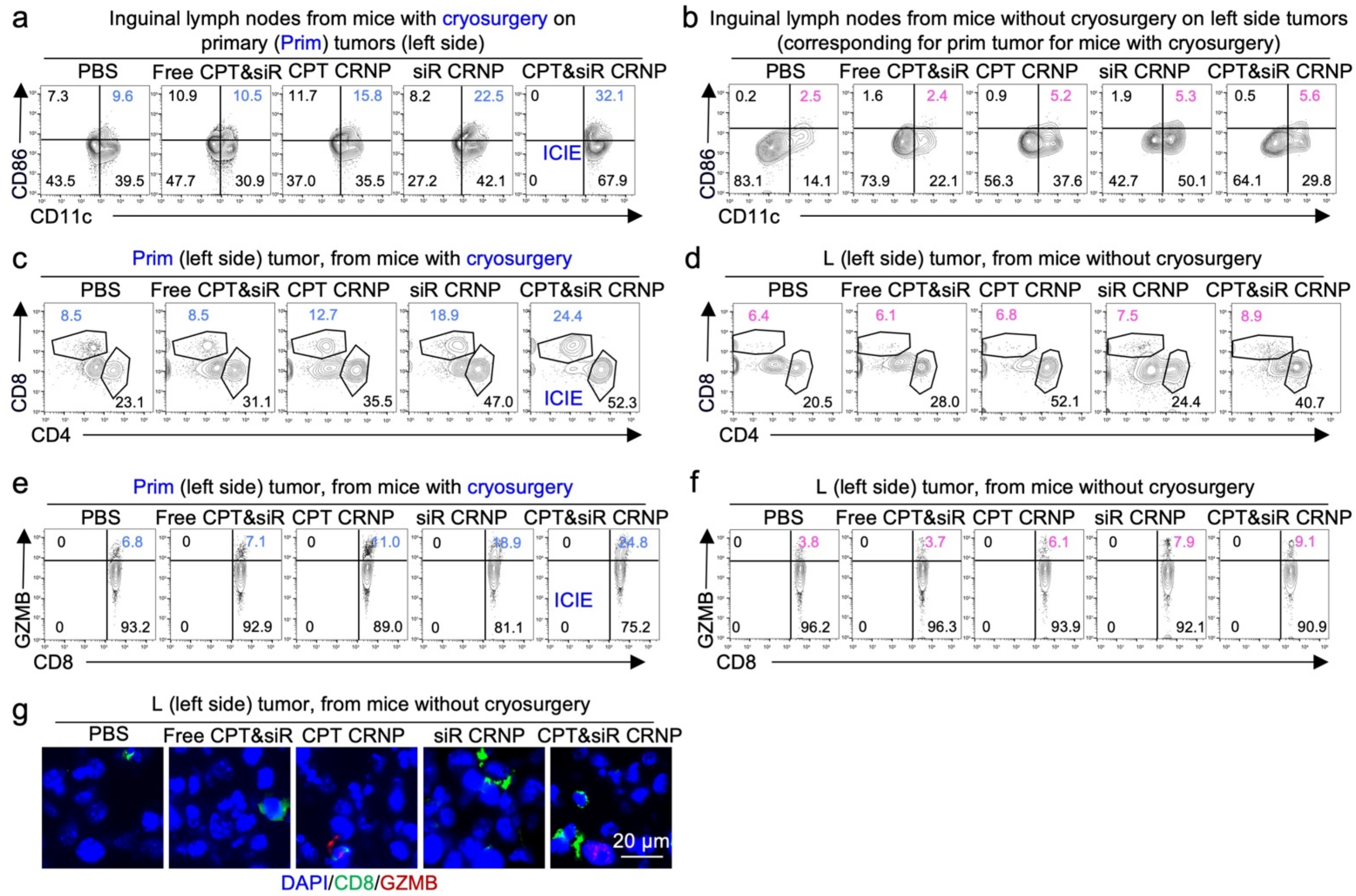

Supplementary Fig. 16 | ICIE induces strong antitumor immune response in primary tumors. a-b, Representative flow cytometry plots of mature dendritic cells (DCs, $CD11c^+CD86^+$) in inguinal lymph nodes of mice injected with PBS, free CPT&siRs, CPT CRNPs, siR CRNPs, and CPT&siR CRNPs in the presence (**a**) or absence (**b**) of cryosurgery (n=3 mice). **c-d**, Representative flow cytometry plots of $CD4^+$ and $CD8^+$ T cells in the primary (**c**) or left-side (**d**) tumors harvested from mice injected with various formulations in the presence (c) or absence (d) of cryosurgery (n=3 mice). **e-f**, Representative flow cytometry plots of infiltrated cytotoxic T lymphocytes (CTLs, $CD8^+GZMB^+$) in the primary (**e**) or left-side (**f**) tumors harvested from mice injected with various formulations mentioned above in the presence (**e**) or absence (**f**) of cryosurgery (n=3 mice). **g**, Representative fluorescence staining of infiltrated CTLs ($CD8^+GZMB^+$) in the left-side tumors harvested from mice injected with various formulations in the absence of cryosurgery. The experiments were repeated three times independently (n=3 mice) with similar results. GZMB: granzyme B.

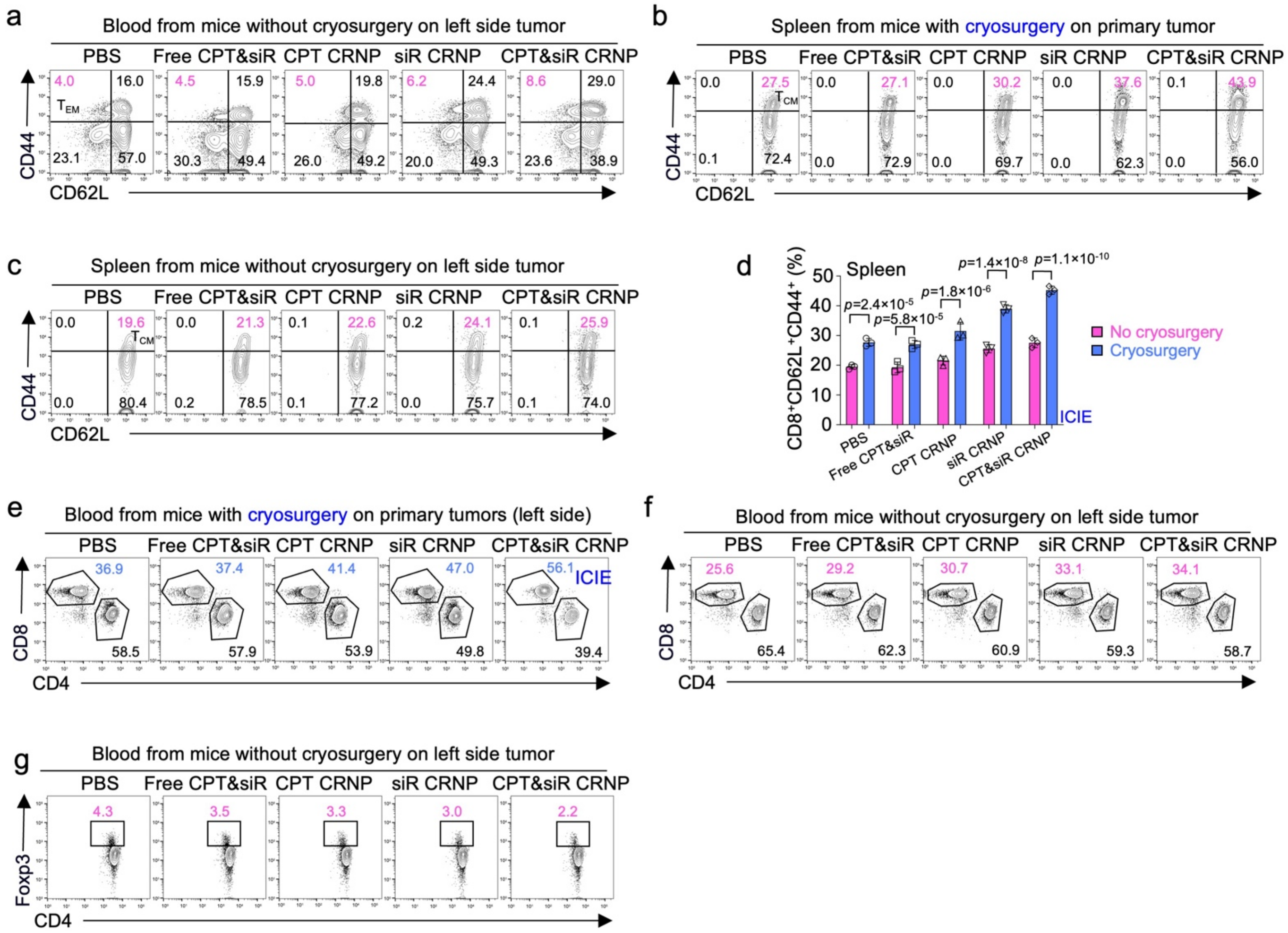

Supplementary Fig. 17 | ICIE enhances the frequency of effector/central memory $CD8^+$ T cells and increases the $CD8^+$/Treg cell ratios in blood/spleen. a, Representative flow cytometry plots of effector memory T cells (T_{EM}, $CD3^+CD8^+CD44^+CD62L^-$) in blood collected from mice injected with PBS, free CPT&siR, CPT CRNPs, siR CRNPs and CPT&siR CRNPs in the absence of cryosurgery (n=3 mice). **b-c**, Representative flow cytometry plots of central memory T cells (T_{CM}, $CD3^+CD8^+CD44^+CD62L^+$) in the spleen of mice injected with one of the aforementioned formulations in the presence (**b**) or absence (**c**) of cryosurgery. **d**, Quantitative data of T_{CM} in the spleen of mice received treatment with one of the aforementioned formulations in the presence or absence of cryosurgery (n=3 mice). Statistical analyses were done using two-way ANOVA with Sidak's post-test and correction for multiple comparisons. Data are presented as mean ± SD. **e-f**, Representative flow cytometry plots of $CD4^+$ and $CD8^+$ T cells in blood harvested from mice injected with one of the various formulations in the presence (**e**) or absence (**f**) of cryosurgery (n=3 mice). **g**, Representative flow cytometry plots of Treg cells ($CD4^+Foxp3^+$) in the blood collected from mice injected with the various formulations in the absence of cryosurgery (n=3 mice). Source data are provided as a Source Data file.

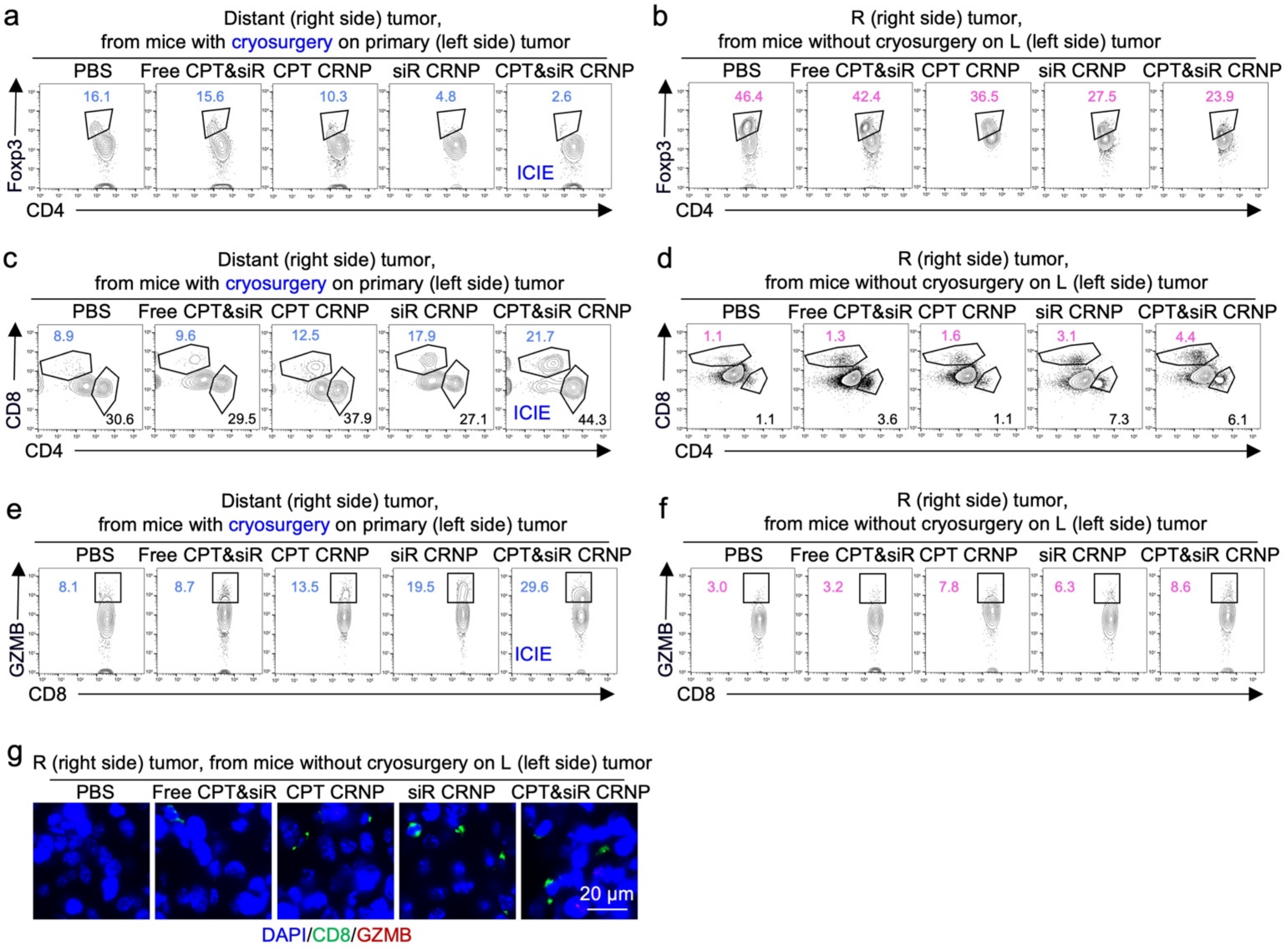

Supplementary Fig. 18 | ICIE stimulates strong antitumor immune response in distant tumors. a-b, Representative flow cytometry plots of Treg cells ($CD4^+Foxp3^+$) in distant (with cryosurgery on primary tumor)/right-side (without cryosurgery) tumors harvested from mice injected with PBS, free CPT&siR, CPT CRNPs, siR CRNPs and CPT&siR CRNPs in the presence (**a**) or absence (**b**) of cryosurgery (on primary tumor) (n=3 mice). **c-d**, Representative flow cytometry plots of $CD4^+$ and $CD8^+$ T cells in distant/right-side tumors harvested from mice injected with various formulations in the presence (**c**) or absence (**d**) of cryosurgery (on primary tumor) (n=3 mice). **e-f**, Representative flow cytometry plots of infiltrated CTLs ($CD8^+GZMB^+$) in the distant/right-side tumors harvested from mice injected with various formulations in the presence (**e**) or absence (**f**) of cryosurgery (on primary tumor) (n=3 mice). **g**, Representative immunofluorescence staining of infiltrated CTL ($CD8^+GZMB^+$) in the right tumors harvested from mice injected with various formulations in the absence of cryosurgery. The experiments were repeated three times independently (n=3 mice) with similar results.

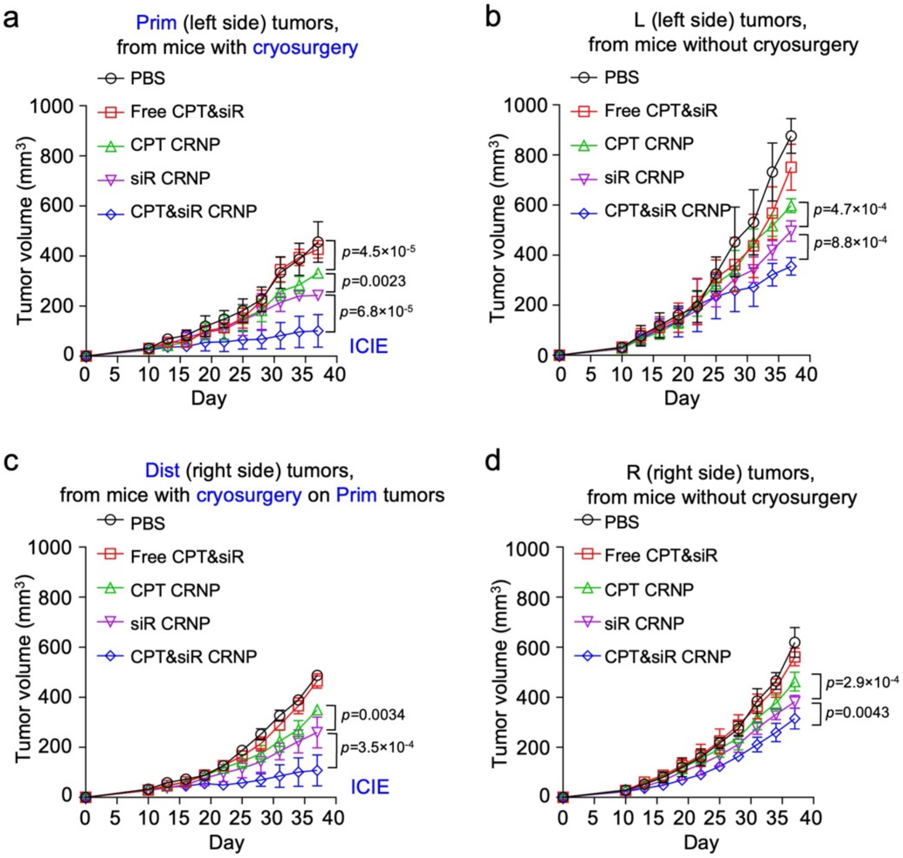

Supplementary Fig. 19 | ICIE inhibits the growth of both primary and distant tumors. a-b, Primary (with cryosurgery, a)/left-side (without cryosurgery, b) tumor growth curves in mice injected with PBS, free CPT&siR, CPT CRNPs, siR CRNPs, and CPT&siR CRNPs in the presence (**a**) or absence (**b**) of cryosurgery (n=6 mice). **c-d**, Distant (with cryosurgery on primary tumor, **c**)/right-side (without cryosurgery, **d**) tumor growth curves in mice injected with various formulations in the presence or absence of cryosurgery (on primary tumor) (n=6 mice). Two-way ANOVA with Sidak's post-test and correction for multiple comparisons was used for statistical analyses. Data are presented as mean ± SD (**a**-**d**). Source data are provided as a Source Data file.

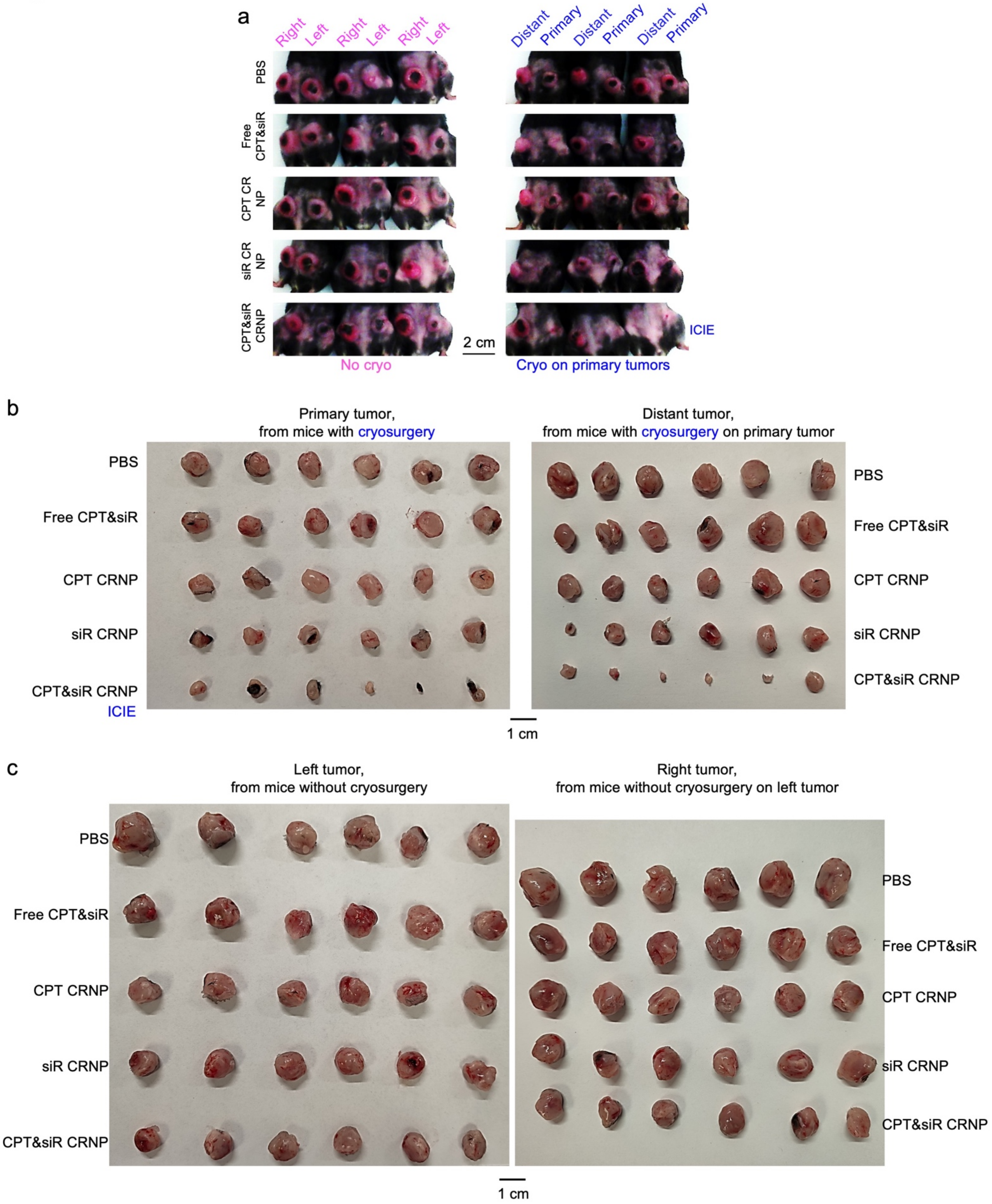

Supplementary Fig. 20 | Images of tumors showing ICIE reduces the size of both primary and distant tumors. a, Representative photographs of EO771 tumor-bearing mice at the end of the study, showing effective tumor destruction by the ICIE treatment. The black regions are a result of skin wound that often occurs in the nipple area due to orthotopic tumor growth. The experiments were repeated six

times independently (n=6 mice) with similar results. **b**, Images of primary and distant tumors collected on the final day of the study. Mice in different groups were treated with cryosurgery and injections of PBS, free CPT&siR, CPT CRNPs, siR CRNPs, and CPT&siR CRNPs (n=6 mice). **c**, Images of left and right tumors collected on the final day of the study. Mice were injected with the various formulations without cryosurgery (n=6 mice).

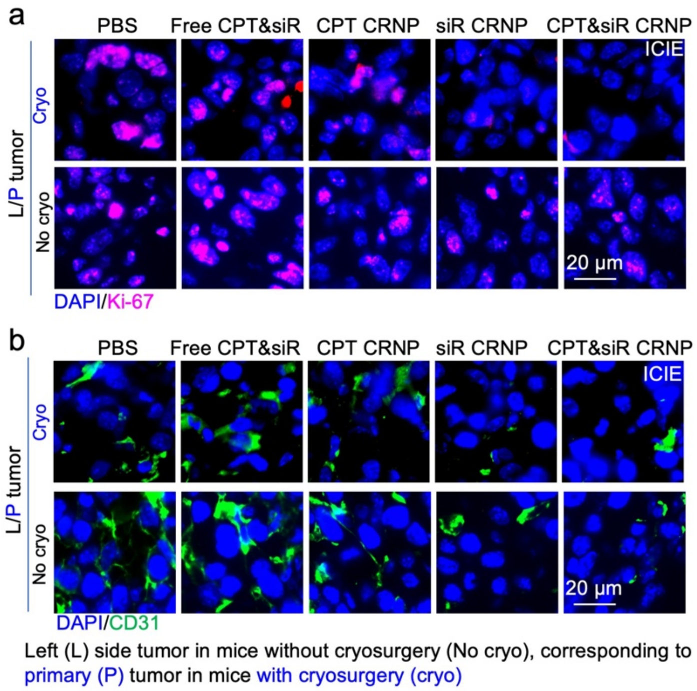

Supplementary Fig. 21 | ICIE significantly decreases the expression of Ki-67 and CD31 in tumors. **a**, Representative immunofluorescence staining of Ki-67 in the primary (P, with cryosurgery)/left-side (L, without cryosurgery) tumor sections collected from mice injected with PBS, free CPT&siR, CPT CRNPs, siR CRNPs, and CPT&siR CRNPs in the presence or absence of cryosurgery. **b**, Representative immunofluorescence staining of CD31 in the primary (P, with cryosurgery)/left-side (L, without cryosurgery) tumor sections collected from mice injected with various formulations in the presence or absence of cryosurgery. All the experiments were repeated three times independently (n=3 mice) with similar results.

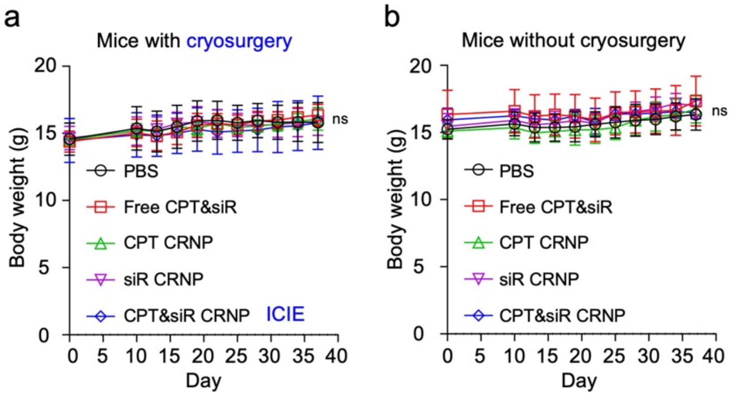

Supplementary Fig. 22 | ICIE induces no significant changes in body weight. a, Body weight recorded for mice injected with PBS, free CPT&siR, CPT CRNPs, siR CRNPs, and CPT&siR CRNPs in the presence of cryosurgery (n=6 mice). **b**, Body weight recorded for mice injected with various formulations in the absence of cryosurgery (n=6 mice). Two-way ANOVA with Sidak's post-test and correction for multiple comparisons was used for statistical analyses. ns: not significance. Data are presented as mean ± SD (**a-b**). Source data are provided as a Source Data file.

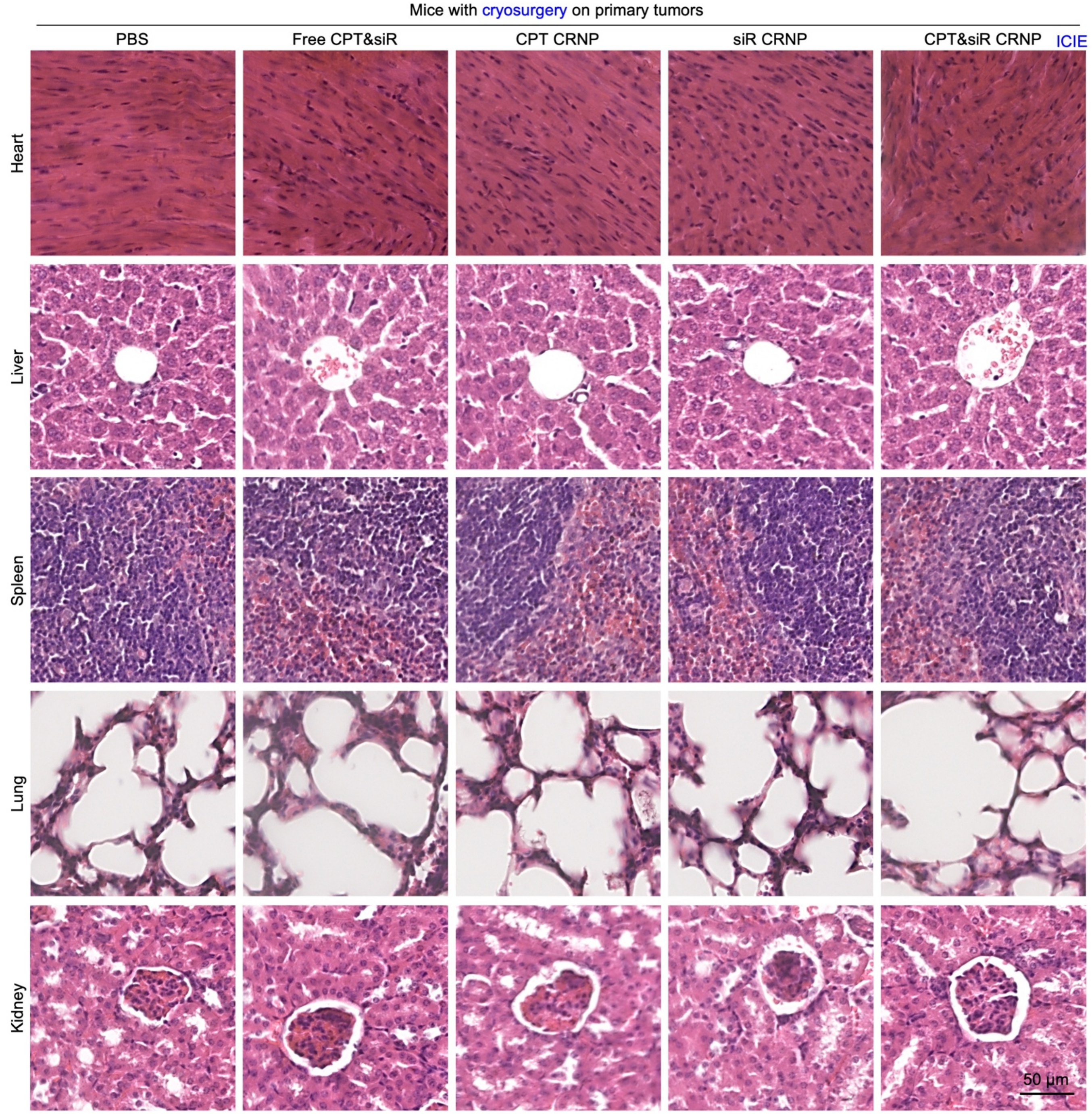

Supplementary Fig. 23 | ICIE induces no evident damage in major organs. H&E staining of major organs including heart, liver, spleen, lung, and kidney collected from mice injected with PBS, free CPT&siR, CPT CRNPs, siR CRNPs, and CPT&siR CRNPs in the presence of cryosurgery. H&E: hematoxylin and eosin. The experiments were repeated three times independently (n=3 mice) with similar results.

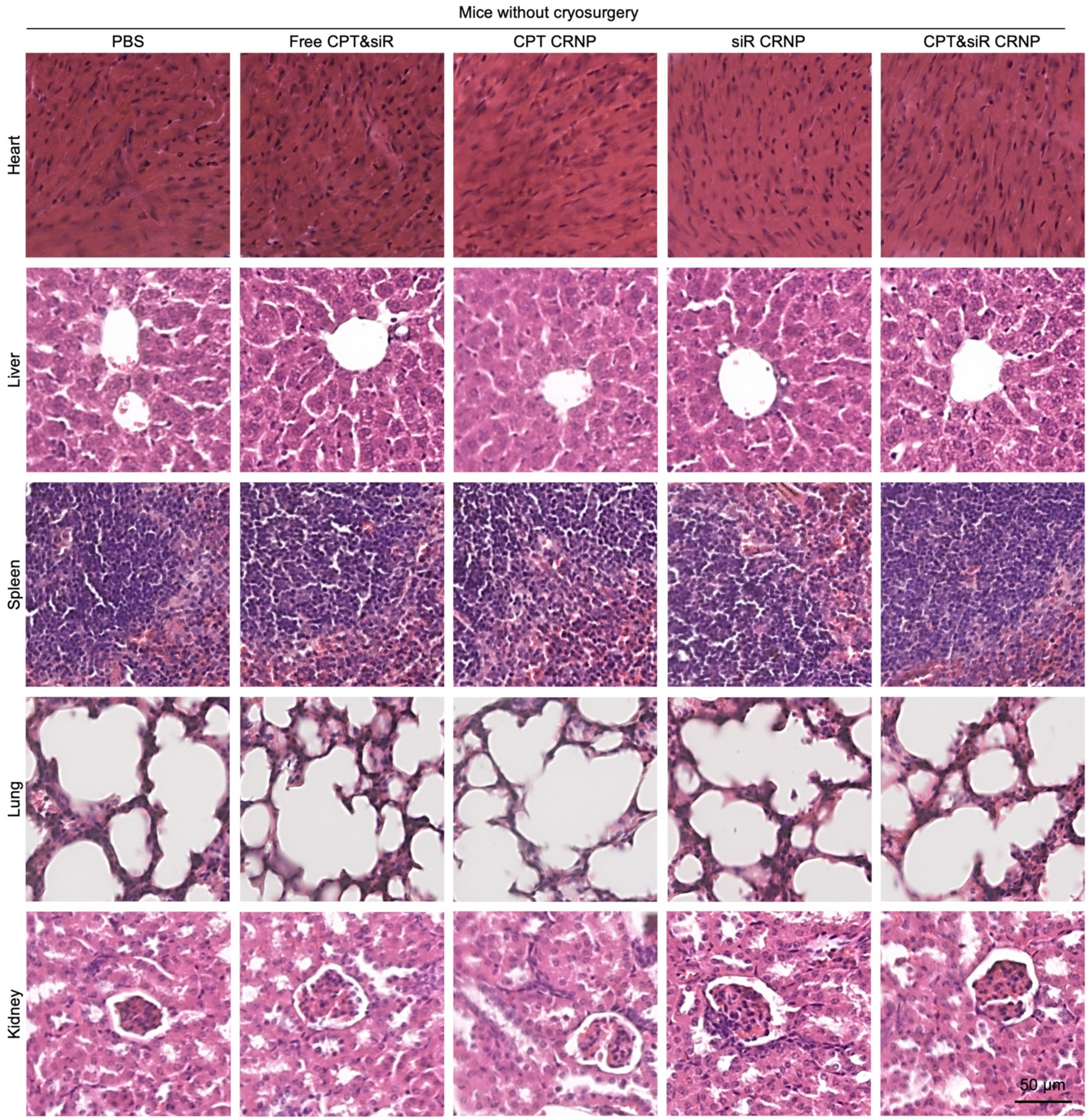

Supplementary Fig. 24 | CRNPs induce no evident damages in major organs. H&E staining of major organs including heart, liver, spleen, lung, and kidney collected from mice injected with PBS, free CPT&siR, CPT CRNPs, siR CRNPs, and CPT&siR CRNPs in the absence of cryosurgery. The experiments were repeated three times independently (n=3 mice) with similar results.

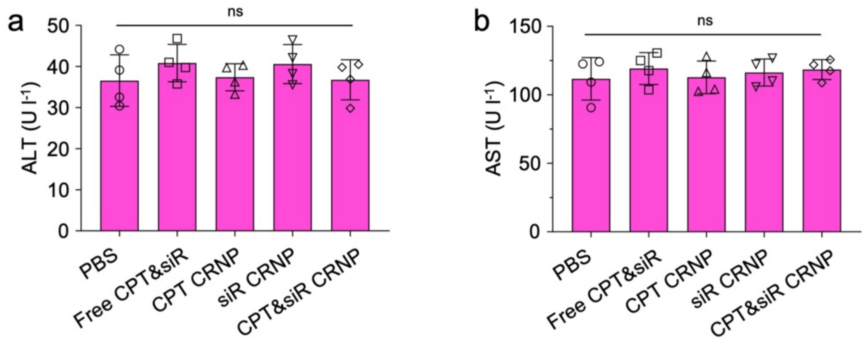

Supplementary Fig. 25 | CRNPs induce no evident changes in blood alanine aminotransferase (ALT) and aspartate aminotransferase (AST) levels. a-b, Detection of ALT (**a**) and AST (**b**) levels in the blood of mice injected with PBS, free CPT&siR, CPT CRNPs, siR CRNPs, and CPT&siR CRNPs at the end of the study (n=4 mice). Statistical analyses were performed using one-way ANOVA with Tukey's multiple comparisons and correction. ns: not significant. Data are presented as mean ± SD (**a**-**b**). Source data are provided as a Source Data file.

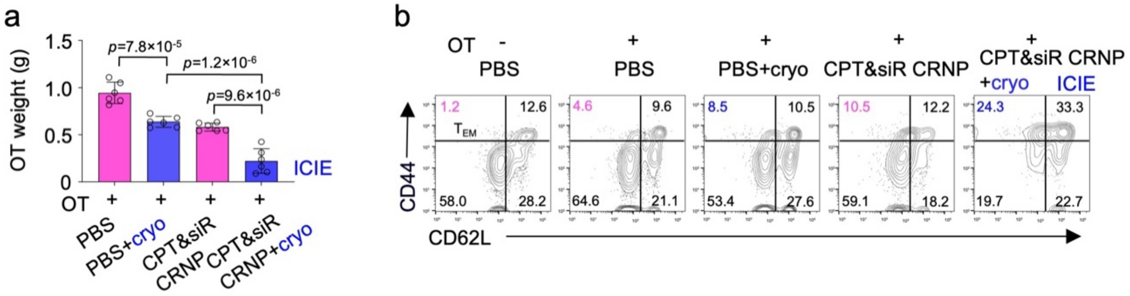

Supplementary Fig. 26 | ICIE effectively inhibits the growth of orthotopic tumors (OTs) and generates long-term antitumor memory immune response against metastatic tumors. a, Weight of OTs obtained after sacrificing the mice at the end of the study, showing effective destruction of localized OTs by the ICIE treatment (n=6 mice). Mice with OTs were treated with PBS, PBS+cryo, CPT&siR CRNPs, or CPT&siR CRNPs+cryo. Cryosurgery (cryo) was performed on the OT at 8 h after the first injection of the various formulations. **b**, Representative flow cytometry plots of effector memory T cells (T_{EM}, $CD3^+CD8^+CD44^+CD62L^-$) in blood collected from mice with no OT injected with PBS, and mice with OT treated with PBS, PBS+cryo, CPT&siR CRNPs, or CPT&siR CRNPs+cryo. Cryosurgery was performed on the OT at 8 h after the first injection of the various formulations (n=3 mice). Statistical analyses were performed using one-way ANOVA with Tukey's multiple comparisons and correction. Data are presented as mean ± SD (**a**). Source data are provided as a Source Data file.

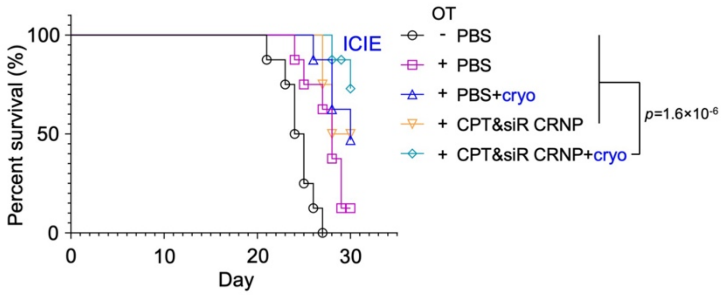

Supplementary Fig. 27 | ICIE effectively prolongs the overall survival of Balb/c mice with metastatic tumors. Overall survival rate of Balb/c mice with 4T1 lung metastasis in mice with no orthotopic tumor (OT) after treatment with PBS, and mice with OT after treating with PBS, PBS+cryo, CPT&siR CRNPs, or CPT&siR CRNPs+cryo (n=8 mice). Cryosurgery was performed on the OT at 8 h after the first injection of the various formulations. Statistical analyses were performed using the Log-rank (Mantel-Cox) test. Source data are provided as a Source Data file.
