# Supplementary material for: In-situ cryo-immune engineering of tumor microenvironment with cold-responsive nanotechnology for cancer immunotherapy
Source: Nat Commun. 2023 Jan 24;14:392. doi: 10.1038/s41467-023-36045-7 (PMC9873931; doi:10.1038/s41467-023-36045-7)
Supplement: Supplementary file 3 — Description of Additional Supplementary Files [file 41467_2023_36045_MOESM3_ESM.pdf]

Description of Additional Supplementary Files

Supplementary Movie 1: Tumor-attacking capability of the activated CD8+ T cells. CD8+ T cells activated by BMDCs co-cultured with EO771-OVA cells receiving ICIE treatment, migrate to and accumulate around the EO771-OVA tumor cells (labeled with green color) within 5 min to attack them persistently and the tumor cells are eventually dismantled within 4 h.

Supplementary Code: The supplementary code file contains a Matlab script to analyze the gray value (Grayscale value) data to observe the cold-responsive behavior of the samples. These scripts were developed for the following publication: " In-situ cryo-immune engineering of tumor microenvironment with coldresponsive nanotechnology for cancer immunotherapy" Ou et al., in Nature communication. These files allow to analyze the grayscale value data acquired on the Linkam temperature-controlled microscope stage mounted on a Zeiss Axio Scope A1 microscope (Oberkochen, Germany) to observe the cold-responsive behavior of the samples. To run these scripts, you need to install the Matlab programming environment first (download from: https://www.mathworks.com/?s_tid=gn_logo). The untreated raw data are provided in the attached files. More detailed instruction to run the data analysis is provided inside the Matlab script. The script was written and tested in MATLAB 2020b version (Windows 10), and other versions were not tested. This script will provide the temperature (T) and normalized grayscale value matrices to produce the graphs shown in Fig. 2d. The expected run time of the script is very short (<1 second).
