# Supplementary figures and images for: In-situ cryo-immune engineering of tumor microenvironment with cold-responsive nanotechnology for cancer immunotherapy (part 2 of 2)
Source: Nat Commun. 2023 Jan 24;14:392. doi: 10.1038/s41467-023-36045-7 (PMC9873931; doi:10.1038/s41467-023-36045-7)

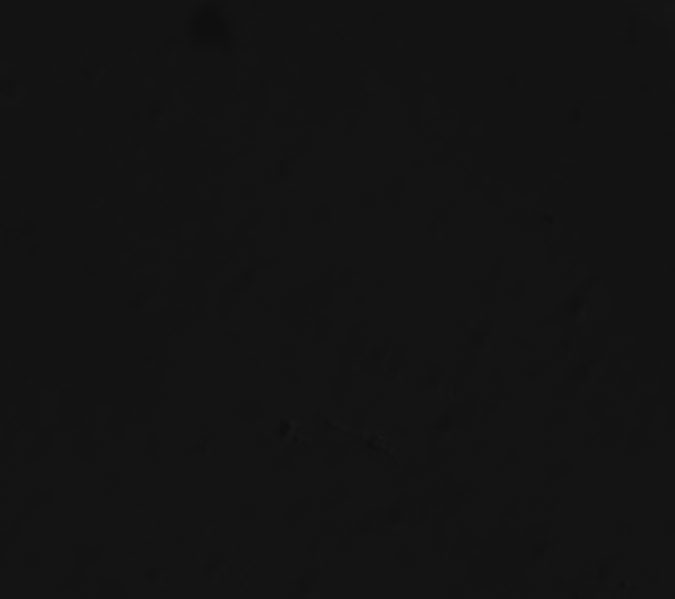

Supplement: Supplementary file 5 — Supplementary Code [file 41467_2023_36045_MOESM5_ESM.zip › Source Code/Untreated raw data for testing the code/PLGA NPs/Image64.jpg]

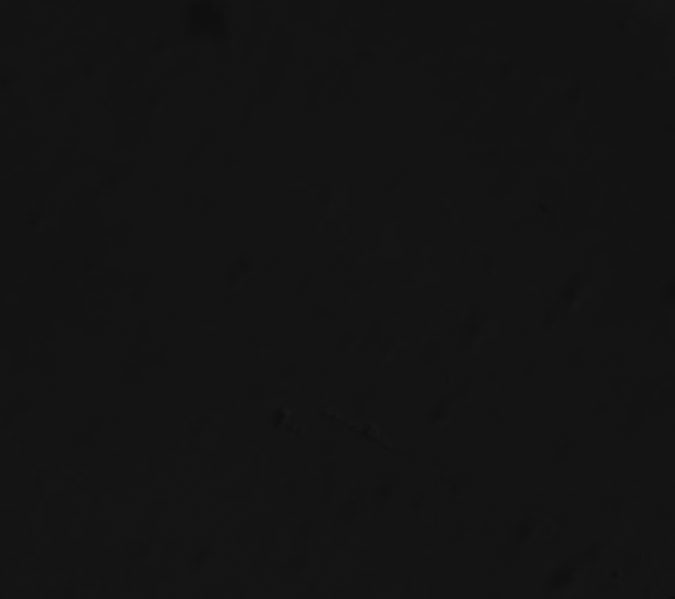

Supplement: Supplementary file 5 — Supplementary Code [file 41467_2023_36045_MOESM5_ESM.zip › Source Code/Untreated raw data for testing the code/PLGA NPs/Image279.jpg]

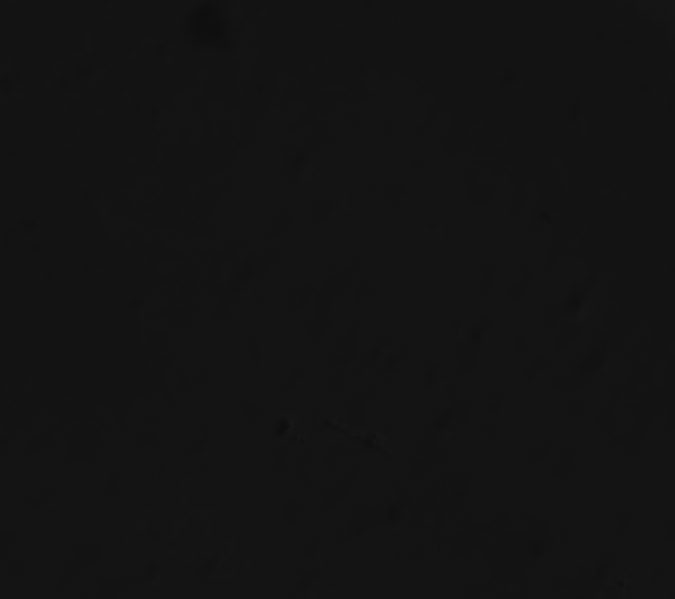

Supplement: Supplementary file 5 — Supplementary Code [file 41467_2023_36045_MOESM5_ESM.zip › Source Code/Untreated raw data for testing the code/PLGA NPs/Image70.jpg]

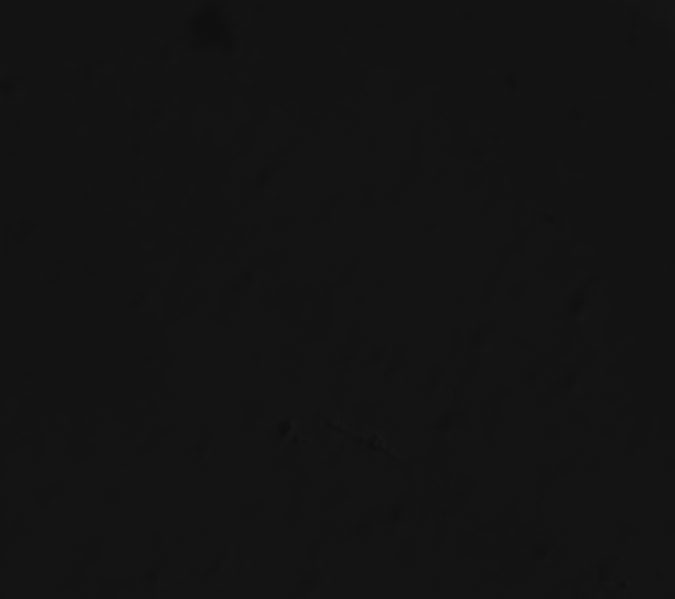

Supplement: Supplementary file 5 — Supplementary Code [file 41467_2023_36045_MOESM5_ESM.zip › Source Code/Untreated raw data for testing the code/PLGA NPs/Image58.jpg]

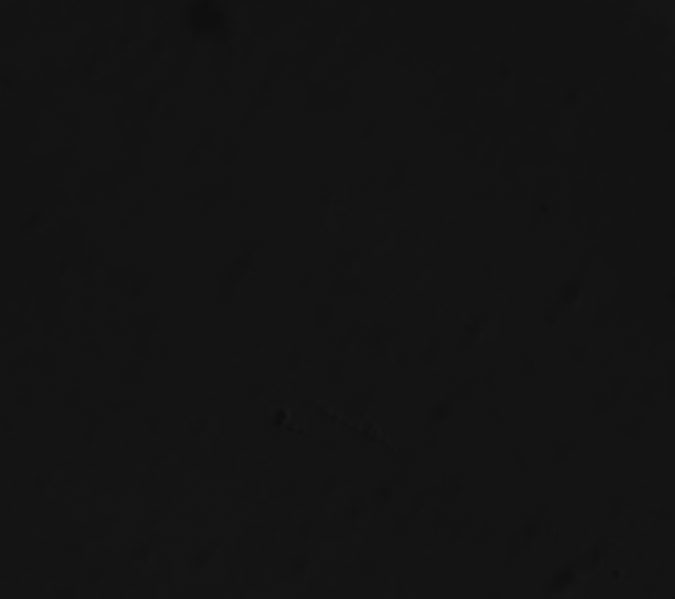

Supplement: Supplementary file 5 — Supplementary Code [file 41467_2023_36045_MOESM5_ESM.zip › Source Code/Untreated raw data for testing the code/PLGA NPs/Image245.jpg]

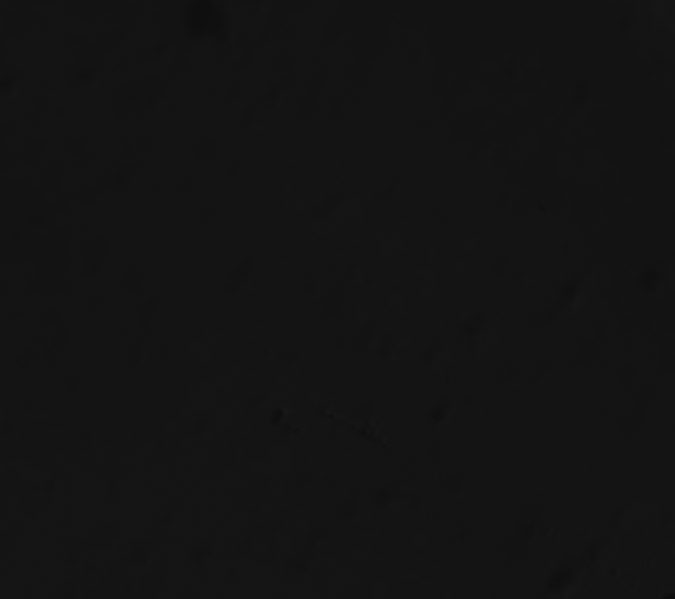

Supplement: Supplementary file 5 — Supplementary Code [file 41467_2023_36045_MOESM5_ESM.zip › Source Code/Untreated raw data for testing the code/PLGA NPs/Image523.jpg]

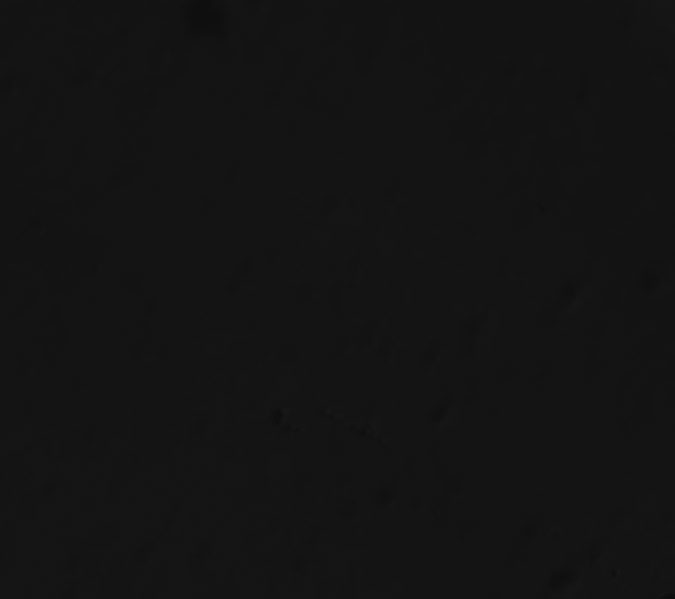

Supplement: Supplementary file 5 — Supplementary Code [file 41467_2023_36045_MOESM5_ESM.zip › Source Code/Untreated raw data for testing the code/PLGA NPs/Image537.jpg]

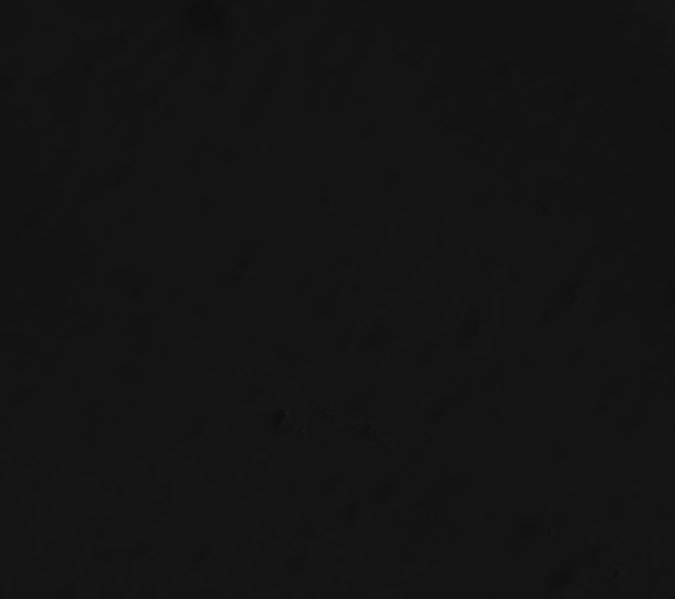

Supplement: Supplementary file 5 — Supplementary Code [file 41467_2023_36045_MOESM5_ESM.zip › Source Code/Untreated raw data for testing the code/PLGA NPs/Image251.jpg]

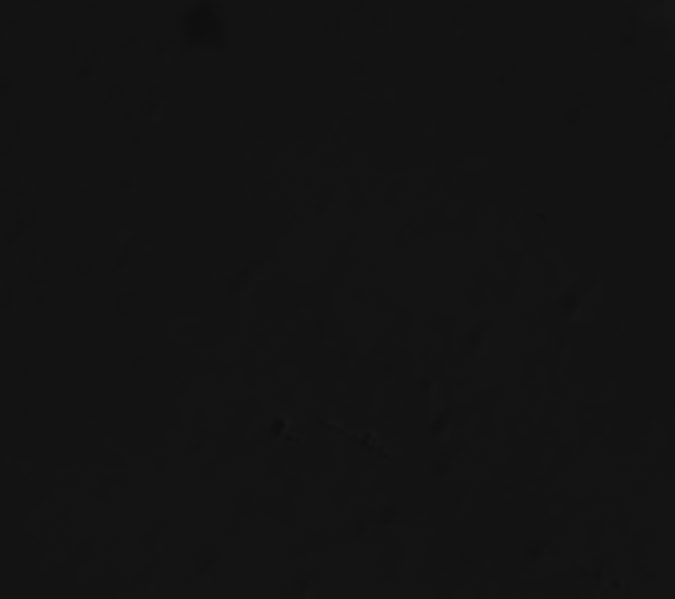

Supplement: Supplementary file 5 — Supplementary Code [file 41467_2023_36045_MOESM5_ESM.zip › Source Code/Untreated raw data for testing the code/PLGA NPs/Image9.jpg]

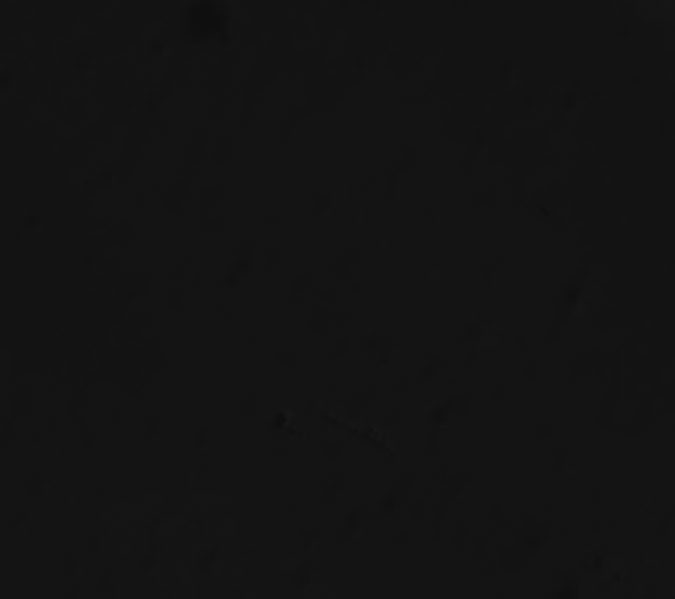

Supplement: Supplementary file 5 — Supplementary Code [file 41467_2023_36045_MOESM5_ESM.zip › Source Code/Untreated raw data for testing the code/PLGA NPs/Image133.jpg]

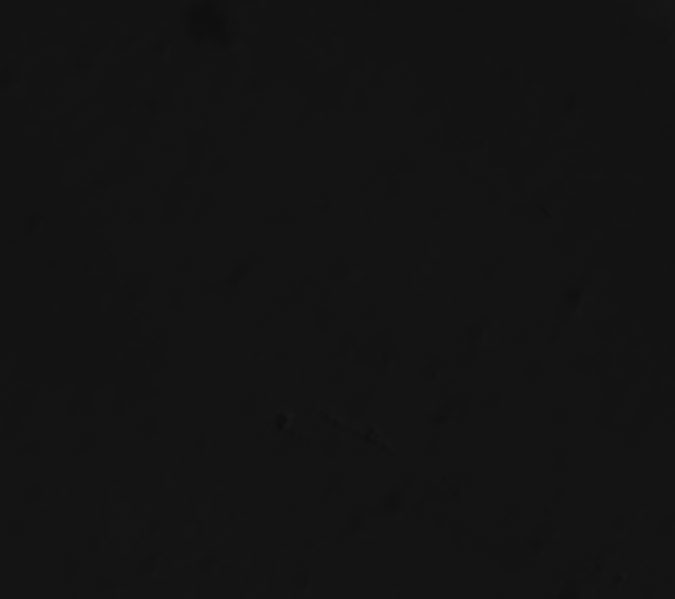

Supplement: Supplementary file 5 — Supplementary Code [file 41467_2023_36045_MOESM5_ESM.zip › Source Code/Untreated raw data for testing the code/PLGA NPs/Image127.jpg]

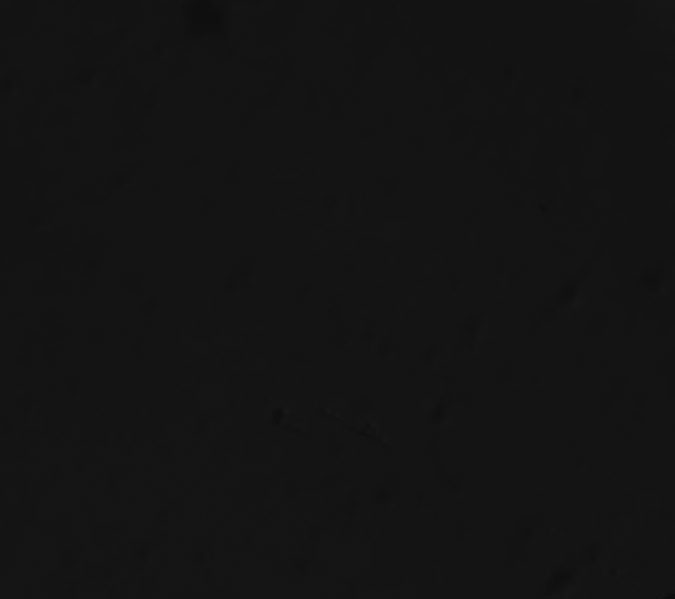

Supplement: Supplementary file 5 — Supplementary Code [file 41467_2023_36045_MOESM5_ESM.zip › Source Code/Untreated raw data for testing the code/PLGA NPs/Image494.jpg]

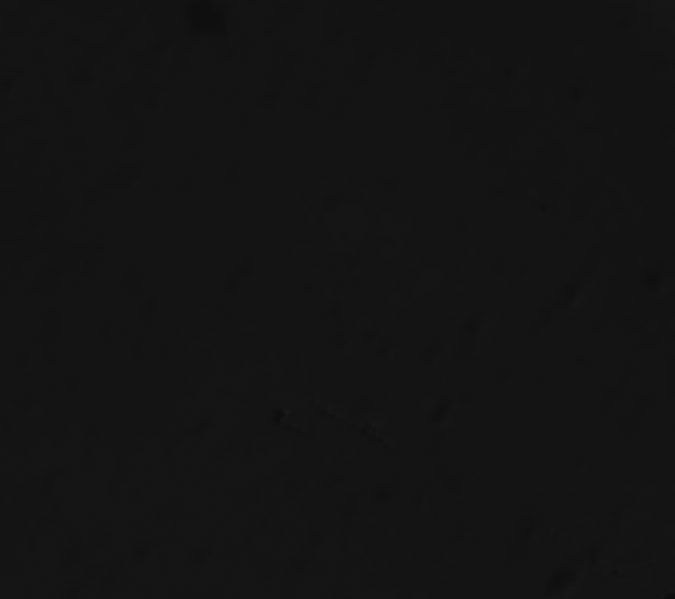

Supplement: Supplementary file 5 — Supplementary Code [file 41467_2023_36045_MOESM5_ESM.zip › Source Code/Untreated raw data for testing the code/PLGA NPs/Image480.jpg]

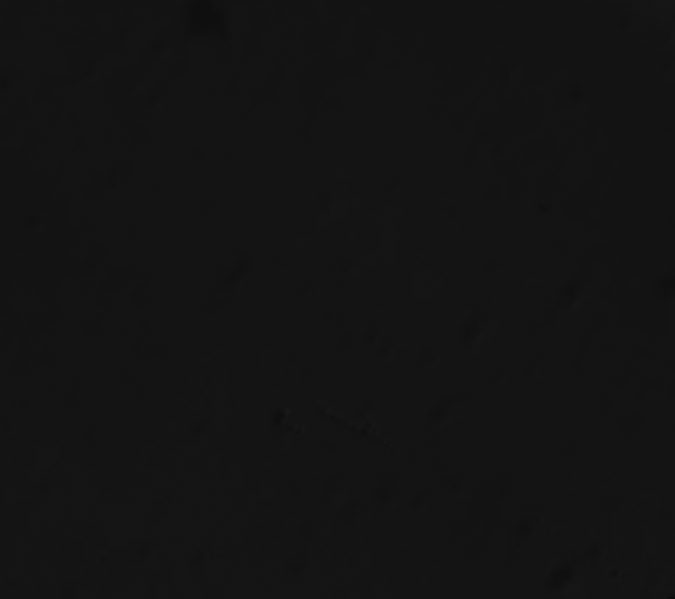

Supplement: Supplementary file 5 — Supplementary Code [file 41467_2023_36045_MOESM5_ESM.zip › Source Code/Untreated raw data for testing the code/PLGA NPs/Image319.jpg]

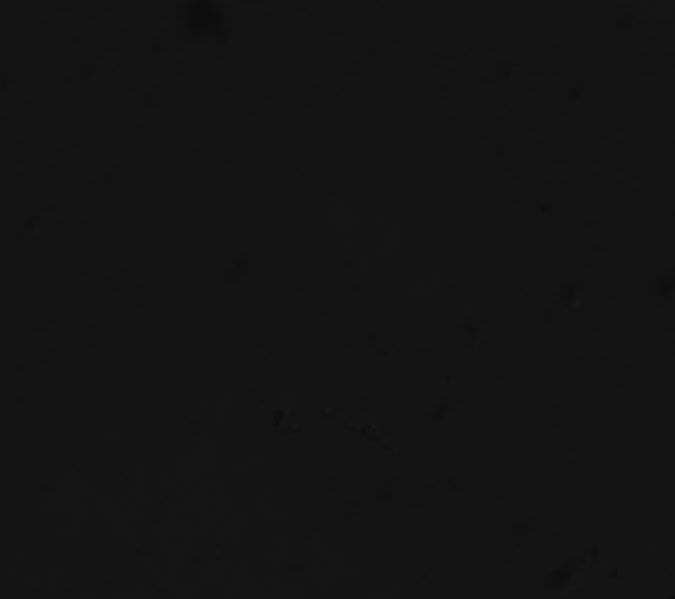

Supplement: Supplementary file 5 — Supplementary Code [file 41467_2023_36045_MOESM5_ESM.zip › Source Code/Untreated raw data for testing the code/PLGA NPs/Image331.jpg]

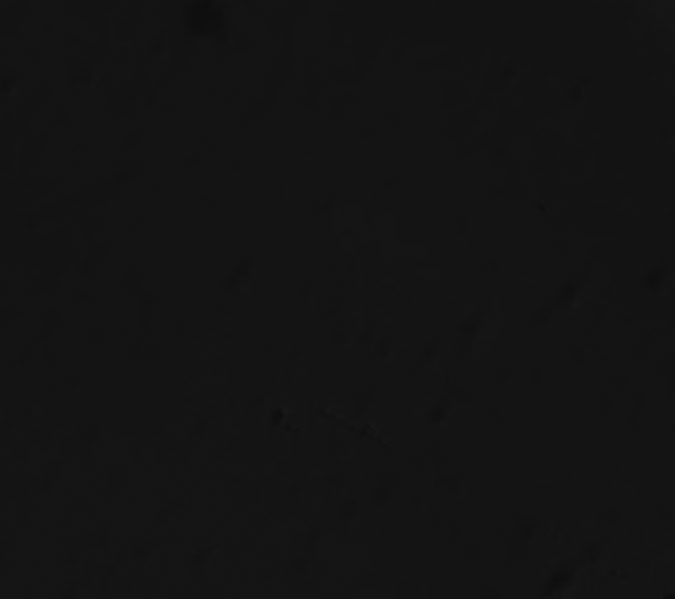

Supplement: Supplementary file 5 — Supplementary Code [file 41467_2023_36045_MOESM5_ESM.zip › Source Code/Untreated raw data for testing the code/PLGA NPs/Image457.jpg]

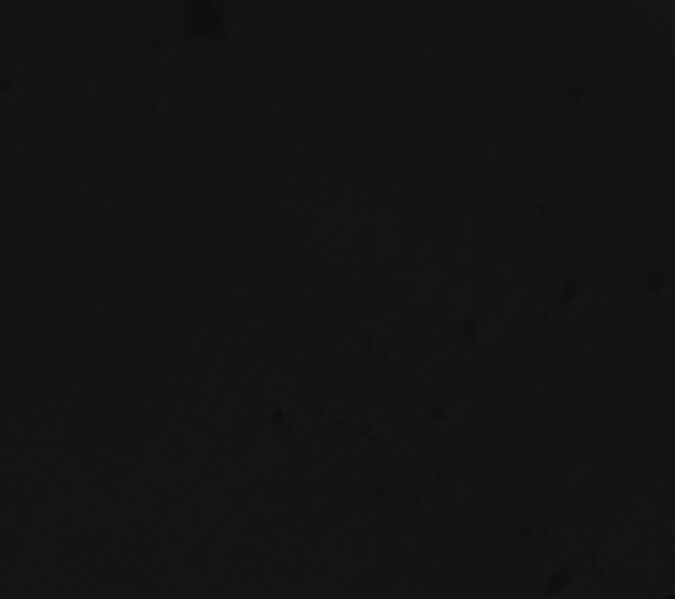

Supplement: Supplementary file 5 — Supplementary Code [file 41467_2023_36045_MOESM5_ESM.zip › Source Code/Untreated raw data for testing the code/PLGA NPs/Image443.jpg]

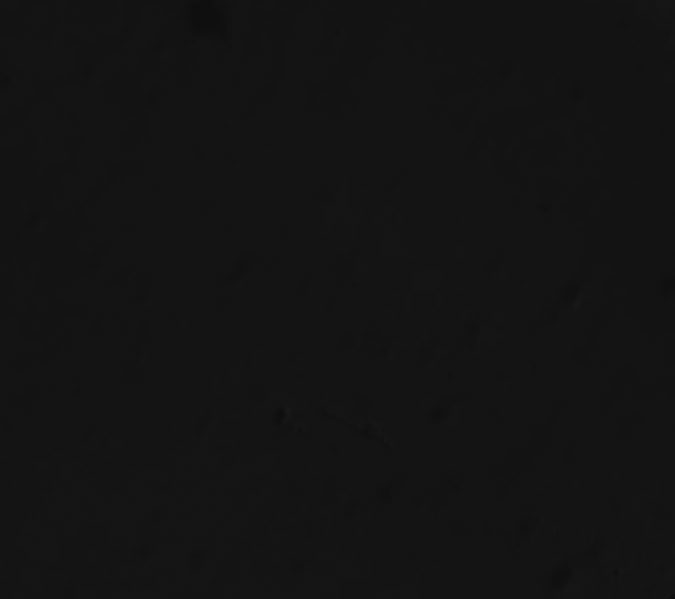

Supplement: Supplementary file 5 — Supplementary Code [file 41467_2023_36045_MOESM5_ESM.zip › Source Code/Untreated raw data for testing the code/PLGA NPs/Image325.jpg]

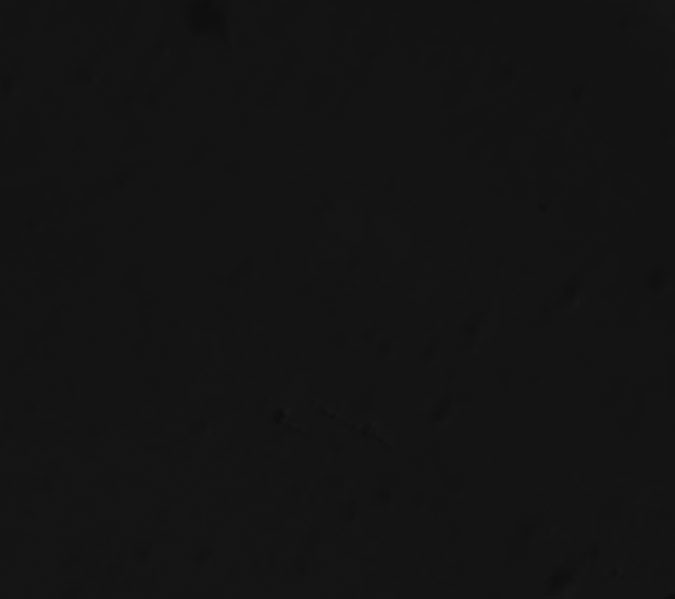

Supplement: Supplementary file 5 — Supplementary Code [file 41467_2023_36045_MOESM5_ESM.zip › Source Code/Untreated raw data for testing the code/PLGA NPs/Image455.jpg]

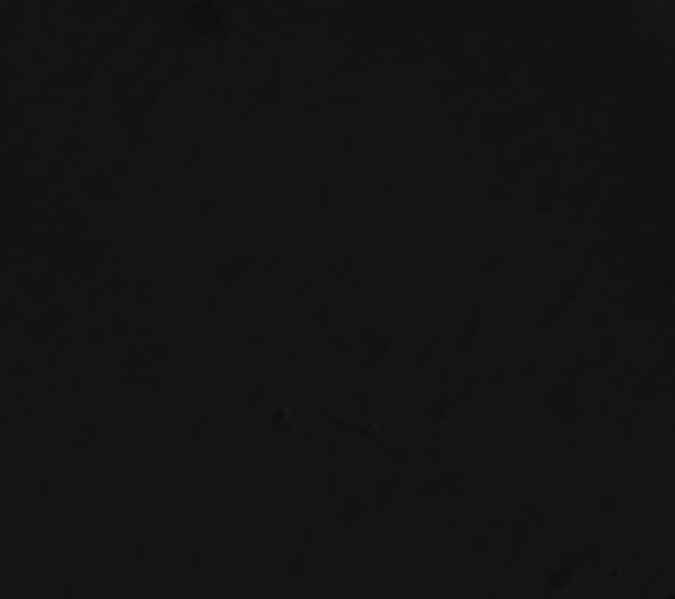

Supplement: Supplementary file 5 — Supplementary Code [file 41467_2023_36045_MOESM5_ESM.zip › Source Code/Untreated raw data for testing the code/PLGA NPs/Image333.jpg]

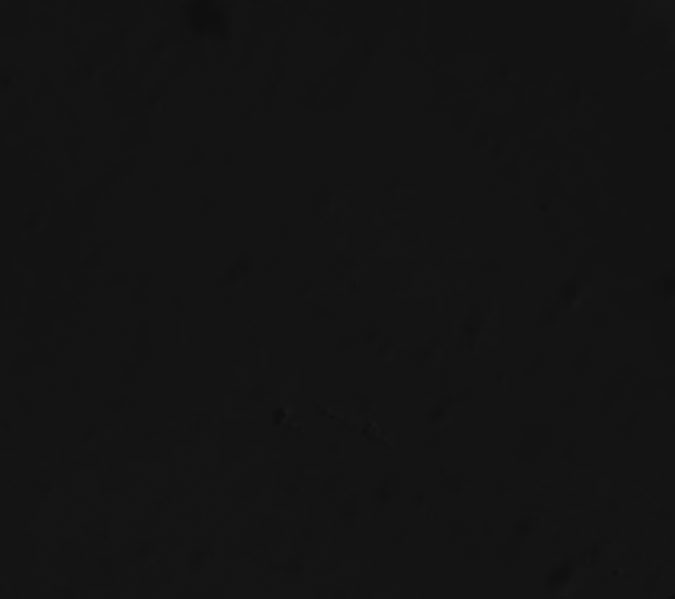

Supplement: Supplementary file 5 — Supplementary Code [file 41467_2023_36045_MOESM5_ESM.zip › Source Code/Untreated raw data for testing the code/PLGA NPs/Image327.jpg]

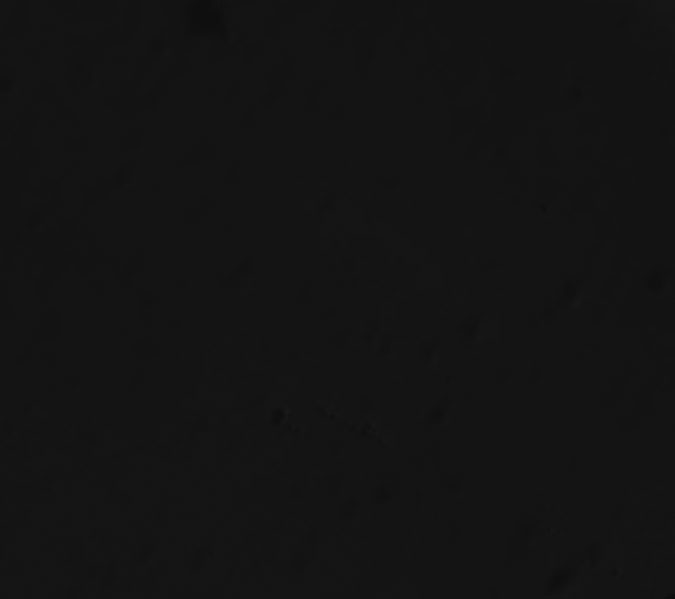

Supplement: Supplementary file 5 — Supplementary Code [file 41467_2023_36045_MOESM5_ESM.zip › Source Code/Untreated raw data for testing the code/PLGA NPs/Image441.jpg]

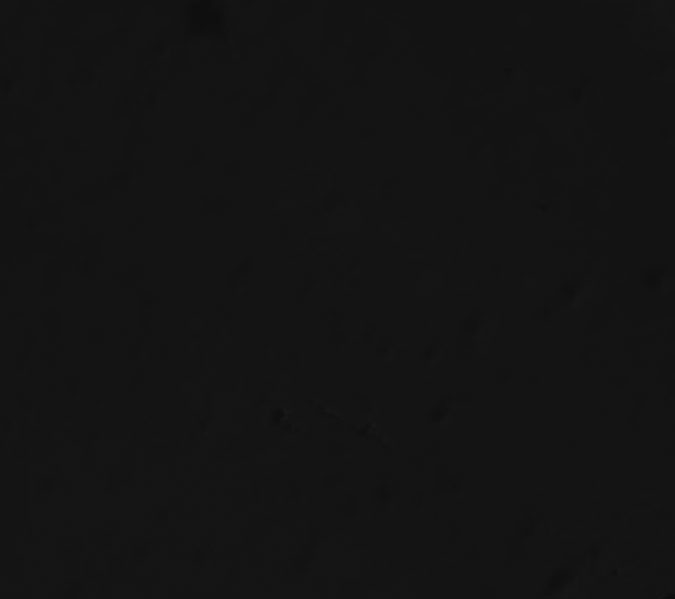

Supplement: Supplementary file 5 — Supplementary Code [file 41467_2023_36045_MOESM5_ESM.zip › Source Code/Untreated raw data for testing the code/PLGA NPs/Image469.jpg]

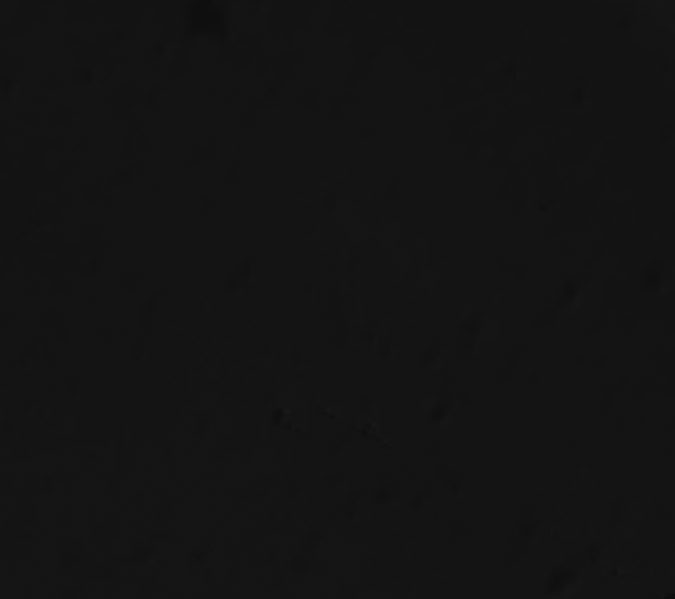

Supplement: Supplementary file 5 — Supplementary Code [file 41467_2023_36045_MOESM5_ESM.zip › Source Code/Untreated raw data for testing the code/PLGA NPs/Image496.jpg]

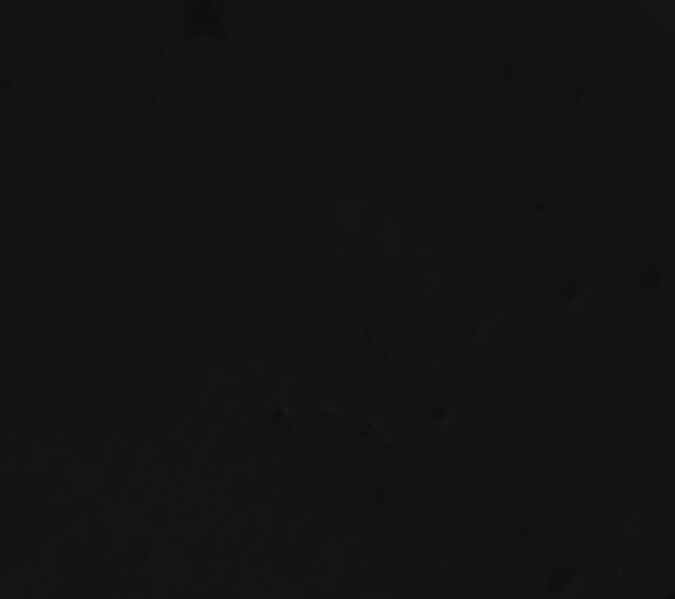

Supplement: Supplementary file 5 — Supplementary Code [file 41467_2023_36045_MOESM5_ESM.zip › Source Code/Untreated raw data for testing the code/PLGA NPs/Image482.jpg]

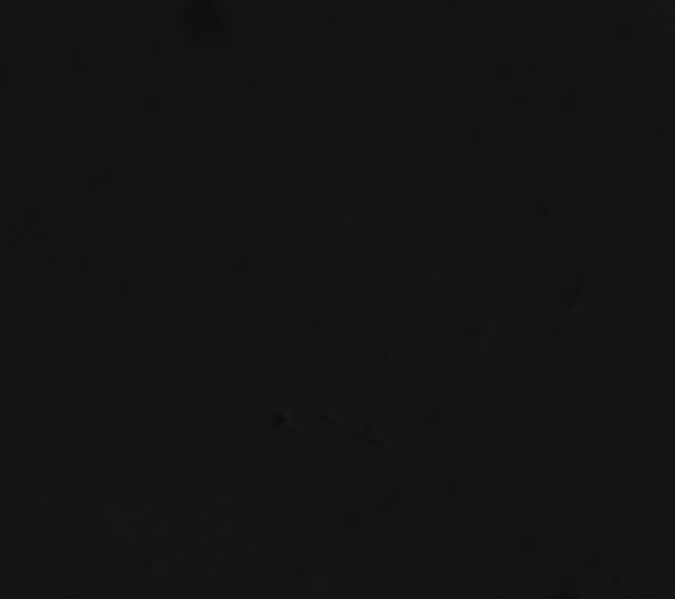

Supplement: Supplementary file 5 — Supplementary Code [file 41467_2023_36045_MOESM5_ESM.zip › Source Code/Untreated raw data for testing the code/PLGA NPs/Image131.jpg]

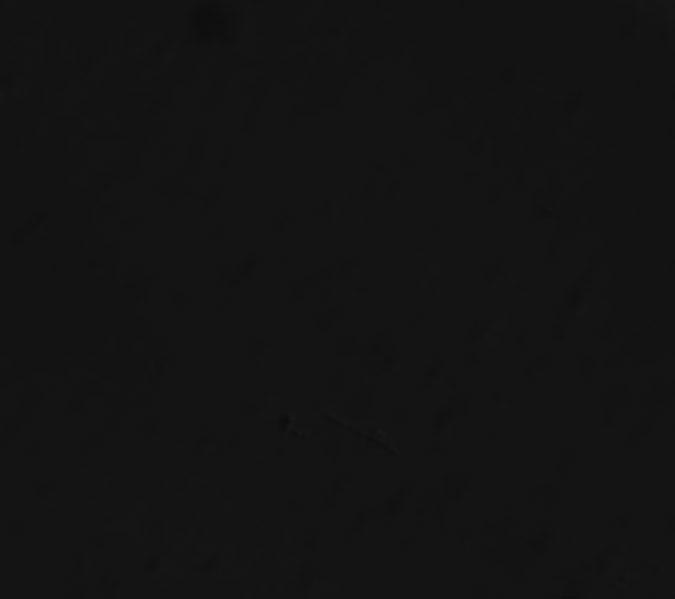

Supplement: Supplementary file 5 — Supplementary Code [file 41467_2023_36045_MOESM5_ESM.zip › Source Code/Untreated raw data for testing the code/PLGA NPs/Image125.jpg]

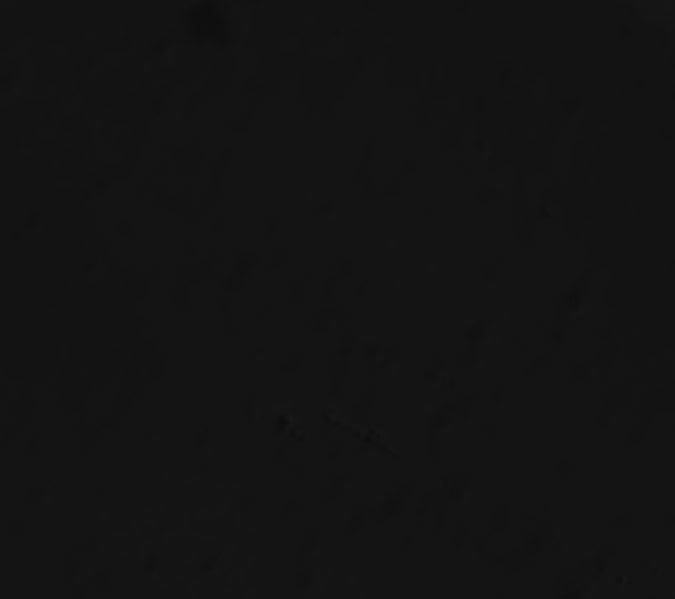

Supplement: Supplementary file 5 — Supplementary Code [file 41467_2023_36045_MOESM5_ESM.zip › Source Code/Untreated raw data for testing the code/PLGA NPs/Image119.jpg]

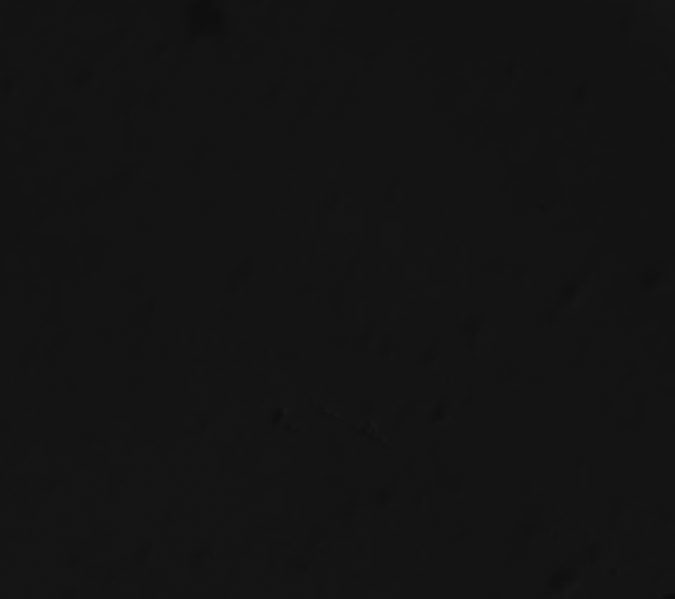

Supplement: Supplementary file 5 — Supplementary Code [file 41467_2023_36045_MOESM5_ESM.zip › Source Code/Untreated raw data for testing the code/PLGA NPs/Image521.jpg]

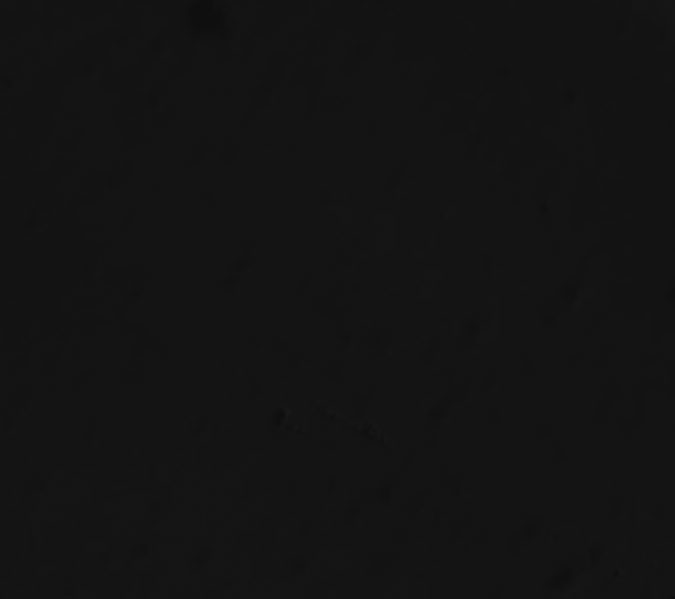

Supplement: Supplementary file 5 — Supplementary Code [file 41467_2023_36045_MOESM5_ESM.zip › Source Code/Untreated raw data for testing the code/PLGA NPs/Image247.jpg]

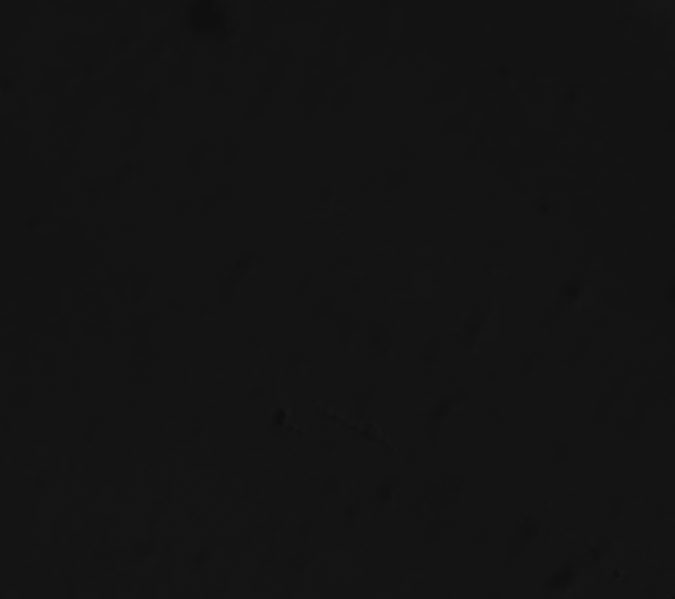

Supplement: Supplementary file 5 — Supplementary Code [file 41467_2023_36045_MOESM5_ESM.zip › Source Code/Untreated raw data for testing the code/PLGA NPs/Image253.jpg]

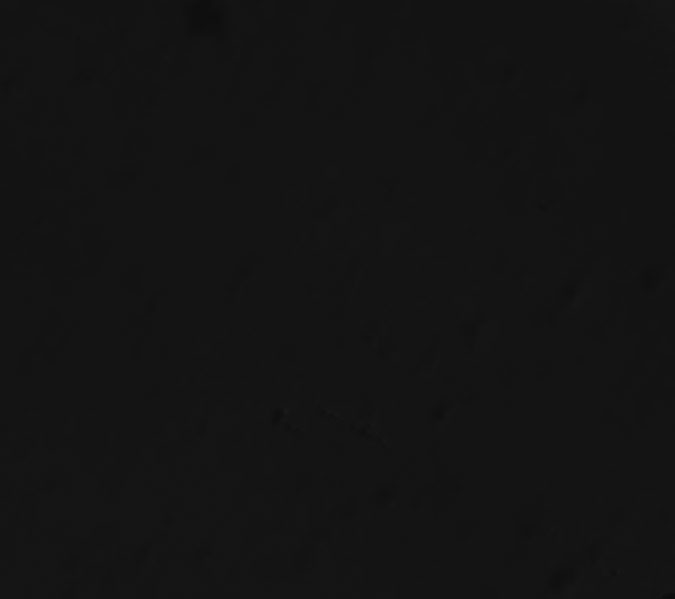

Supplement: Supplementary file 5 — Supplementary Code [file 41467_2023_36045_MOESM5_ESM.zip › Source Code/Untreated raw data for testing the code/PLGA NPs/Image535.jpg]

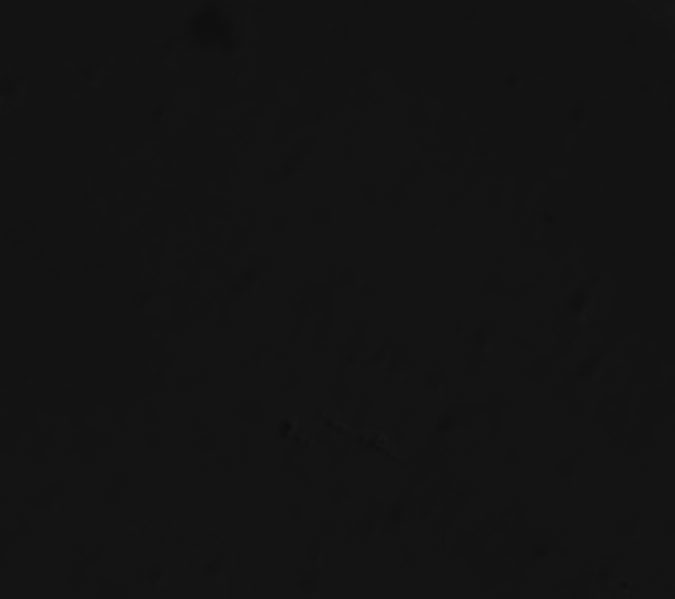

Supplement: Supplementary file 5 — Supplementary Code [file 41467_2023_36045_MOESM5_ESM.zip › Source Code/Untreated raw data for testing the code/PLGA NPs/Image66.jpg]

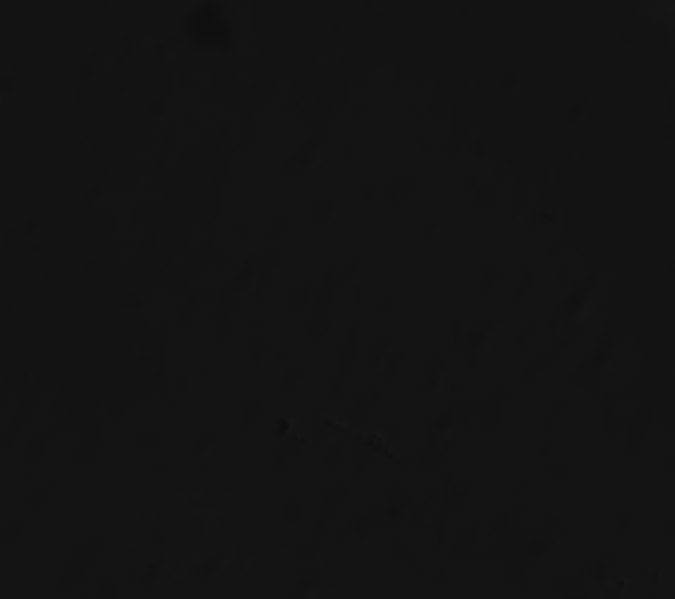

Supplement: Supplementary file 5 — Supplementary Code [file 41467_2023_36045_MOESM5_ESM.zip › Source Code/Untreated raw data for testing the code/PLGA NPs/Image72.jpg]

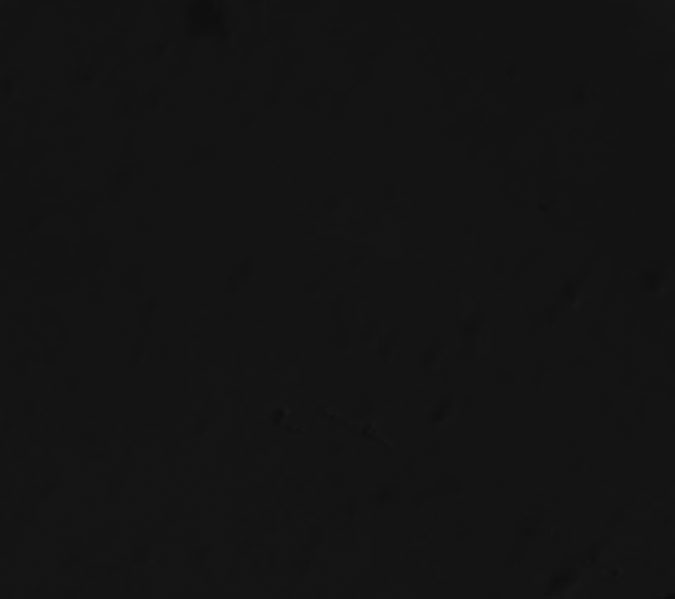

Supplement: Supplementary file 5 — Supplementary Code [file 41467_2023_36045_MOESM5_ESM.zip › Source Code/Untreated raw data for testing the code/PLGA NPs/Image509.jpg]

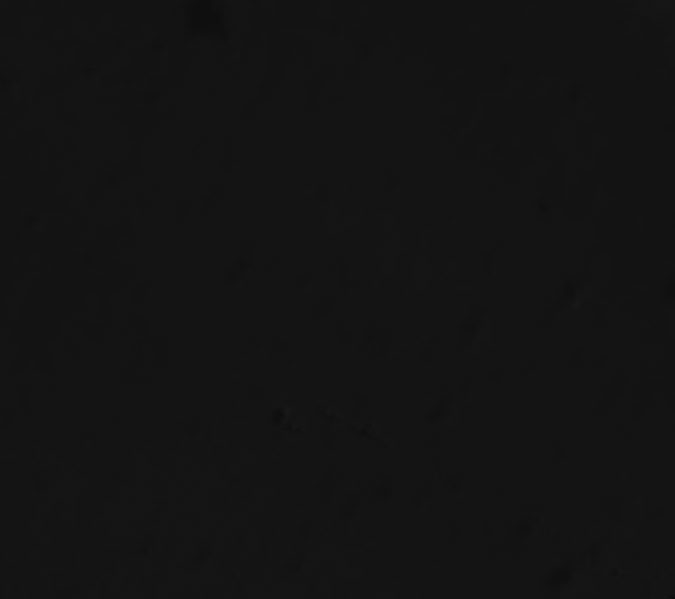

Supplement: Supplementary file 5 — Supplementary Code [file 41467_2023_36045_MOESM5_ESM.zip › Source Code/Untreated raw data for testing the code/PLGA NPs/Image284.jpg]

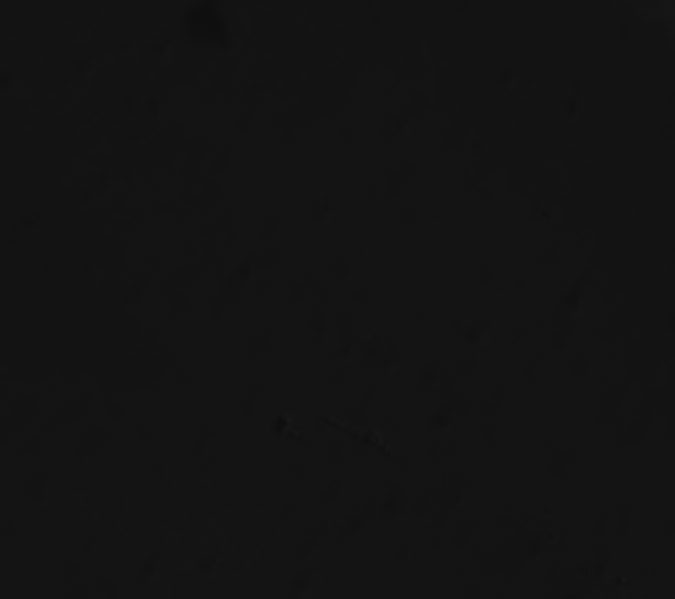

Supplement: Supplementary file 5 — Supplementary Code [file 41467_2023_36045_MOESM5_ESM.zip › Source Code/Untreated raw data for testing the code/PLGA NPs/Image99.jpg]

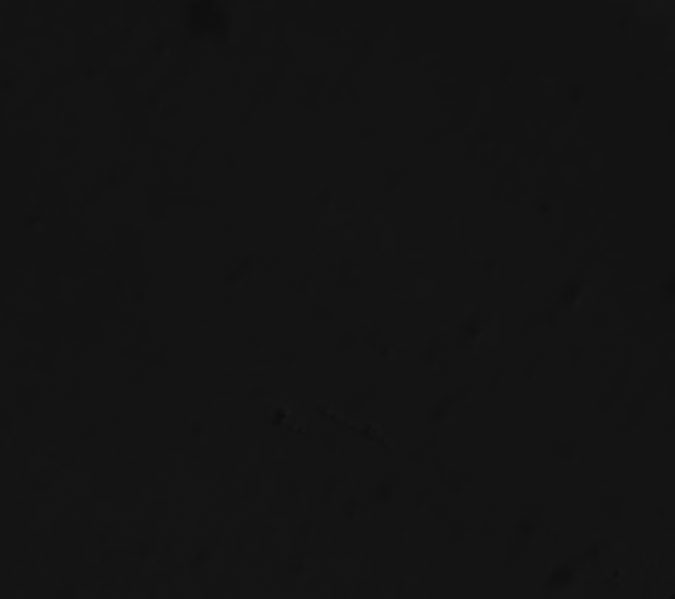

Supplement: Supplementary file 5 — Supplementary Code [file 41467_2023_36045_MOESM5_ESM.zip › Source Code/Untreated raw data for testing the code/PLGA NPs/Image290.jpg]

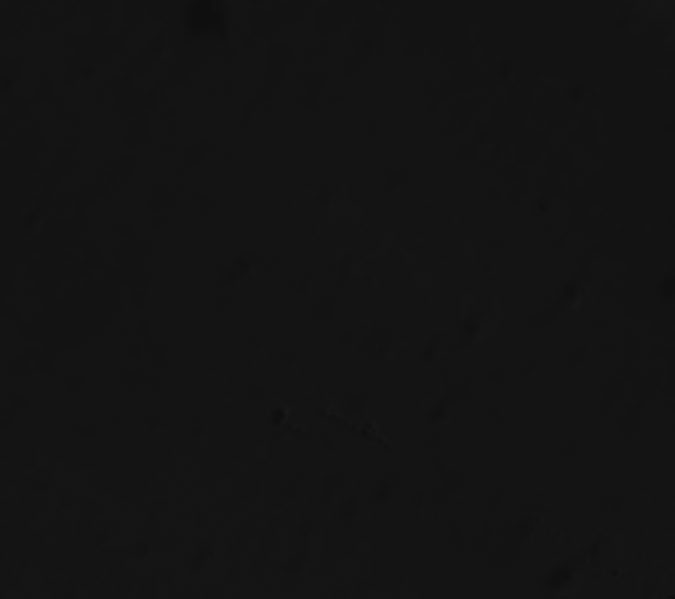

Supplement: Supplementary file 5 — Supplementary Code [file 41467_2023_36045_MOESM5_ESM.zip › Source Code/Untreated raw data for testing the code/PLGA NPs/Image291.jpg]

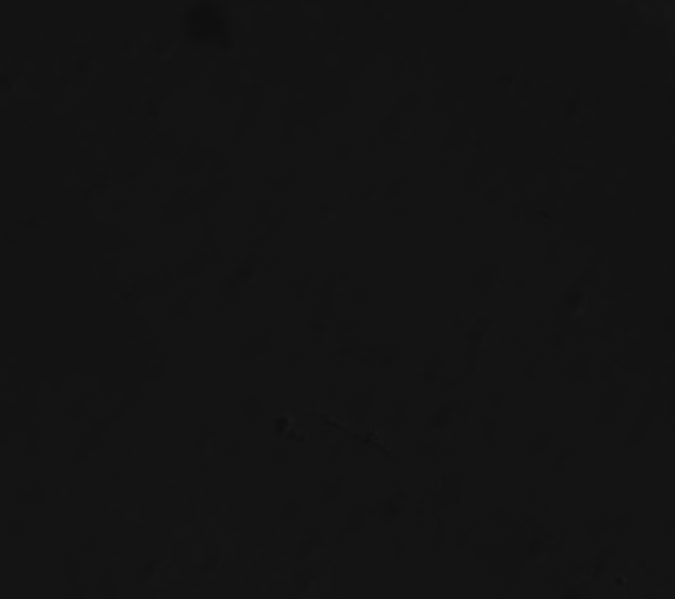

Supplement: Supplementary file 5 — Supplementary Code [file 41467_2023_36045_MOESM5_ESM.zip › Source Code/Untreated raw data for testing the code/PLGA NPs/Image98.jpg]

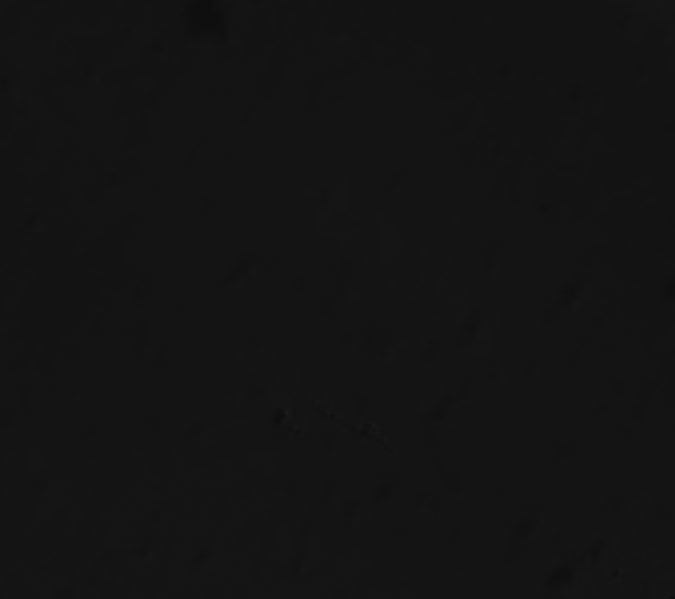

Supplement: Supplementary file 5 — Supplementary Code [file 41467_2023_36045_MOESM5_ESM.zip › Source Code/Untreated raw data for testing the code/PLGA NPs/Image285.jpg]

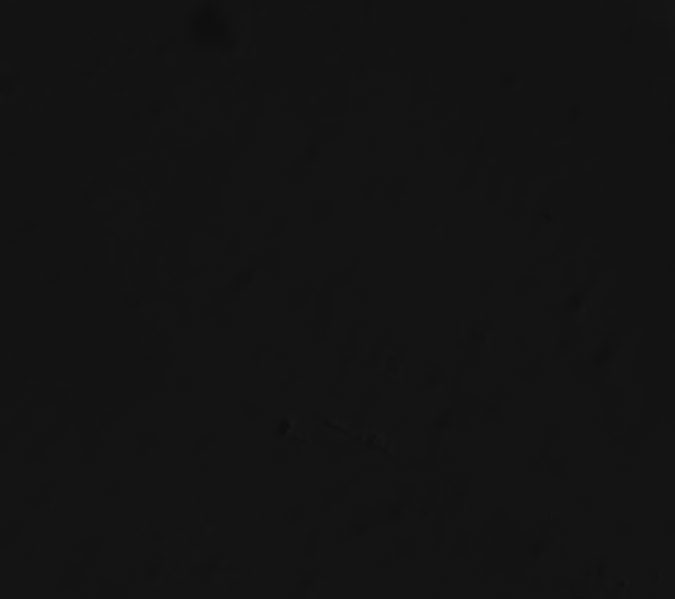

Supplement: Supplementary file 5 — Supplementary Code [file 41467_2023_36045_MOESM5_ESM.zip › Source Code/Untreated raw data for testing the code/PLGA NPs/Image73.jpg]

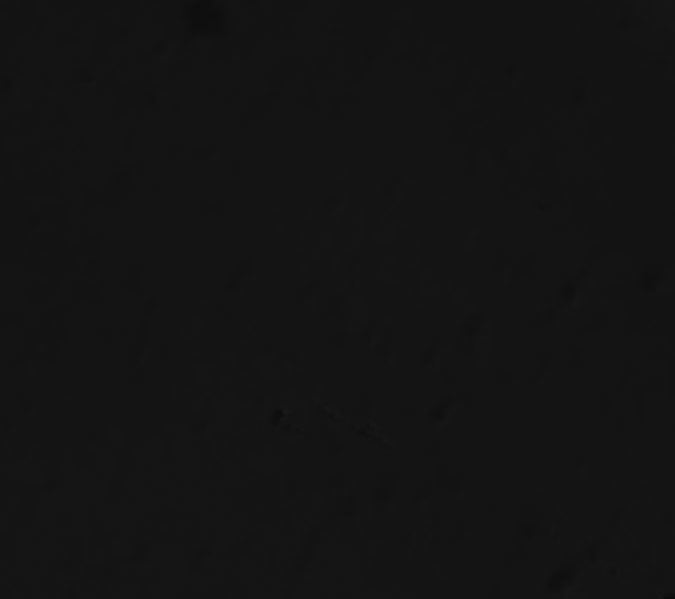

Supplement: Supplementary file 5 — Supplementary Code [file 41467_2023_36045_MOESM5_ESM.zip › Source Code/Untreated raw data for testing the code/PLGA NPs/Image508.jpg]

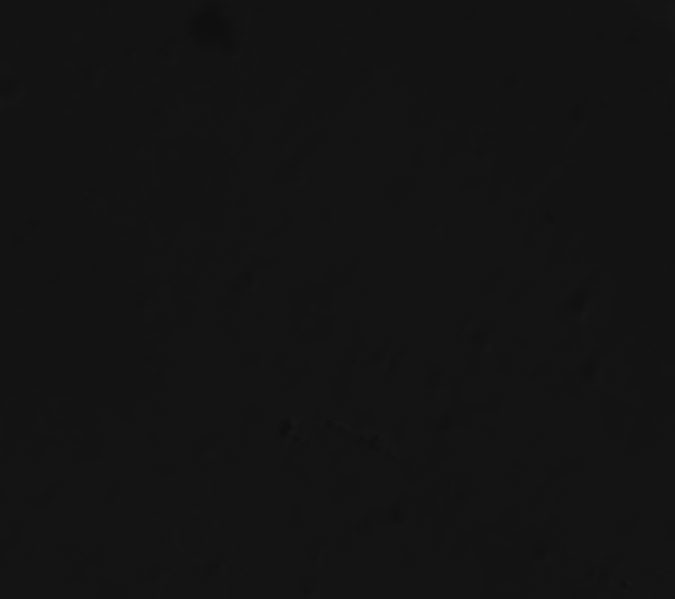

Supplement: Supplementary file 5 — Supplementary Code [file 41467_2023_36045_MOESM5_ESM.zip › Source Code/Untreated raw data for testing the code/PLGA NPs/Image67.jpg]

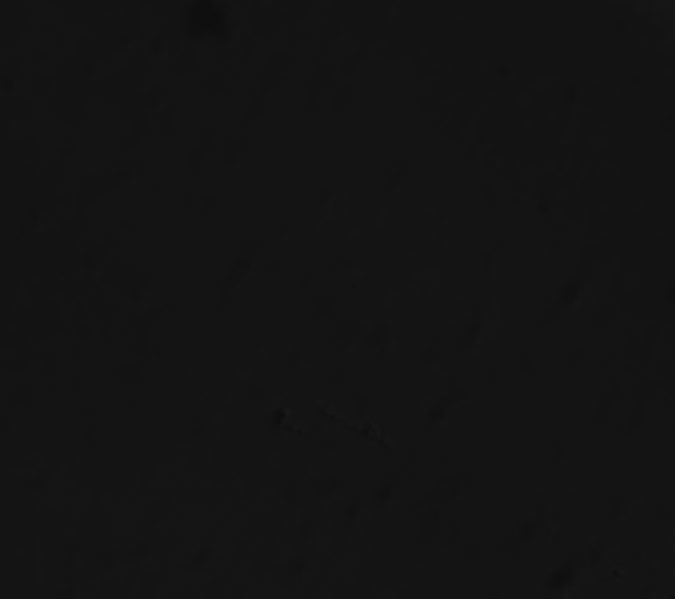

Supplement: Supplementary file 5 — Supplementary Code [file 41467_2023_36045_MOESM5_ESM.zip › Source Code/Untreated raw data for testing the code/PLGA NPs/Image252.jpg]

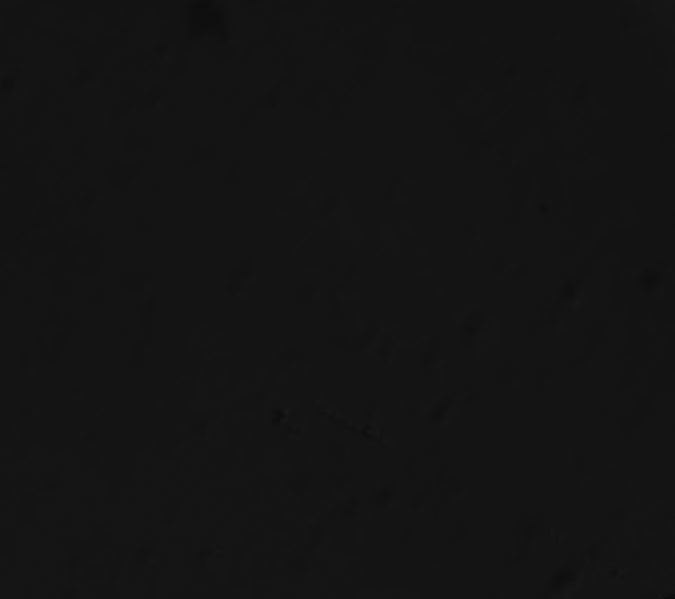

Supplement: Supplementary file 5 — Supplementary Code [file 41467_2023_36045_MOESM5_ESM.zip › Source Code/Untreated raw data for testing the code/PLGA NPs/Image534.jpg]

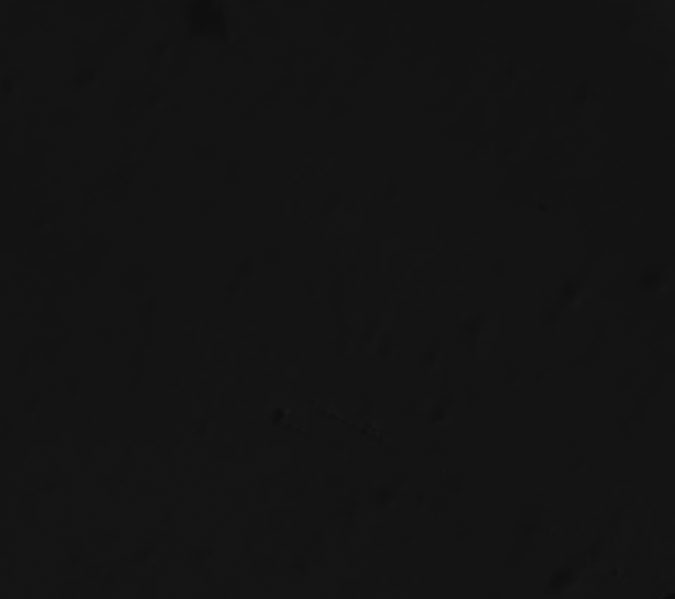

Supplement: Supplementary file 5 — Supplementary Code [file 41467_2023_36045_MOESM5_ESM.zip › Source Code/Untreated raw data for testing the code/PLGA NPs/Image520.jpg]

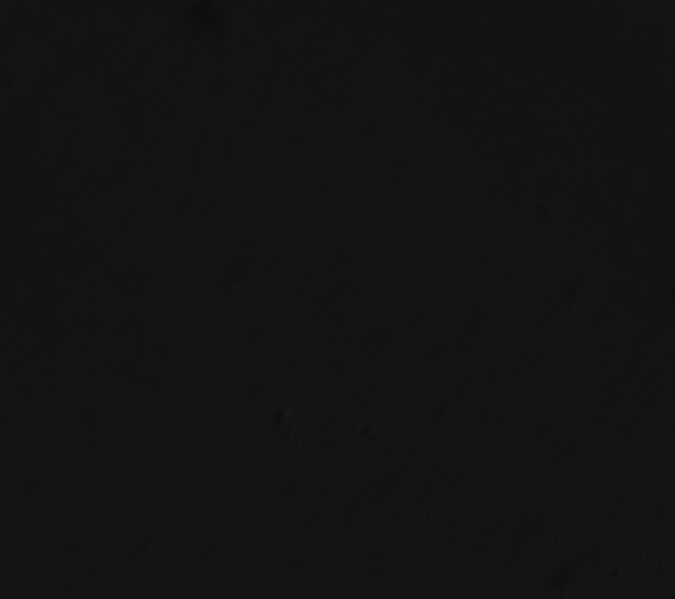

Supplement: Supplementary file 5 — Supplementary Code [file 41467_2023_36045_MOESM5_ESM.zip › Source Code/Untreated raw data for testing the code/PLGA NPs/Image246.jpg]

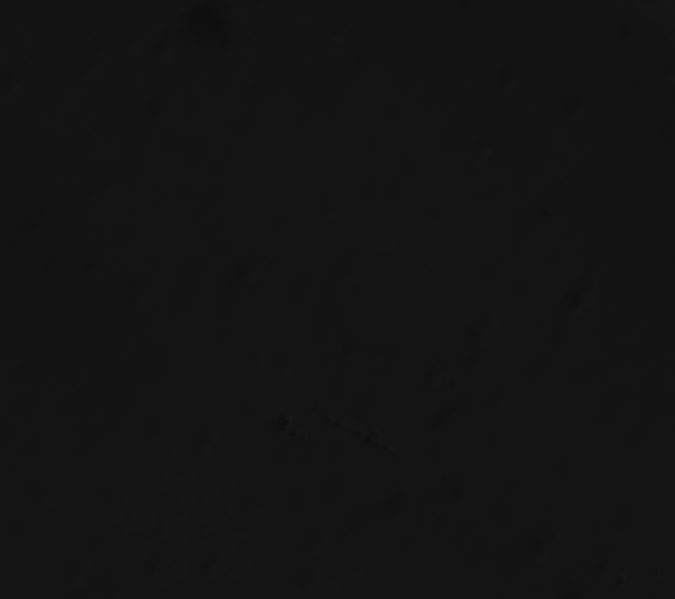

Supplement: Supplementary file 5 — Supplementary Code [file 41467_2023_36045_MOESM5_ESM.zip › Source Code/Untreated raw data for testing the code/PLGA NPs/Image118.jpg]

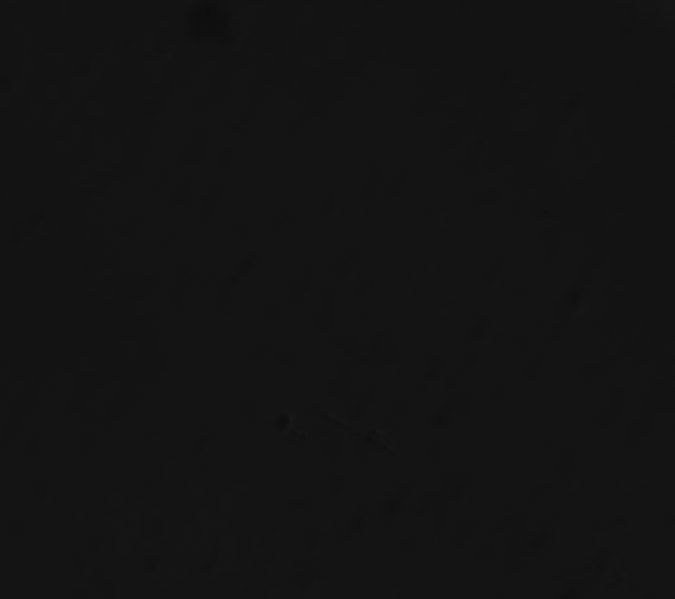

Supplement: Supplementary file 5 — Supplementary Code [file 41467_2023_36045_MOESM5_ESM.zip › Source Code/Untreated raw data for testing the code/PLGA NPs/Image124.jpg]

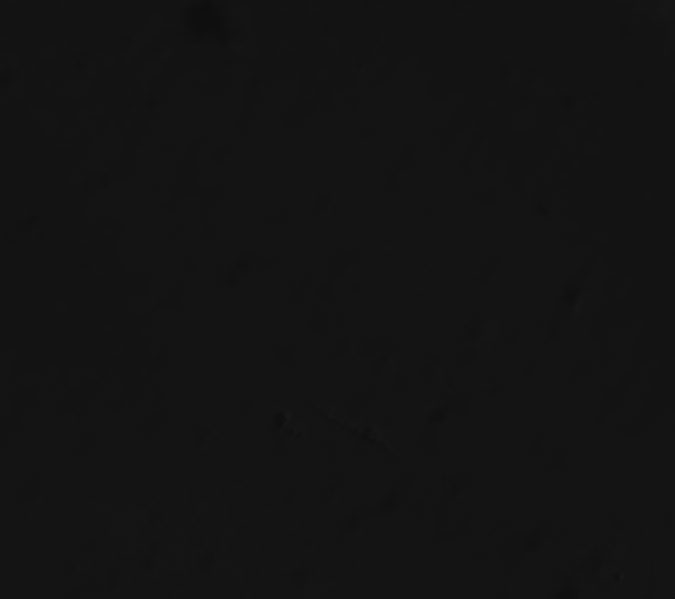

Supplement: Supplementary file 5 — Supplementary Code [file 41467_2023_36045_MOESM5_ESM.zip › Source Code/Untreated raw data for testing the code/PLGA NPs/Image130.jpg]

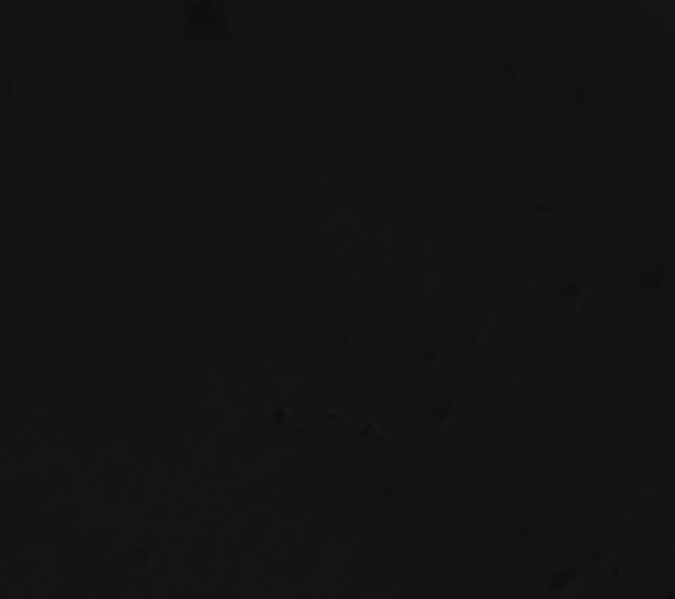

Supplement: Supplementary file 5 — Supplementary Code [file 41467_2023_36045_MOESM5_ESM.zip › Source Code/Untreated raw data for testing the code/PLGA NPs/Image483.jpg]

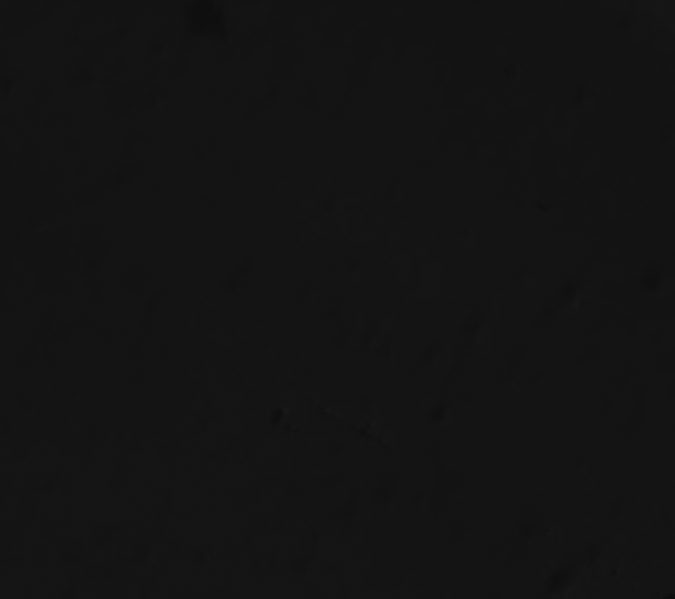

Supplement: Supplementary file 5 — Supplementary Code [file 41467_2023_36045_MOESM5_ESM.zip › Source Code/Untreated raw data for testing the code/PLGA NPs/Image497.jpg]

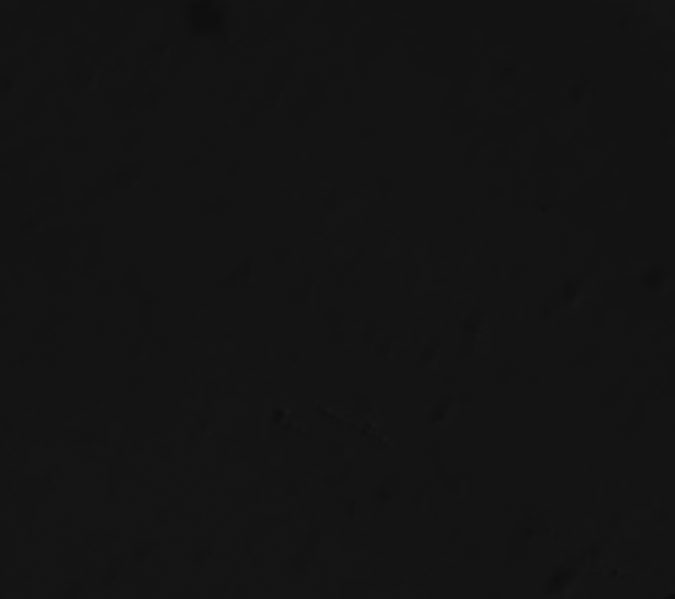

Supplement: Supplementary file 5 — Supplementary Code [file 41467_2023_36045_MOESM5_ESM.zip › Source Code/Untreated raw data for testing the code/PLGA NPs/Image468.jpg]

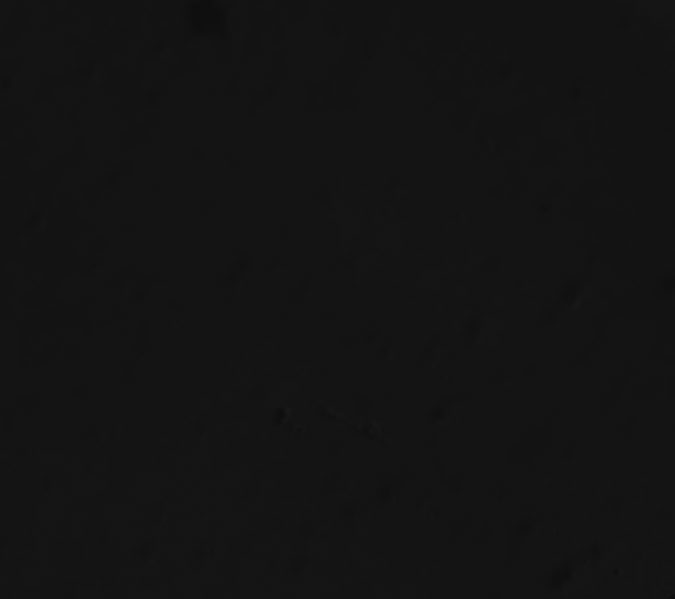

Supplement: Supplementary file 5 — Supplementary Code [file 41467_2023_36045_MOESM5_ESM.zip › Source Code/Untreated raw data for testing the code/PLGA NPs/Image326.jpg]

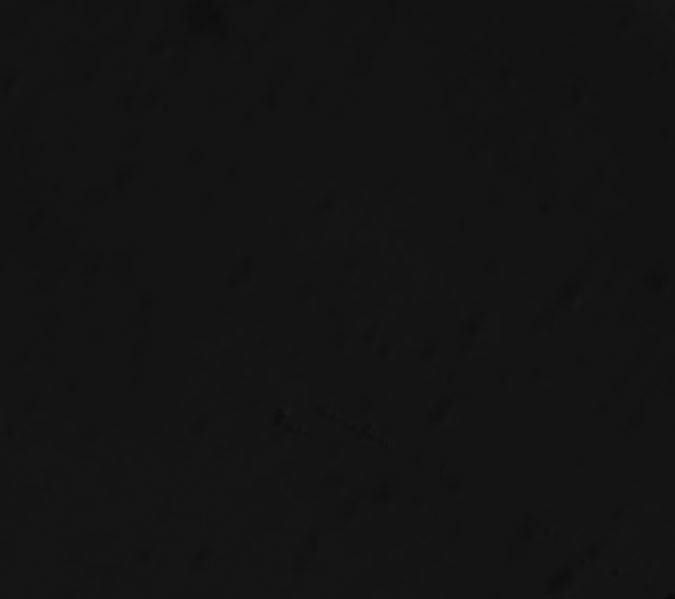

Supplement: Supplementary file 5 — Supplementary Code [file 41467_2023_36045_MOESM5_ESM.zip › Source Code/Untreated raw data for testing the code/PLGA NPs/Image440.jpg]

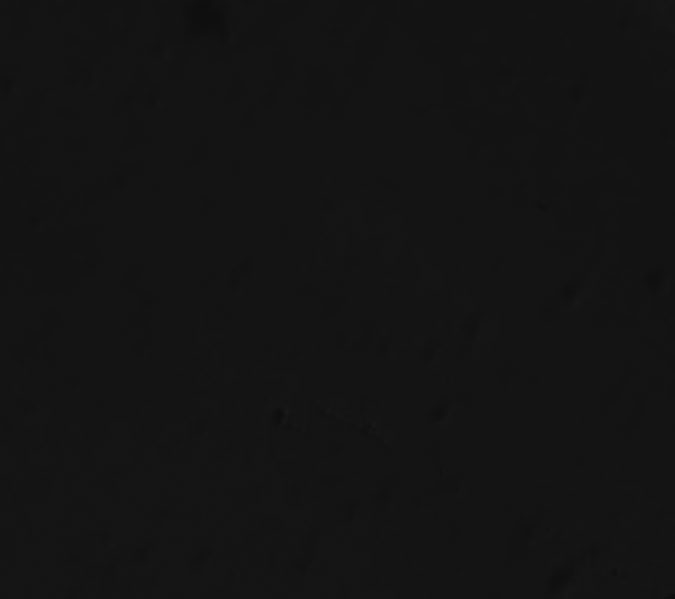

Supplement: Supplementary file 5 — Supplementary Code [file 41467_2023_36045_MOESM5_ESM.zip › Source Code/Untreated raw data for testing the code/PLGA NPs/Image454.jpg]

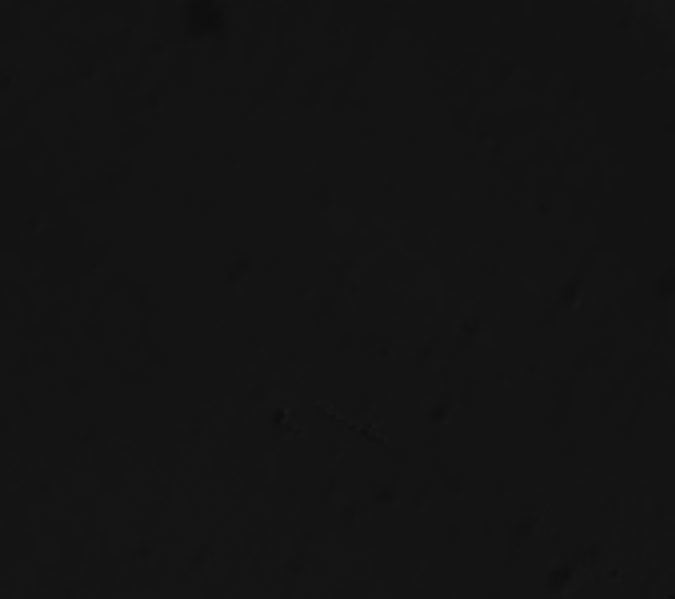

Supplement: Supplementary file 5 — Supplementary Code [file 41467_2023_36045_MOESM5_ESM.zip › Source Code/Untreated raw data for testing the code/PLGA NPs/Image332.jpg]

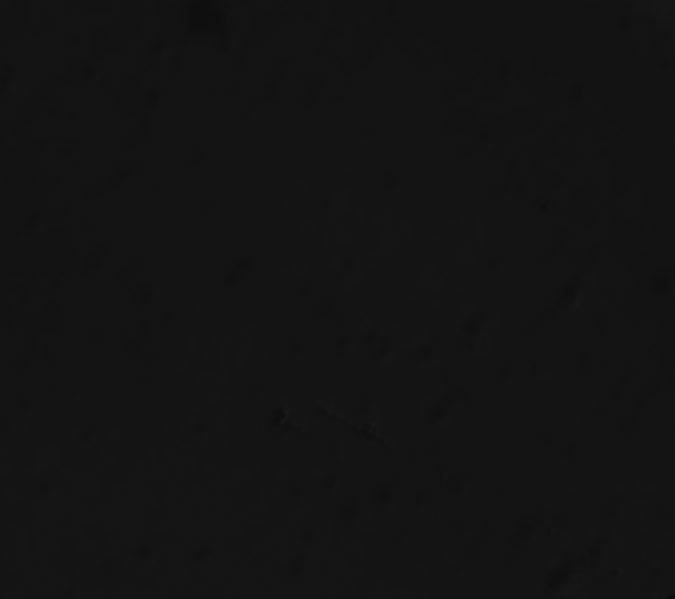

Supplement: Supplementary file 5 — Supplementary Code [file 41467_2023_36045_MOESM5_ESM.zip › Source Code/Untreated raw data for testing the code/PLGA NPs/Image369.jpg]

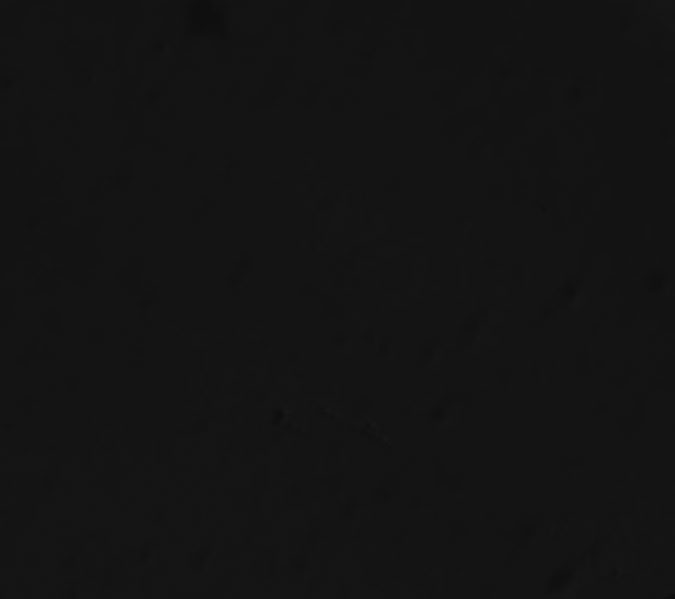

Supplement: Supplementary file 5 — Supplementary Code [file 41467_2023_36045_MOESM5_ESM.zip › Source Code/Untreated raw data for testing the code/PLGA NPs/Image433.jpg]

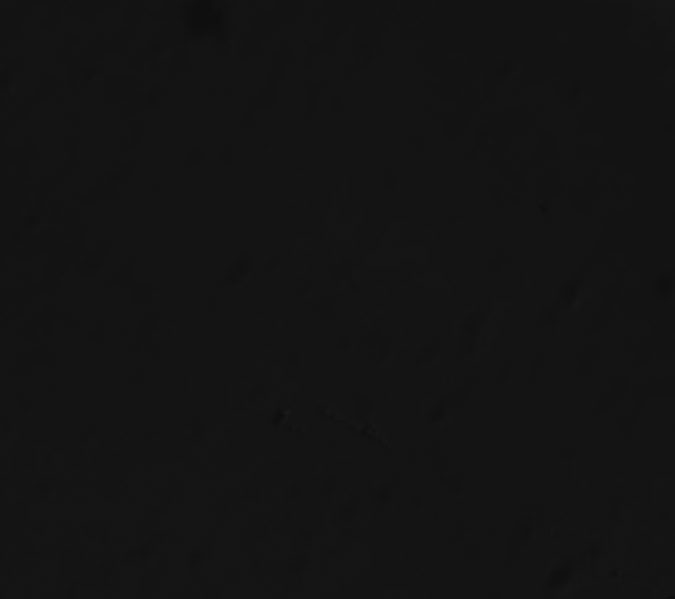

Supplement: Supplementary file 5 — Supplementary Code [file 41467_2023_36045_MOESM5_ESM.zip › Source Code/Untreated raw data for testing the code/PLGA NPs/Image355.jpg]

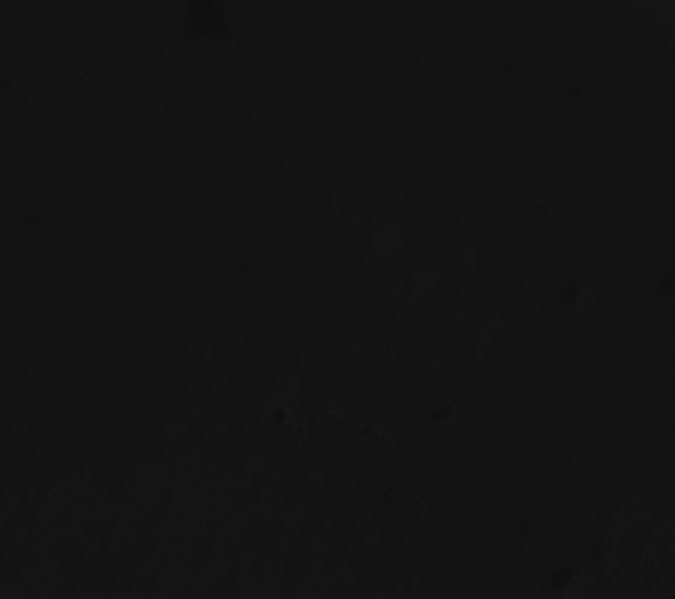

Supplement: Supplementary file 5 — Supplementary Code [file 41467_2023_36045_MOESM5_ESM.zip › Source Code/Untreated raw data for testing the code/PLGA NPs/Image341.jpg]

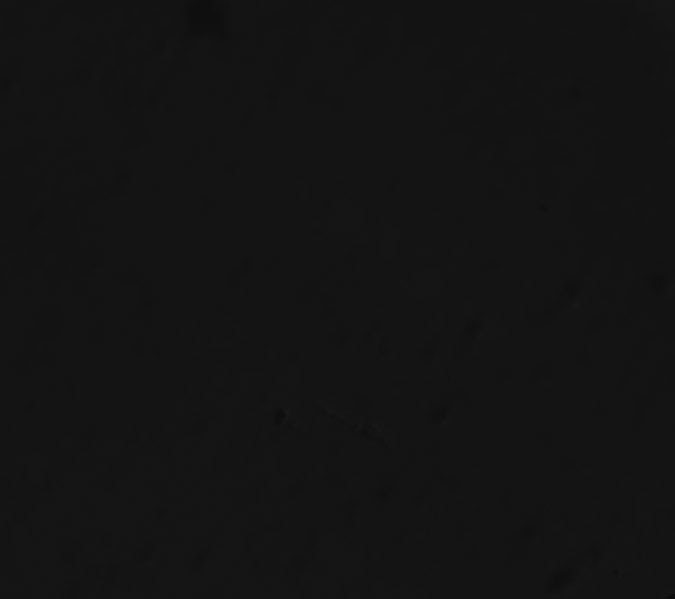

Supplement: Supplementary file 5 — Supplementary Code [file 41467_2023_36045_MOESM5_ESM.zip › Source Code/Untreated raw data for testing the code/PLGA NPs/Image427.jpg]

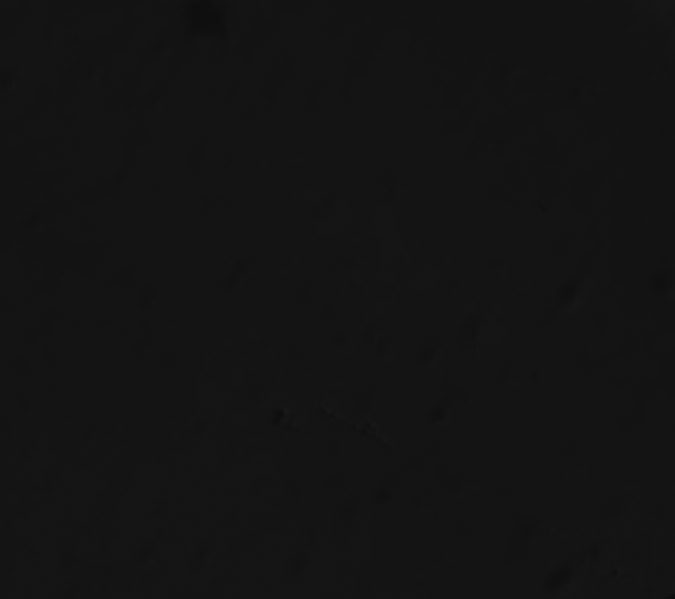

Supplement: Supplementary file 5 — Supplementary Code [file 41467_2023_36045_MOESM5_ESM.zip › Source Code/Untreated raw data for testing the code/PLGA NPs/Image396.jpg]

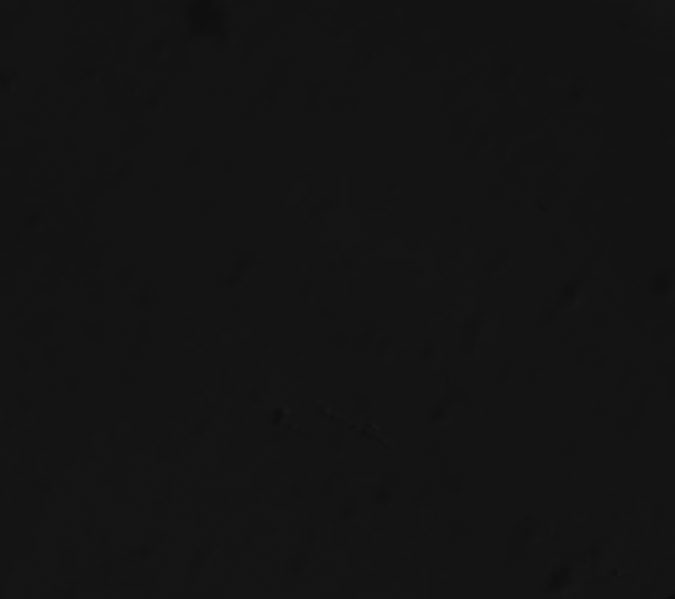

Supplement: Supplementary file 5 — Supplementary Code [file 41467_2023_36045_MOESM5_ESM.zip › Source Code/Untreated raw data for testing the code/PLGA NPs/Image382.jpg]

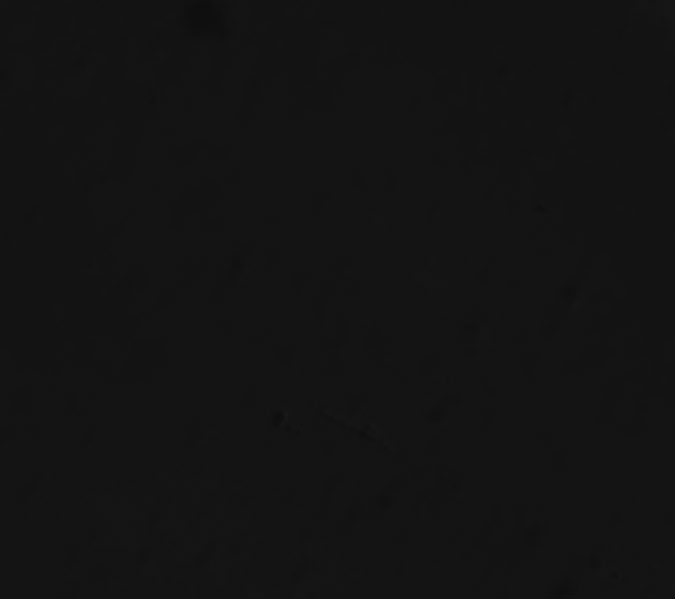

Supplement: Supplementary file 5 — Supplementary Code [file 41467_2023_36045_MOESM5_ESM.zip › Source Code/Untreated raw data for testing the code/PLGA NPs/Image157.jpg]

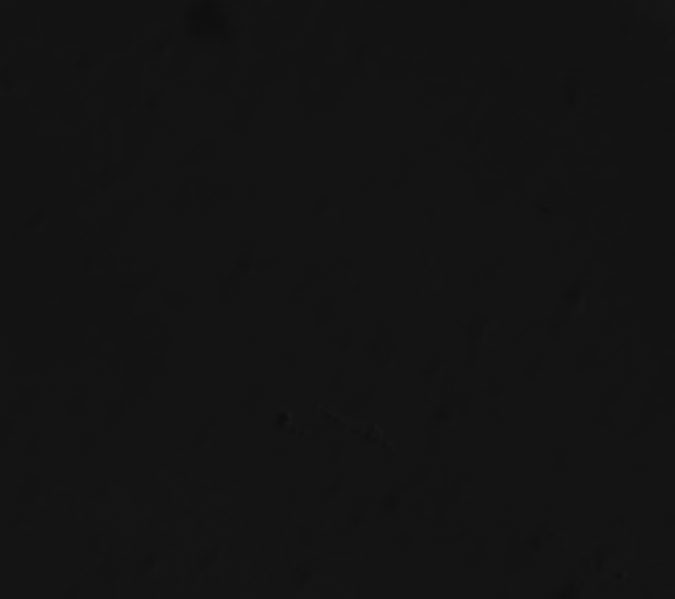

Supplement: Supplementary file 5 — Supplementary Code [file 41467_2023_36045_MOESM5_ESM.zip › Source Code/Untreated raw data for testing the code/PLGA NPs/Image143.jpg]

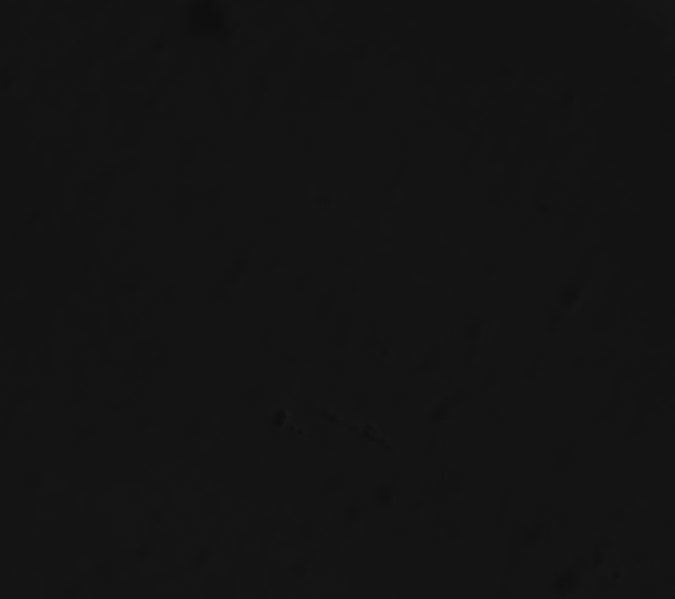

Supplement: Supplementary file 5 — Supplementary Code [file 41467_2023_36045_MOESM5_ESM.zip › Source Code/Untreated raw data for testing the code/PLGA NPs/Image194.jpg]

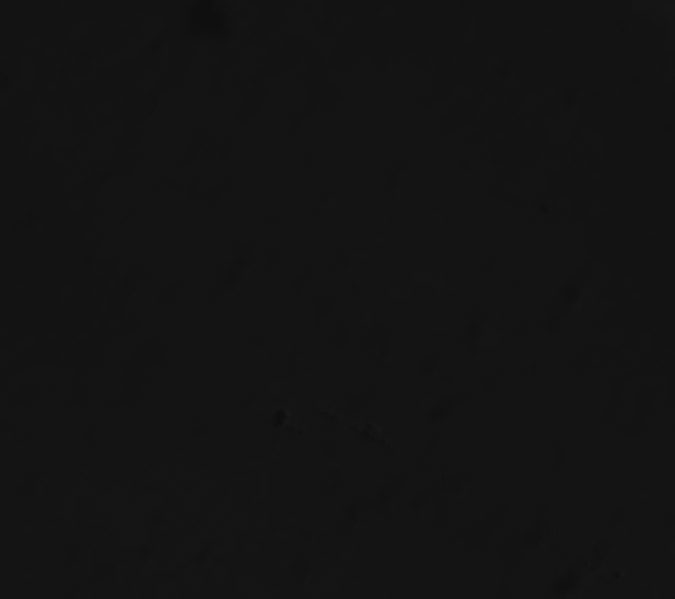

Supplement: Supplementary file 5 — Supplementary Code [file 41467_2023_36045_MOESM5_ESM.zip › Source Code/Untreated raw data for testing the code/PLGA NPs/Image180.jpg]

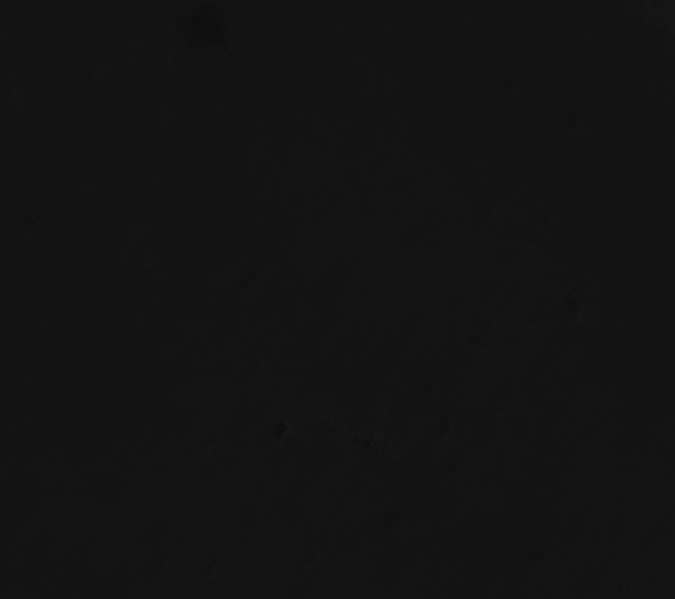

Supplement: Supplementary file 5 — Supplementary Code [file 41467_2023_36045_MOESM5_ESM.zip › Source Code/Untreated raw data for testing the code/PLGA NPs/Image14.jpg]

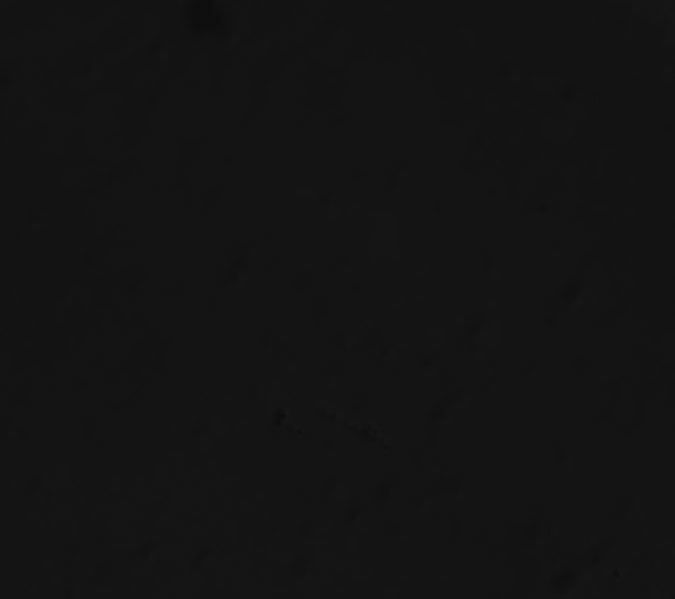

Supplement: Supplementary file 5 — Supplementary Code [file 41467_2023_36045_MOESM5_ESM.zip › Source Code/Untreated raw data for testing the code/PLGA NPs/Image209.jpg]

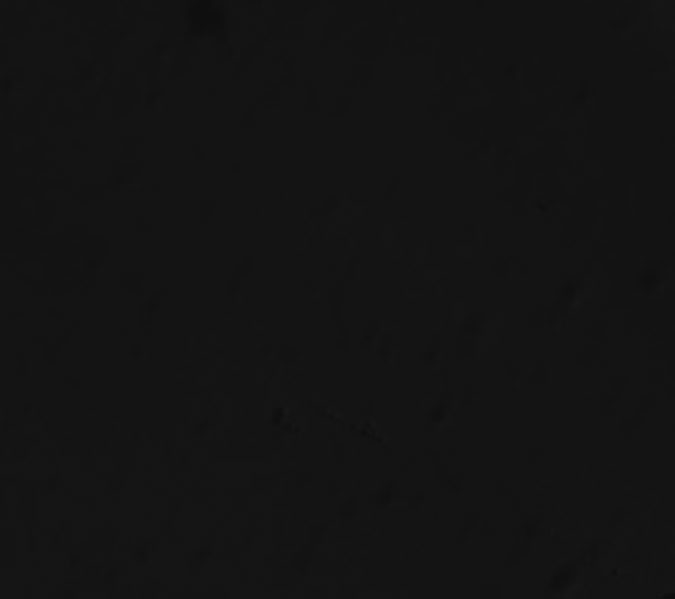

Supplement: Supplementary file 5 — Supplementary Code [file 41467_2023_36045_MOESM5_ESM.zip › Source Code/Untreated raw data for testing the code/PLGA NPs/Image547.jpg]

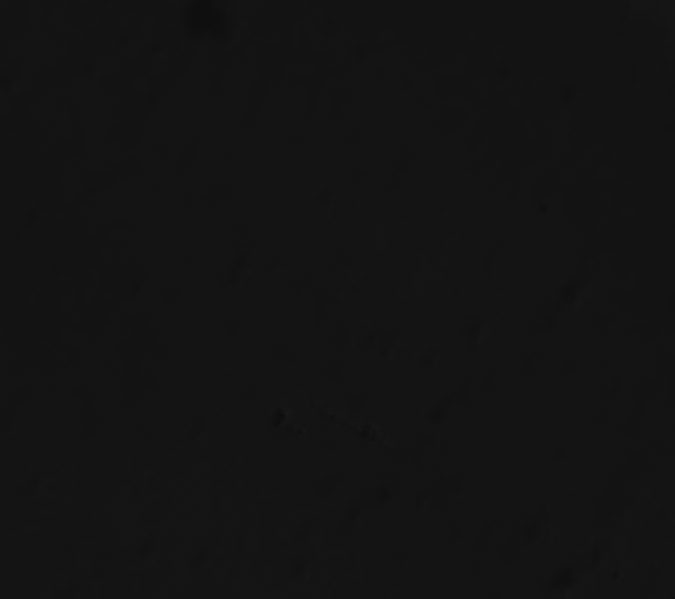

Supplement: Supplementary file 5 — Supplementary Code [file 41467_2023_36045_MOESM5_ESM.zip › Source Code/Untreated raw data for testing the code/PLGA NPs/Image221.jpg]

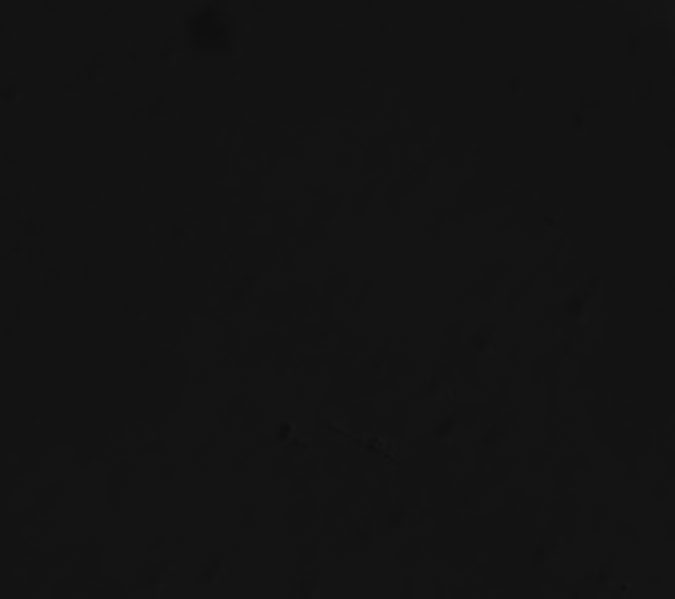

Supplement: Supplementary file 5 — Supplementary Code [file 41467_2023_36045_MOESM5_ESM.zip › Source Code/Untreated raw data for testing the code/PLGA NPs/Image28.jpg]

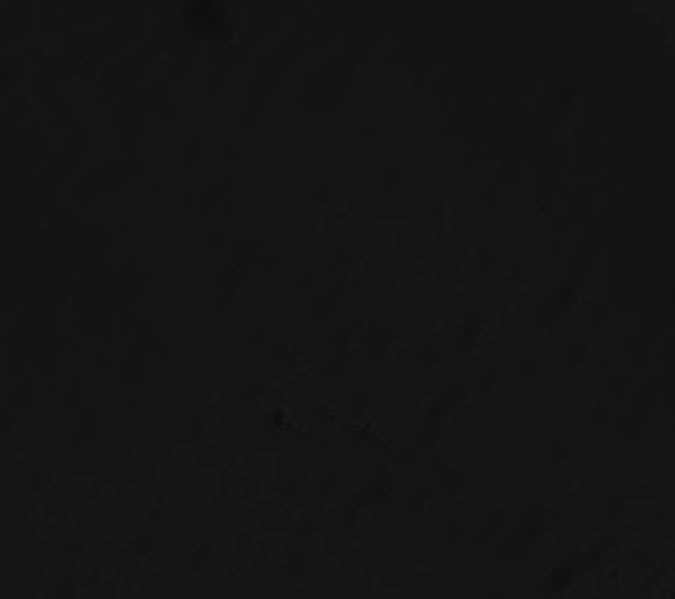

Supplement: Supplementary file 5 — Supplementary Code [file 41467_2023_36045_MOESM5_ESM.zip › Source Code/Untreated raw data for testing the code/PLGA NPs/Image235.jpg]

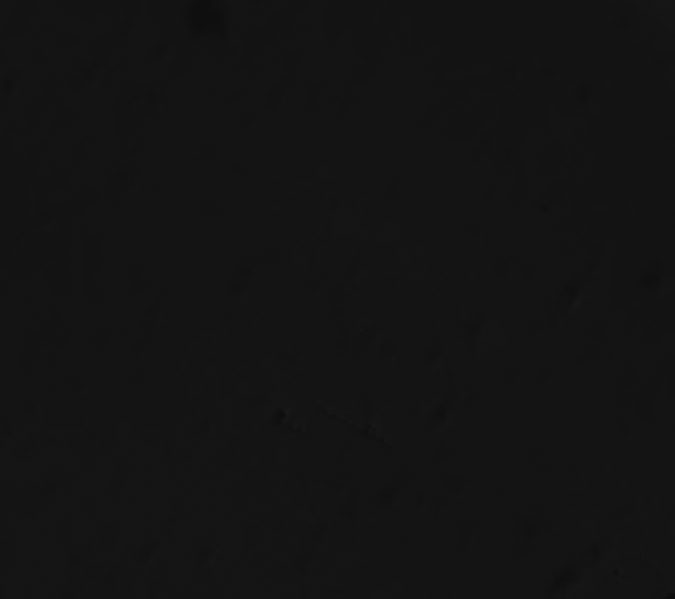

Supplement: Supplementary file 5 — Supplementary Code [file 41467_2023_36045_MOESM5_ESM.zip › Source Code/Untreated raw data for testing the code/PLGA NPs/Image553.jpg]

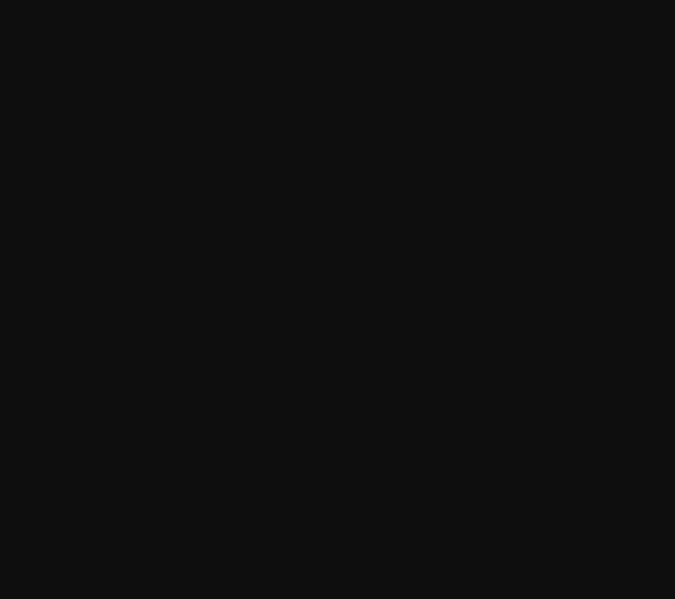

Supplement: Supplementary file 5 — Supplementary Code [file 41467_2023_36045_MOESM5_ESM.zip › Source Code/Untreated raw data for testing the code/PLGA NPs/Image584.jpg]

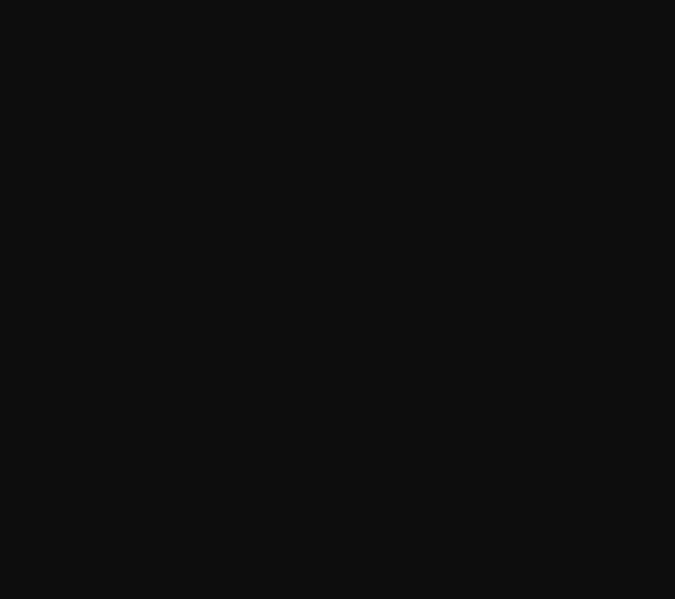

Supplement: Supplementary file 5 — Supplementary Code [file 41467_2023_36045_MOESM5_ESM.zip › Source Code/Untreated raw data for testing the code/PLGA NPs/Image590.jpg]

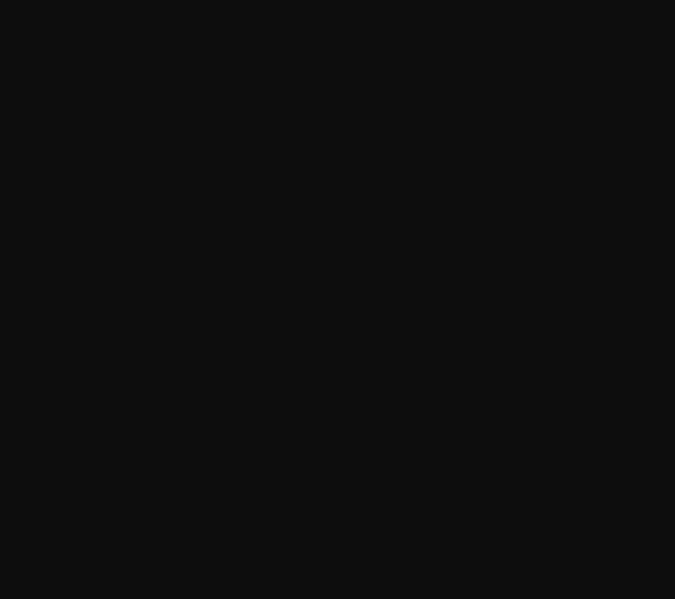

Supplement: Supplementary file 5 — Supplementary Code [file 41467_2023_36045_MOESM5_ESM.zip › Source Code/Untreated raw data for testing the code/PLGA NPs/Image591.jpg]

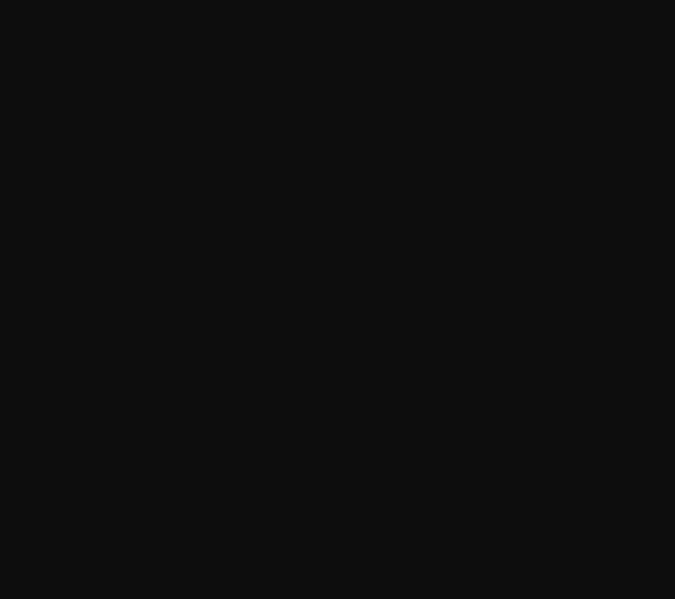

Supplement: Supplementary file 5 — Supplementary Code [file 41467_2023_36045_MOESM5_ESM.zip › Source Code/Untreated raw data for testing the code/PLGA NPs/Image585.jpg]

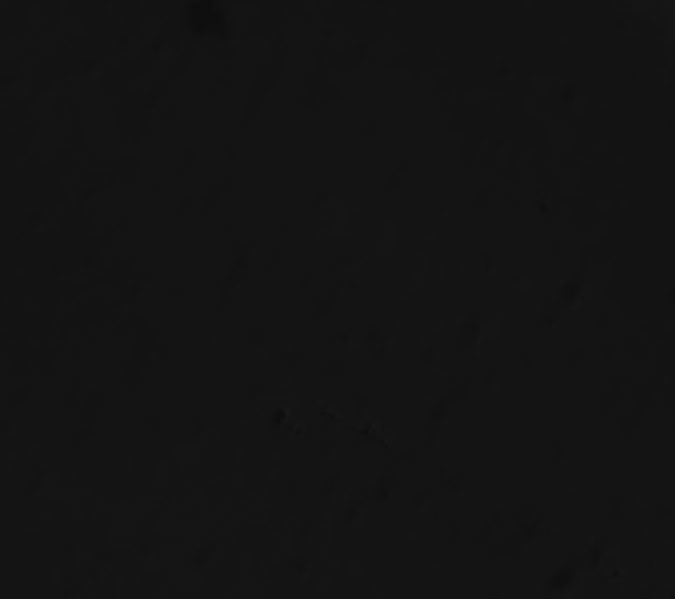

Supplement: Supplementary file 5 — Supplementary Code [file 41467_2023_36045_MOESM5_ESM.zip › Source Code/Untreated raw data for testing the code/PLGA NPs/Image234.jpg]

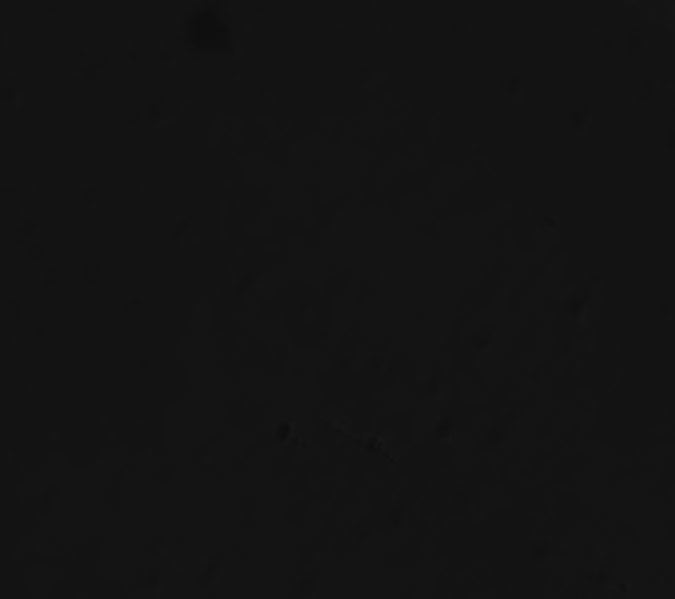

Supplement: Supplementary file 5 — Supplementary Code [file 41467_2023_36045_MOESM5_ESM.zip › Source Code/Untreated raw data for testing the code/PLGA NPs/Image29.jpg]

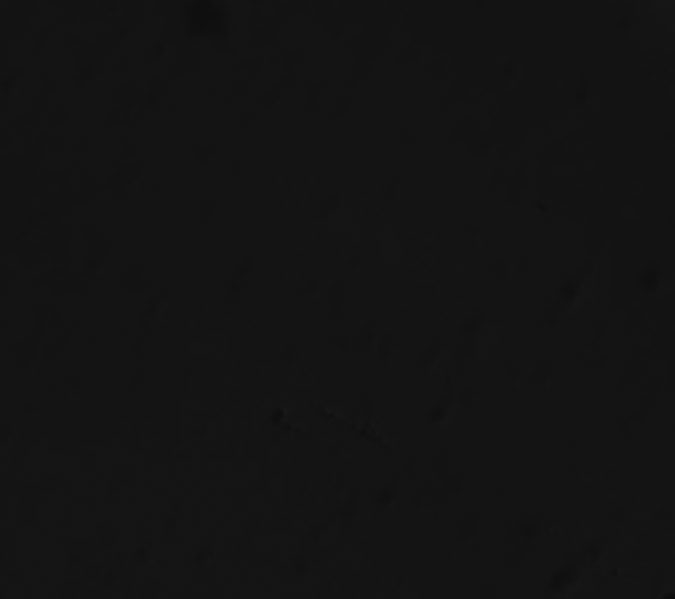

Supplement: Supplementary file 5 — Supplementary Code [file 41467_2023_36045_MOESM5_ESM.zip › Source Code/Untreated raw data for testing the code/PLGA NPs/Image552.jpg]

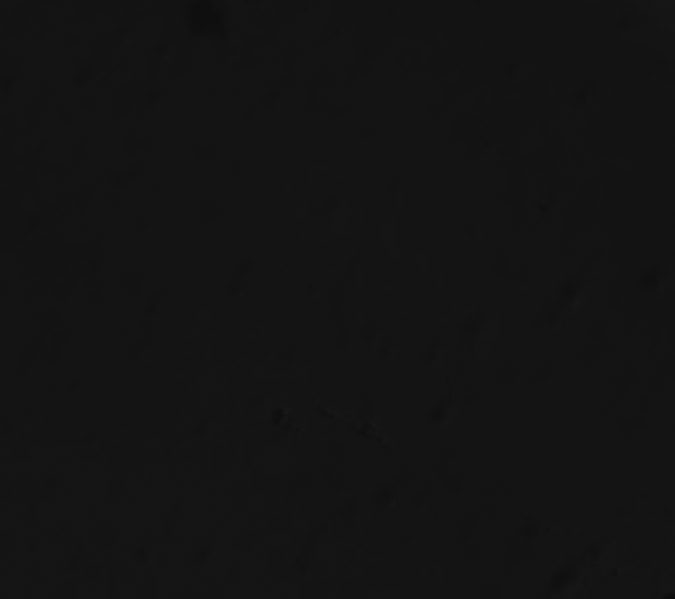

Supplement: Supplementary file 5 — Supplementary Code [file 41467_2023_36045_MOESM5_ESM.zip › Source Code/Untreated raw data for testing the code/PLGA NPs/Image546.jpg]

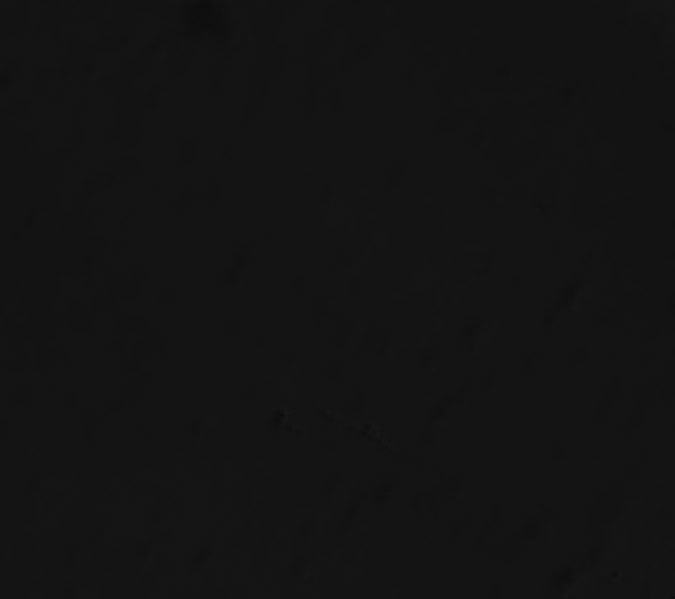

Supplement: Supplementary file 5 — Supplementary Code [file 41467_2023_36045_MOESM5_ESM.zip › Source Code/Untreated raw data for testing the code/PLGA NPs/Image220.jpg]

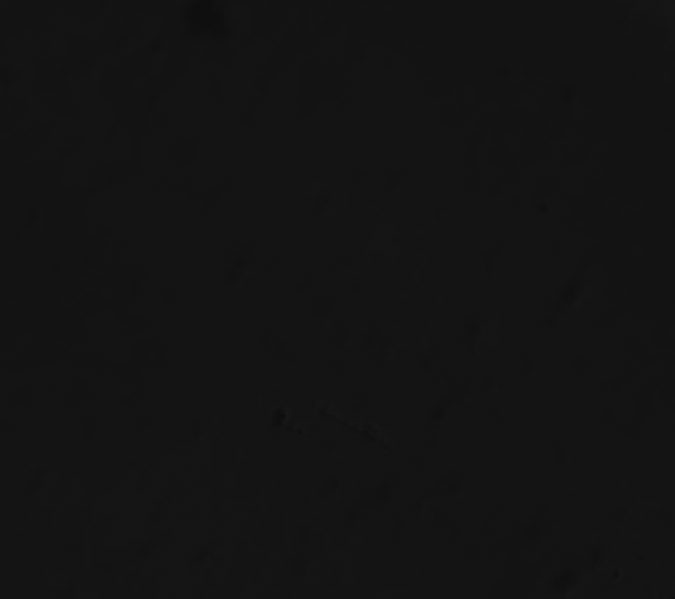

Supplement: Supplementary file 5 — Supplementary Code [file 41467_2023_36045_MOESM5_ESM.zip › Source Code/Untreated raw data for testing the code/PLGA NPs/Image208.jpg]

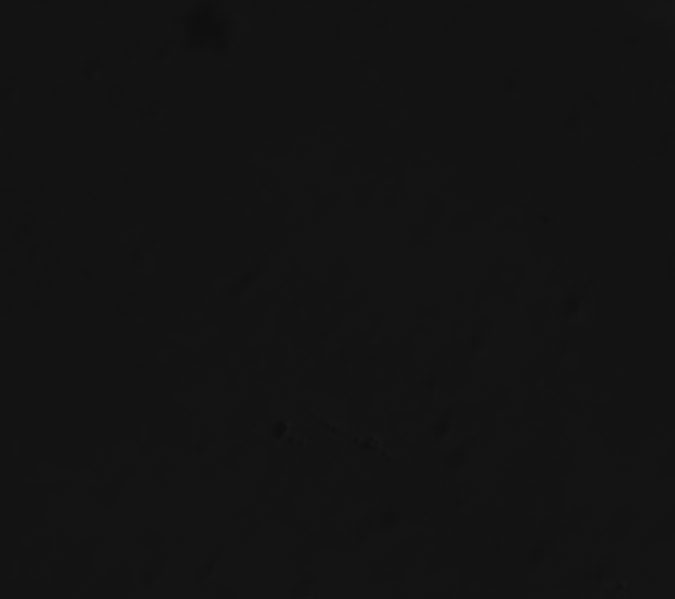

Supplement: Supplementary file 5 — Supplementary Code [file 41467_2023_36045_MOESM5_ESM.zip › Source Code/Untreated raw data for testing the code/PLGA NPs/Image15.jpg]

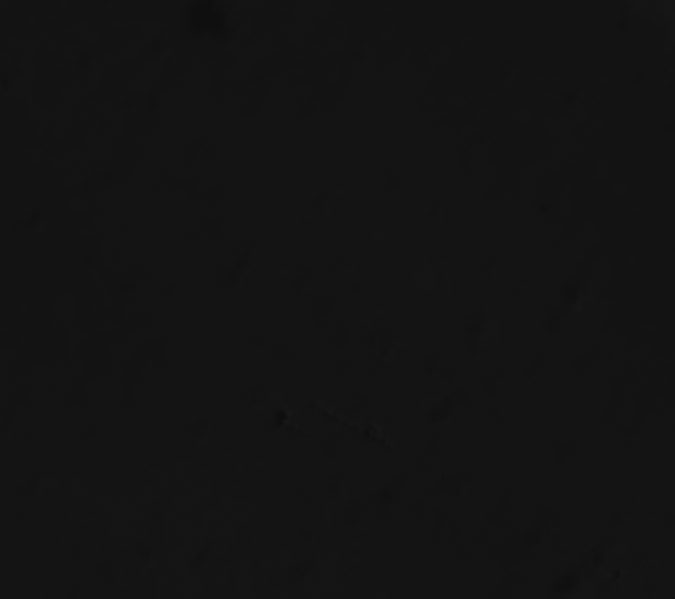

Supplement: Supplementary file 5 — Supplementary Code [file 41467_2023_36045_MOESM5_ESM.zip › Source Code/Untreated raw data for testing the code/PLGA NPs/Image181.jpg]

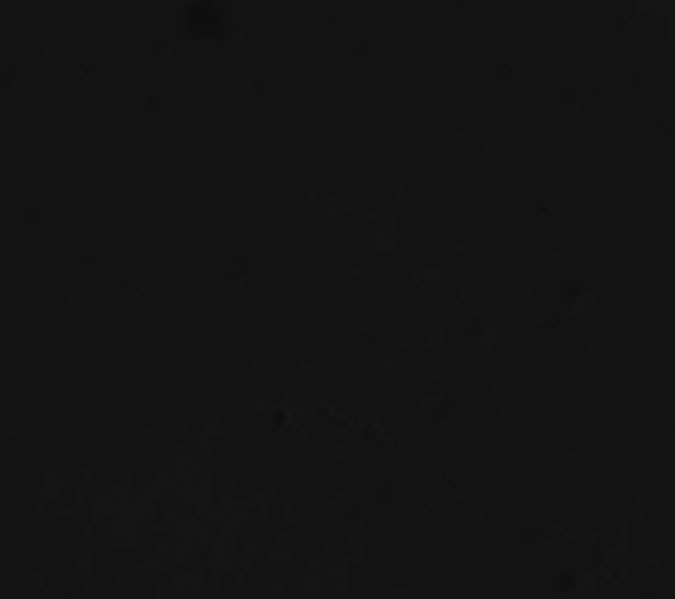

Supplement: Supplementary file 5 — Supplementary Code [file 41467_2023_36045_MOESM5_ESM.zip › Source Code/Untreated raw data for testing the code/PLGA NPs/Image195.jpg]

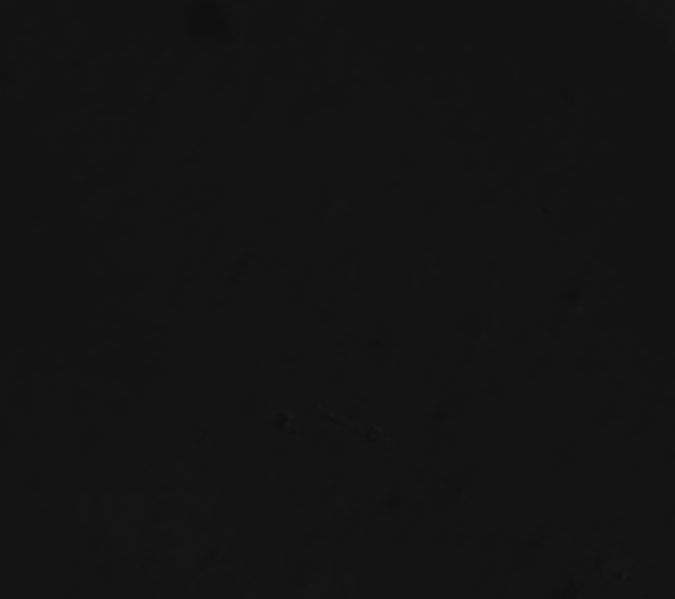

Supplement: Supplementary file 5 — Supplementary Code [file 41467_2023_36045_MOESM5_ESM.zip › Source Code/Untreated raw data for testing the code/PLGA NPs/Image142.jpg]

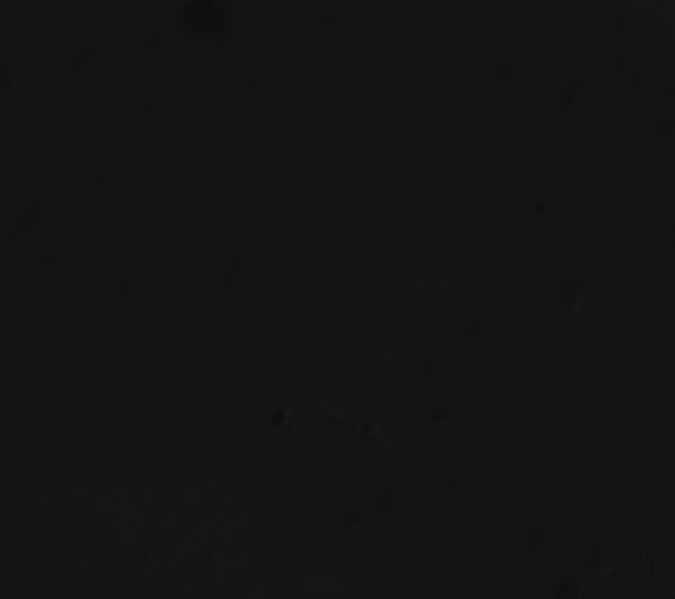

Supplement: Supplementary file 5 — Supplementary Code [file 41467_2023_36045_MOESM5_ESM.zip › Source Code/Untreated raw data for testing the code/PLGA NPs/Image156.jpg]

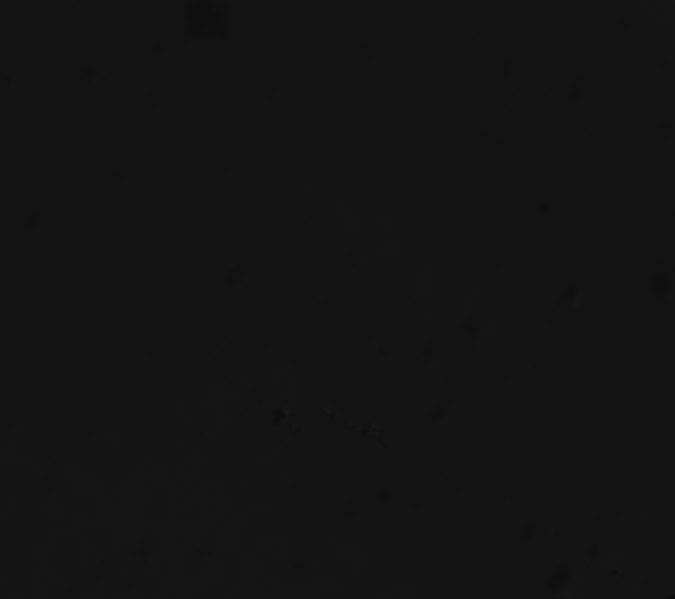

Supplement: Supplementary file 5 — Supplementary Code [file 41467_2023_36045_MOESM5_ESM.zip › Source Code/Untreated raw data for testing the code/PLGA NPs/Image383.jpg]

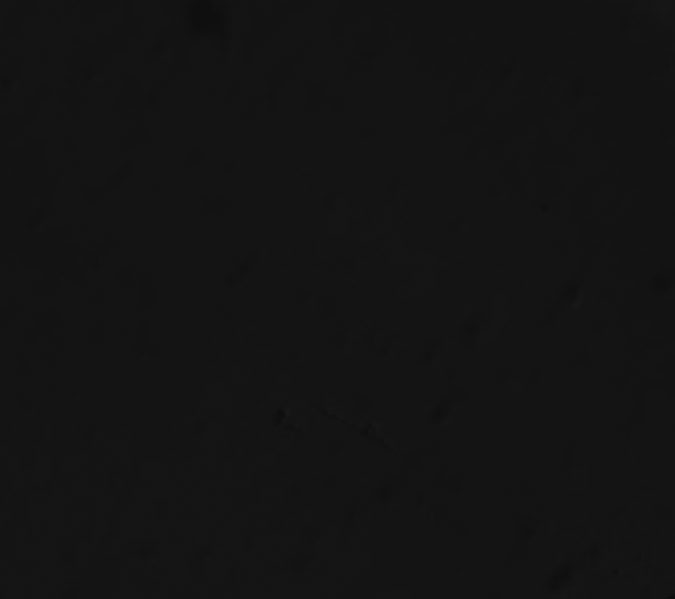

Supplement: Supplementary file 5 — Supplementary Code [file 41467_2023_36045_MOESM5_ESM.zip › Source Code/Untreated raw data for testing the code/PLGA NPs/Image397.jpg]

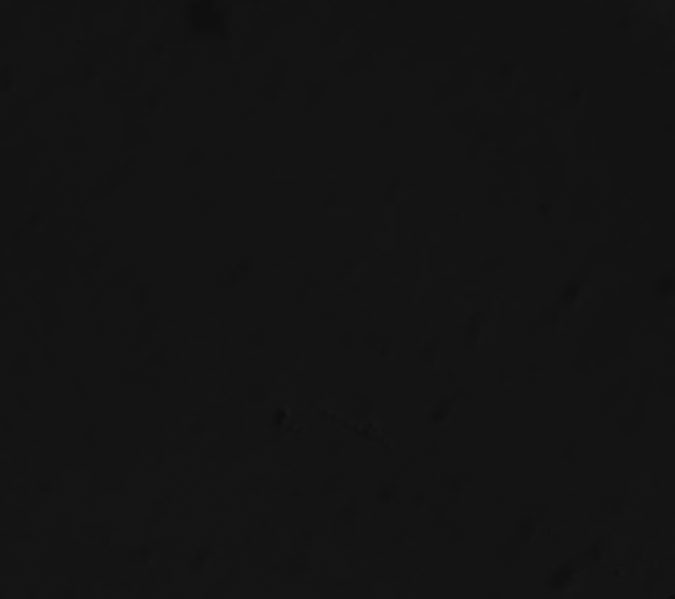

Supplement: Supplementary file 5 — Supplementary Code [file 41467_2023_36045_MOESM5_ESM.zip › Source Code/Untreated raw data for testing the code/PLGA NPs/Image340.jpg]

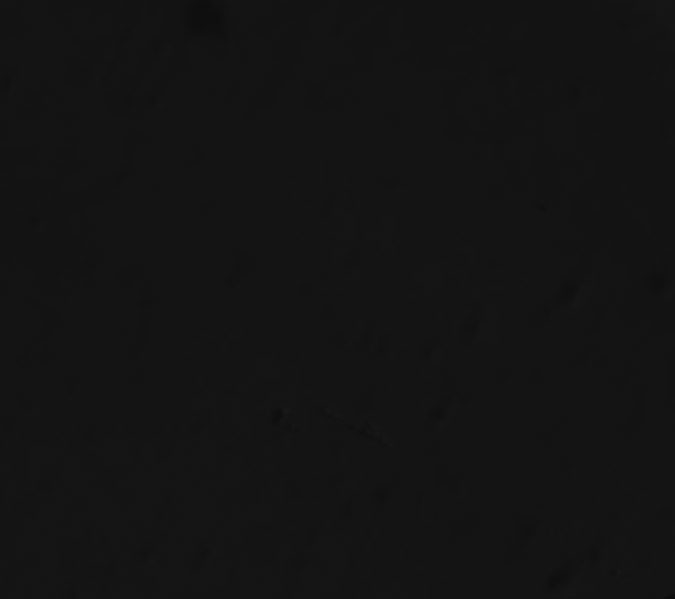

Supplement: Supplementary file 5 — Supplementary Code [file 41467_2023_36045_MOESM5_ESM.zip › Source Code/Untreated raw data for testing the code/PLGA NPs/Image426.jpg]

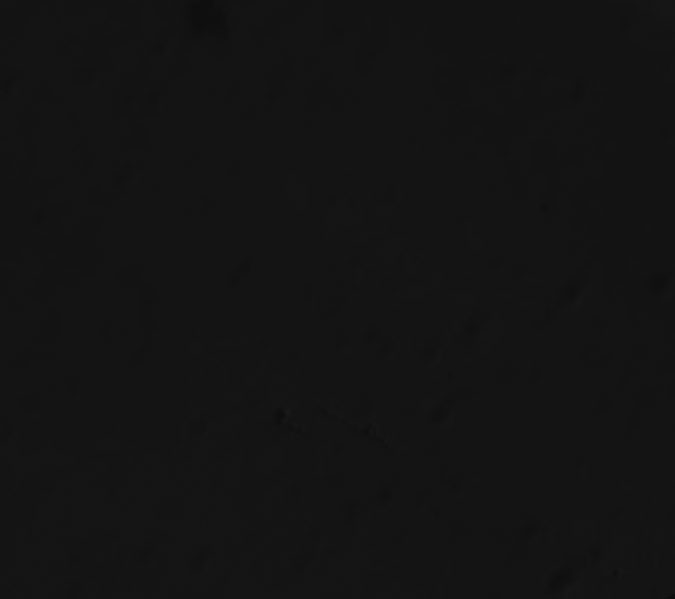

Supplement: Supplementary file 5 — Supplementary Code [file 41467_2023_36045_MOESM5_ESM.zip › Source Code/Untreated raw data for testing the code/PLGA NPs/Image432.jpg]

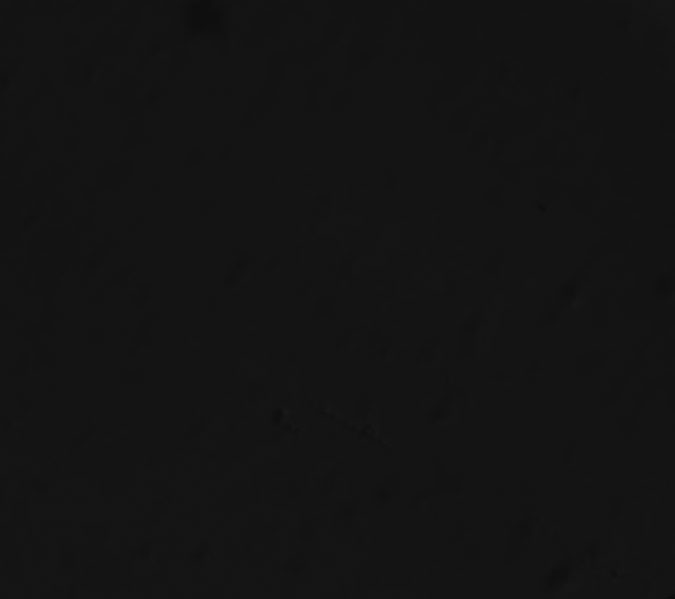

Supplement: Supplementary file 5 — Supplementary Code [file 41467_2023_36045_MOESM5_ESM.zip › Source Code/Untreated raw data for testing the code/PLGA NPs/Image354.jpg]

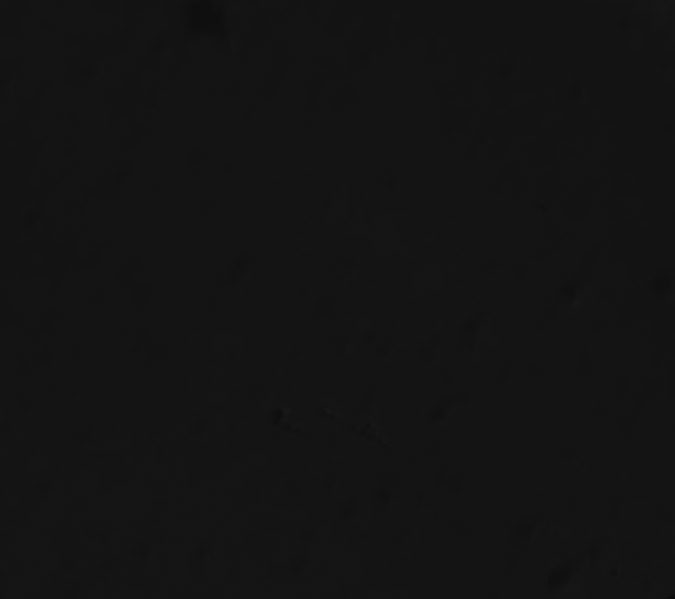

Supplement: Supplementary file 5 — Supplementary Code [file 41467_2023_36045_MOESM5_ESM.zip › Source Code/Untreated raw data for testing the code/PLGA NPs/Image368.jpg]
