# Supplementary figures and images for: In-situ cryo-immune engineering of tumor microenvironment with cold-responsive nanotechnology for cancer immunotherapy (part 1 of 2)
Source: Nat Commun. 2023 Jan 24;14:392. doi: 10.1038/s41467-023-36045-7 (PMC9873931; doi:10.1038/s41467-023-36045-7)

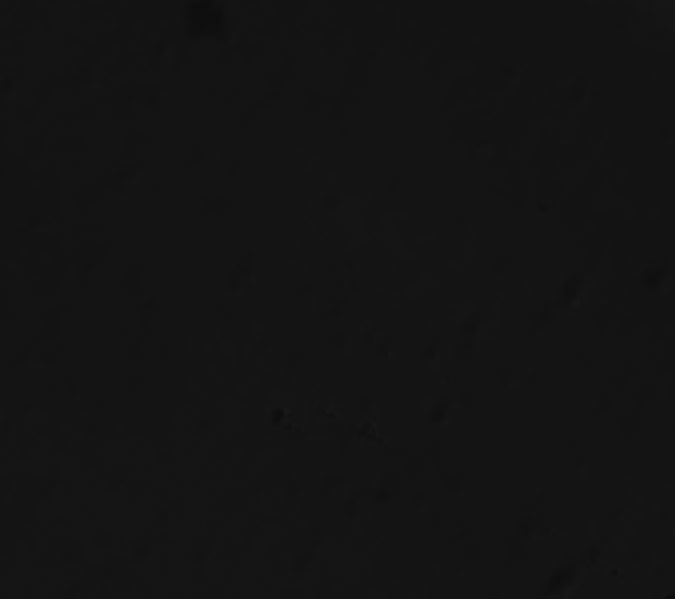

Supplement: Supplementary file 5 — Supplementary Code [file 41467_2023_36045_MOESM5_ESM.zip › Source Code/Untreated raw data for testing the code/PLGA NPs/Image478.jpg]

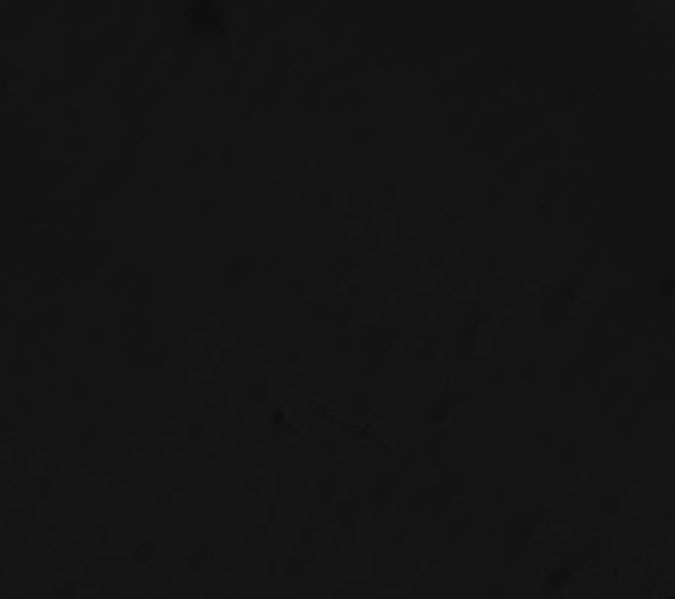

Supplement: Supplementary file 5 — Supplementary Code [file 41467_2023_36045_MOESM5_ESM.zip › Source Code/Untreated raw data for testing the code/PLGA NPs/Image336.jpg]

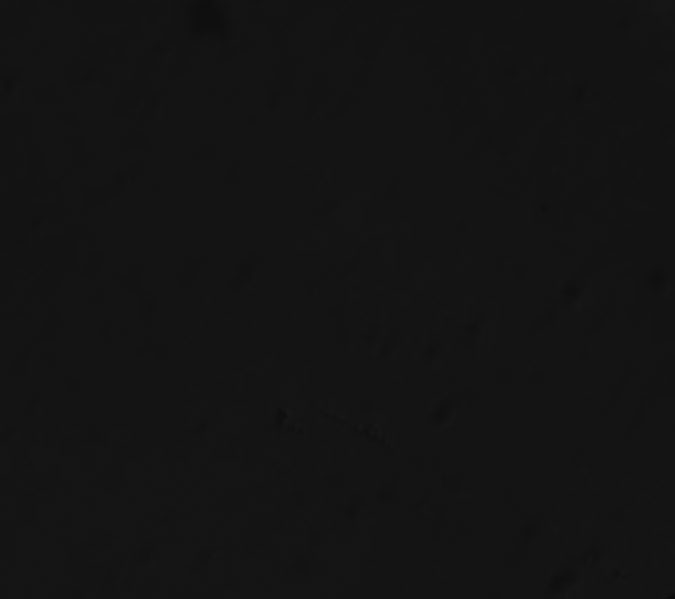

Supplement: Supplementary file 5 — Supplementary Code [file 41467_2023_36045_MOESM5_ESM.zip › Source Code/Untreated raw data for testing the code/PLGA NPs/Image450.jpg]

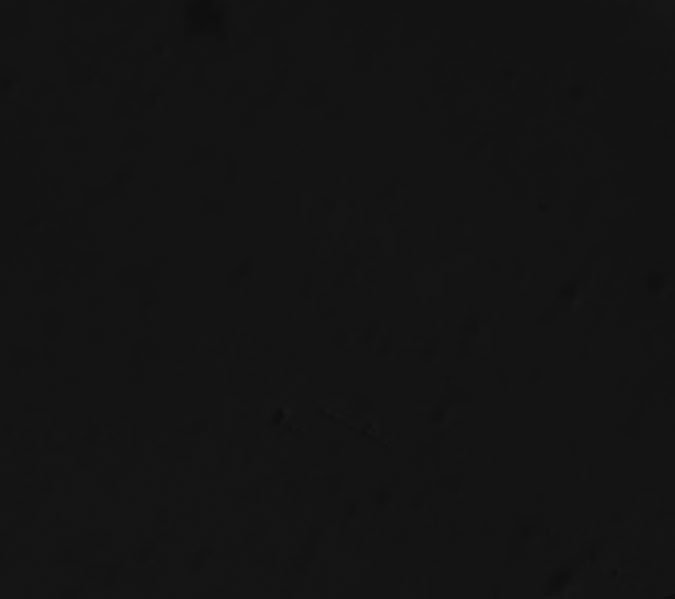

Supplement: Supplementary file 5 — Supplementary Code [file 41467_2023_36045_MOESM5_ESM.zip › Source Code/Untreated raw data for testing the code/PLGA NPs/Image444.jpg]

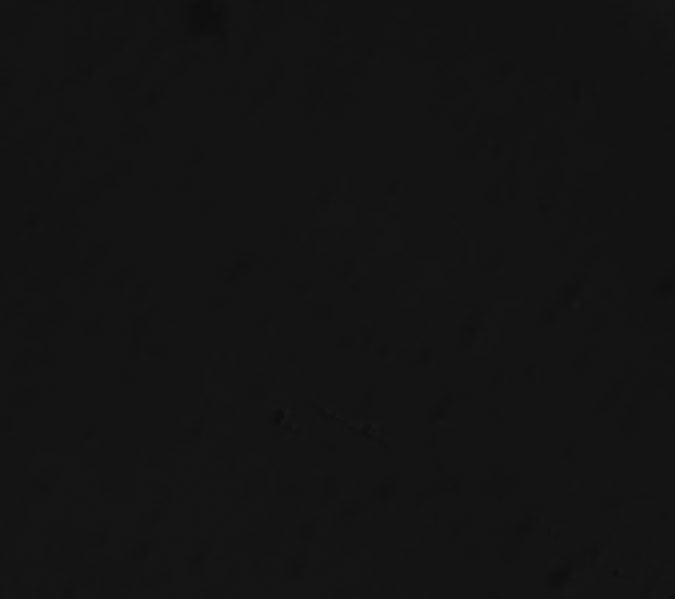

Supplement: Supplementary file 5 — Supplementary Code [file 41467_2023_36045_MOESM5_ESM.zip › Source Code/Untreated raw data for testing the code/PLGA NPs/Image322.jpg]

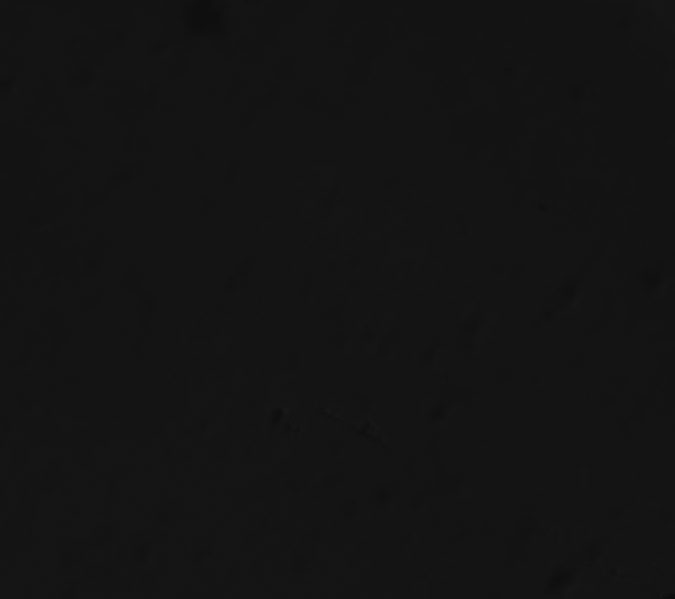

Supplement: Supplementary file 5 — Supplementary Code [file 41467_2023_36045_MOESM5_ESM.zip › Source Code/Untreated raw data for testing the code/PLGA NPs/Image493.jpg]

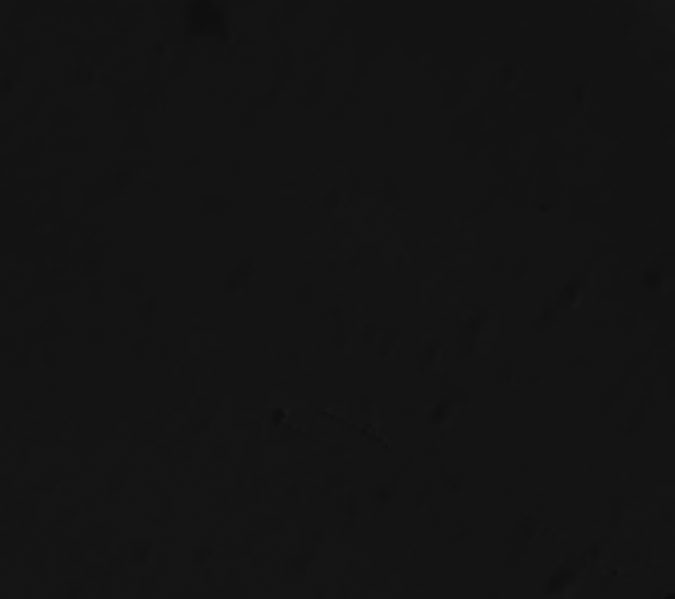

Supplement: Supplementary file 5 — Supplementary Code [file 41467_2023_36045_MOESM5_ESM.zip › Source Code/Untreated raw data for testing the code/PLGA NPs/Image487.jpg]

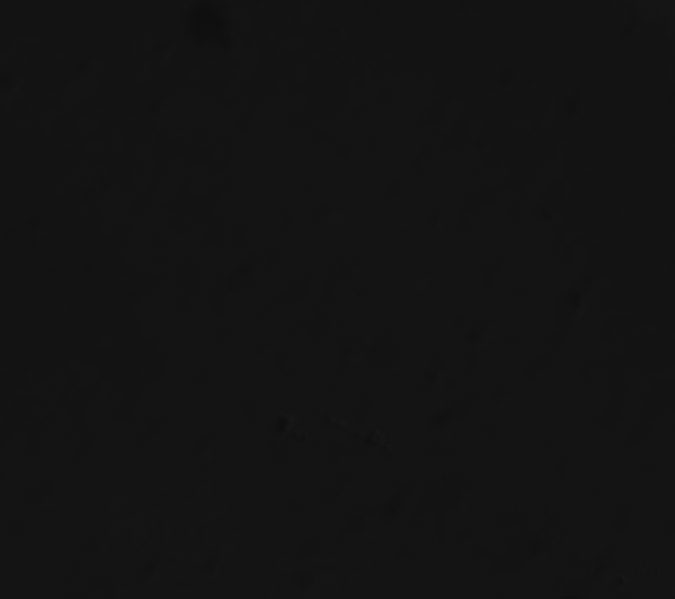

Supplement: Supplementary file 5 — Supplementary Code [file 41467_2023_36045_MOESM5_ESM.zip › Source Code/Untreated raw data for testing the code/PLGA NPs/Image108.jpg]

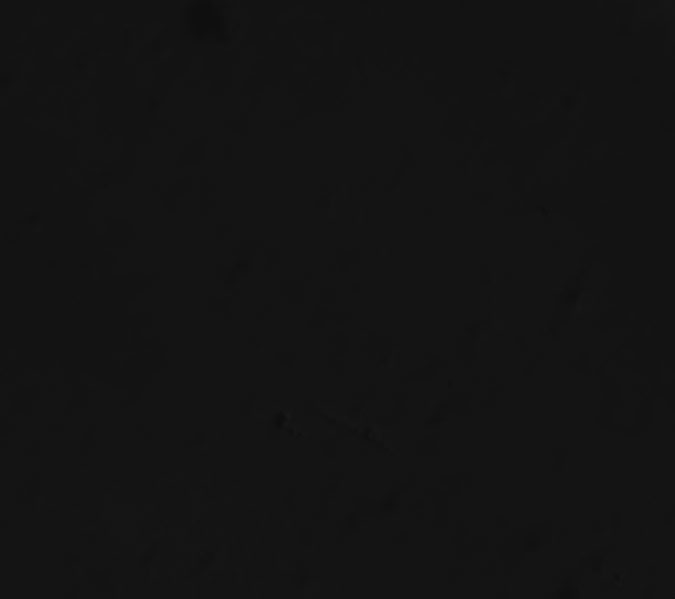

Supplement: Supplementary file 5 — Supplementary Code [file 41467_2023_36045_MOESM5_ESM.zip › Source Code/Untreated raw data for testing the code/PLGA NPs/Image134.jpg]

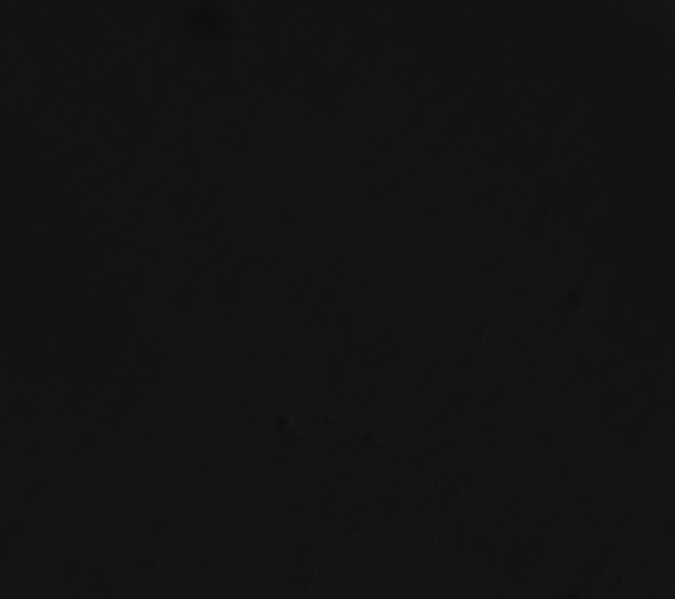

Supplement: Supplementary file 5 — Supplementary Code [file 41467_2023_36045_MOESM5_ESM.zip › Source Code/Untreated raw data for testing the code/PLGA NPs/Image120.jpg]

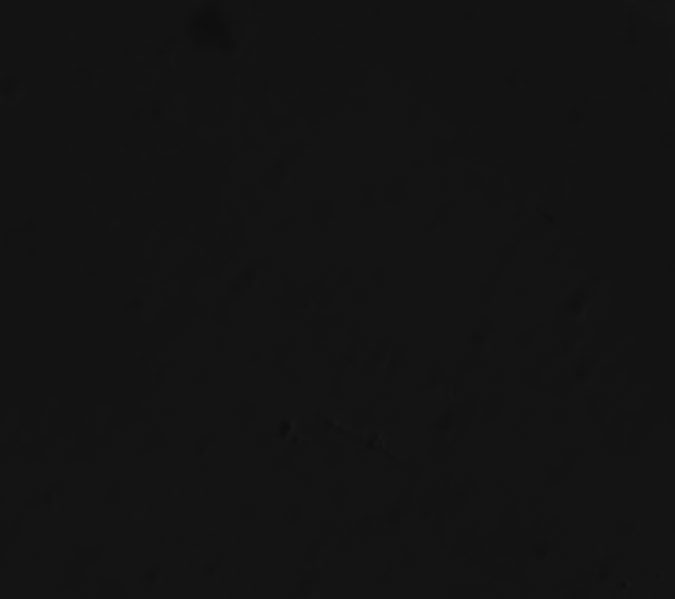

Supplement: Supplementary file 5 — Supplementary Code [file 41467_2023_36045_MOESM5_ESM.zip › Source Code/Untreated raw data for testing the code/PLGA NPs/Image63.jpg]

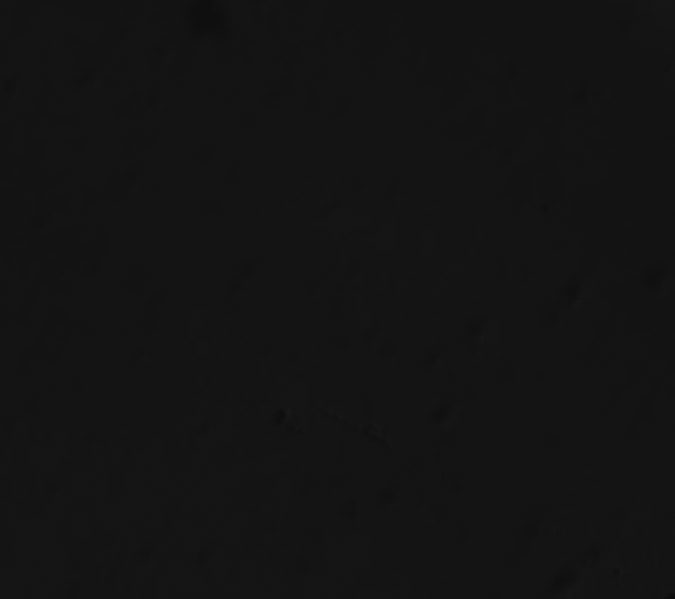

Supplement: Supplementary file 5 — Supplementary Code [file 41467_2023_36045_MOESM5_ESM.zip › Source Code/Untreated raw data for testing the code/PLGA NPs/Image518.jpg]

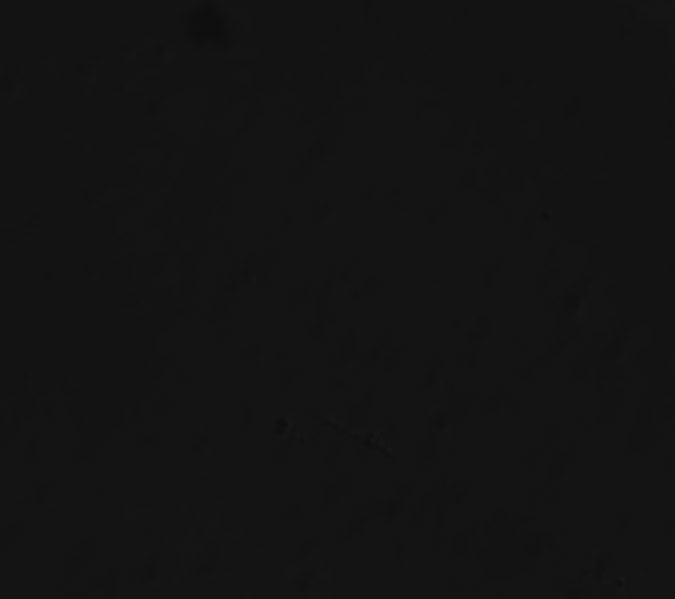

Supplement: Supplementary file 5 — Supplementary Code [file 41467_2023_36045_MOESM5_ESM.zip › Source Code/Untreated raw data for testing the code/PLGA NPs/Image77.jpg]

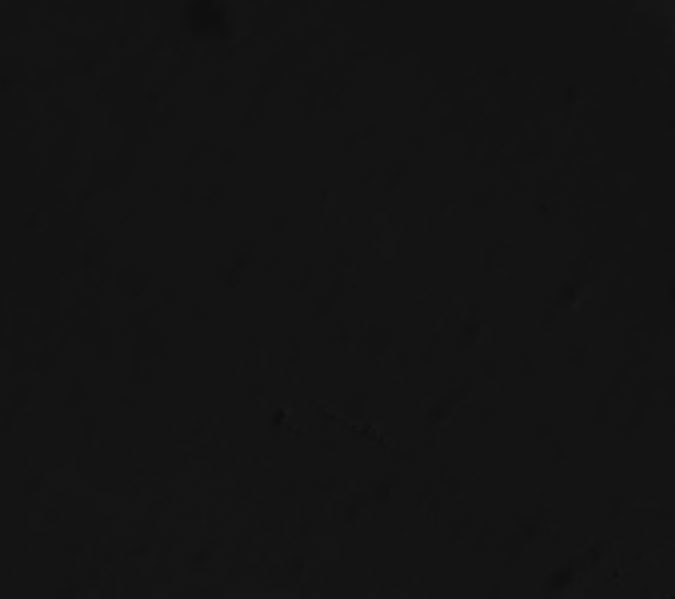

Supplement: Supplementary file 5 — Supplementary Code [file 41467_2023_36045_MOESM5_ESM.zip › Source Code/Untreated raw data for testing the code/PLGA NPs/Image242.jpg]

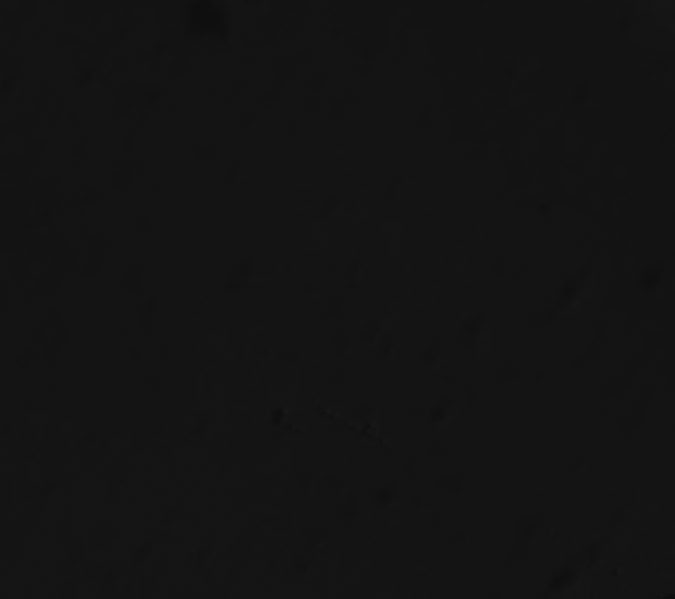

Supplement: Supplementary file 5 — Supplementary Code [file 41467_2023_36045_MOESM5_ESM.zip › Source Code/Untreated raw data for testing the code/PLGA NPs/Image524.jpg]

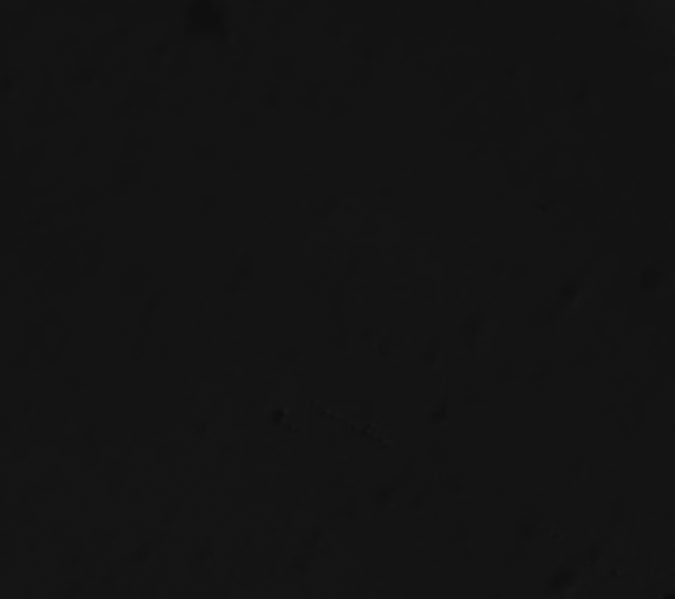

Supplement: Supplementary file 5 — Supplementary Code [file 41467_2023_36045_MOESM5_ESM.zip › Source Code/Untreated raw data for testing the code/PLGA NPs/Image530.jpg]

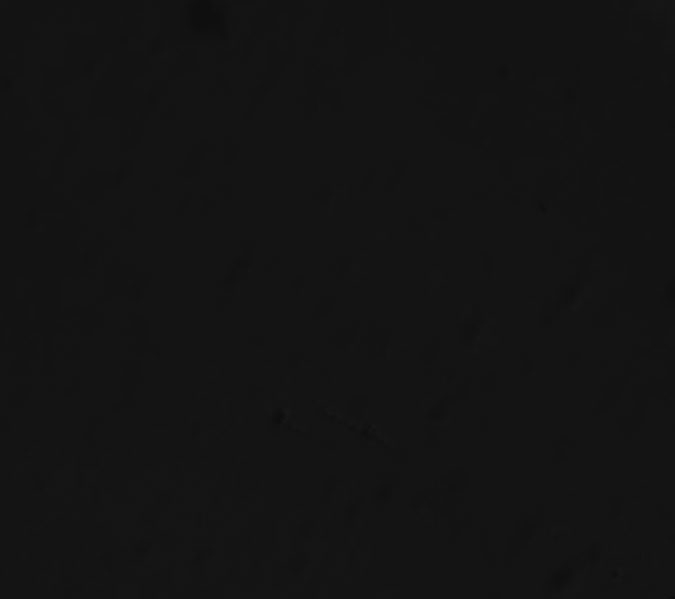

Supplement: Supplementary file 5 — Supplementary Code [file 41467_2023_36045_MOESM5_ESM.zip › Source Code/Untreated raw data for testing the code/PLGA NPs/Image256.jpg]

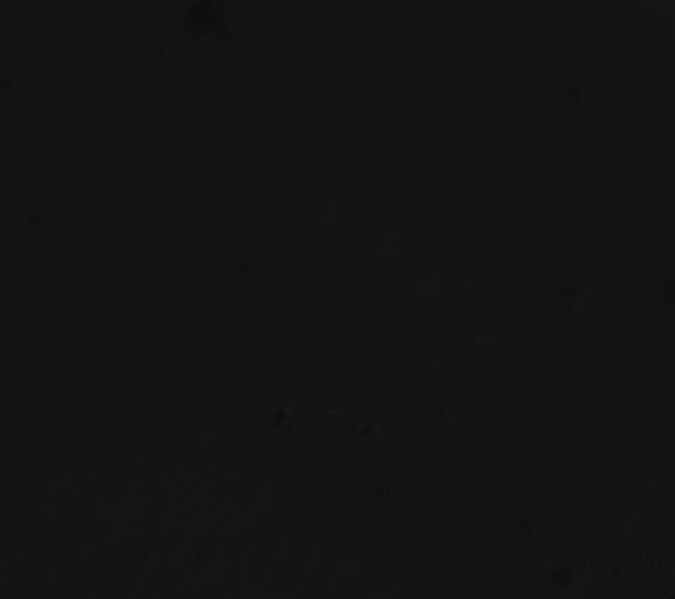

Supplement: Supplementary file 5 — Supplementary Code [file 41467_2023_36045_MOESM5_ESM.zip › Source Code/Untreated raw data for testing the code/PLGA NPs/Image281.jpg]

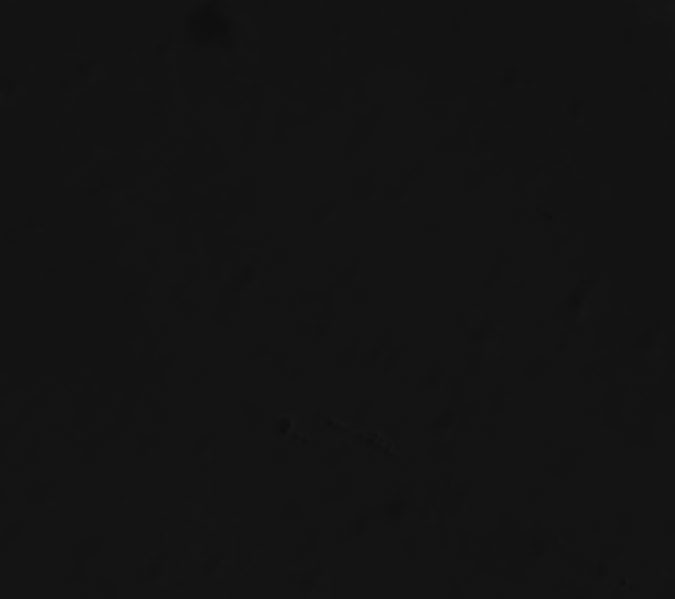

Supplement: Supplementary file 5 — Supplementary Code [file 41467_2023_36045_MOESM5_ESM.zip › Source Code/Untreated raw data for testing the code/PLGA NPs/Image88.jpg]

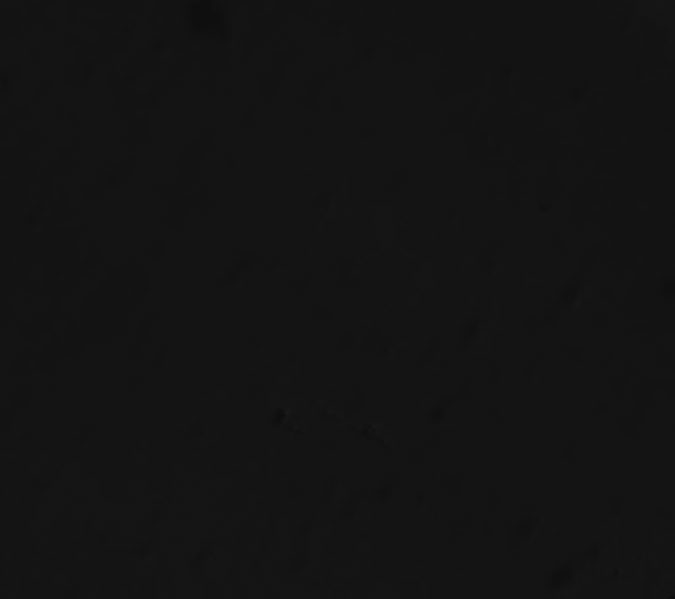

Supplement: Supplementary file 5 — Supplementary Code [file 41467_2023_36045_MOESM5_ESM.zip › Source Code/Untreated raw data for testing the code/PLGA NPs/Image295.jpg]

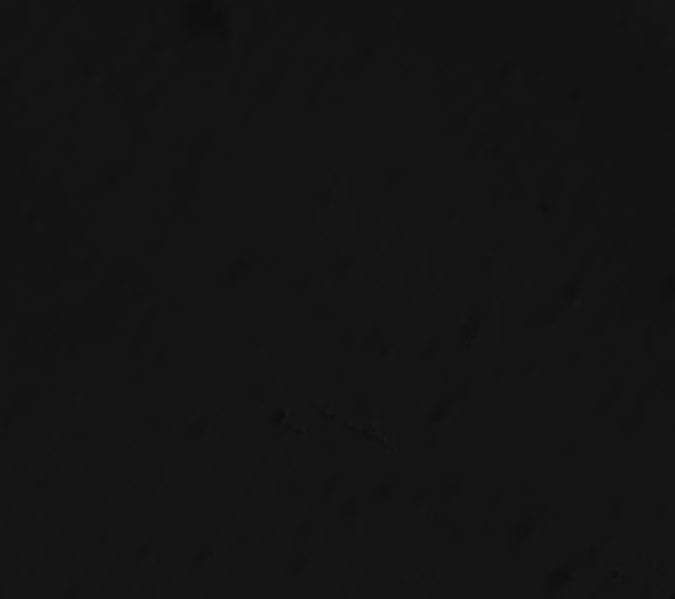

Supplement: Supplementary file 5 — Supplementary Code [file 41467_2023_36045_MOESM5_ESM.zip › Source Code/Untreated raw data for testing the code/PLGA NPs/Image294.jpg]

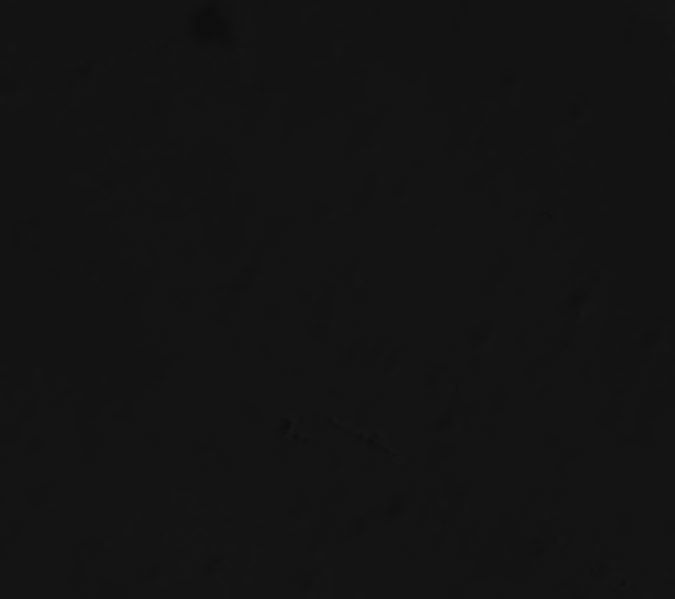

Supplement: Supplementary file 5 — Supplementary Code [file 41467_2023_36045_MOESM5_ESM.zip › Source Code/Untreated raw data for testing the code/PLGA NPs/Image89.jpg]

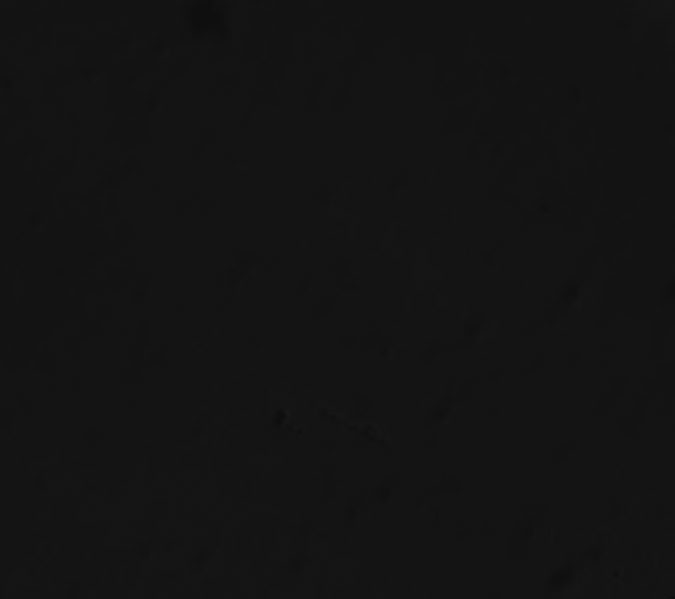

Supplement: Supplementary file 5 — Supplementary Code [file 41467_2023_36045_MOESM5_ESM.zip › Source Code/Untreated raw data for testing the code/PLGA NPs/Image280.jpg]

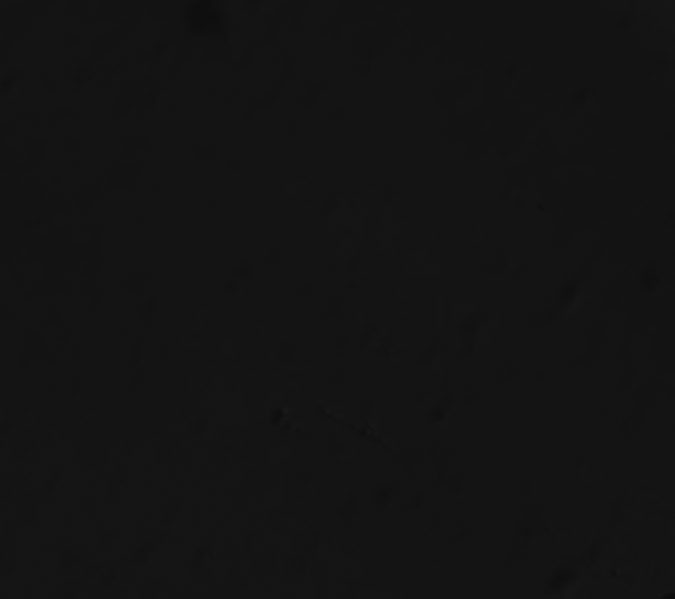

Supplement: Supplementary file 5 — Supplementary Code [file 41467_2023_36045_MOESM5_ESM.zip › Source Code/Untreated raw data for testing the code/PLGA NPs/Image531.jpg]

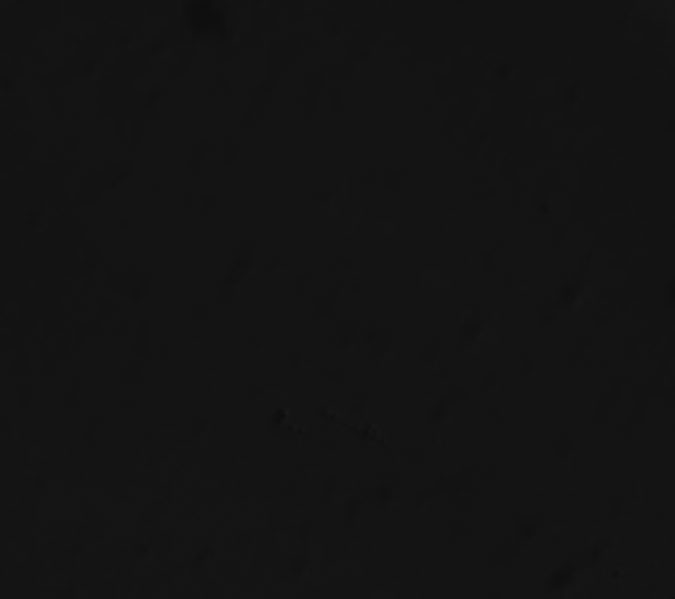

Supplement: Supplementary file 5 — Supplementary Code [file 41467_2023_36045_MOESM5_ESM.zip › Source Code/Untreated raw data for testing the code/PLGA NPs/Image257.jpg]

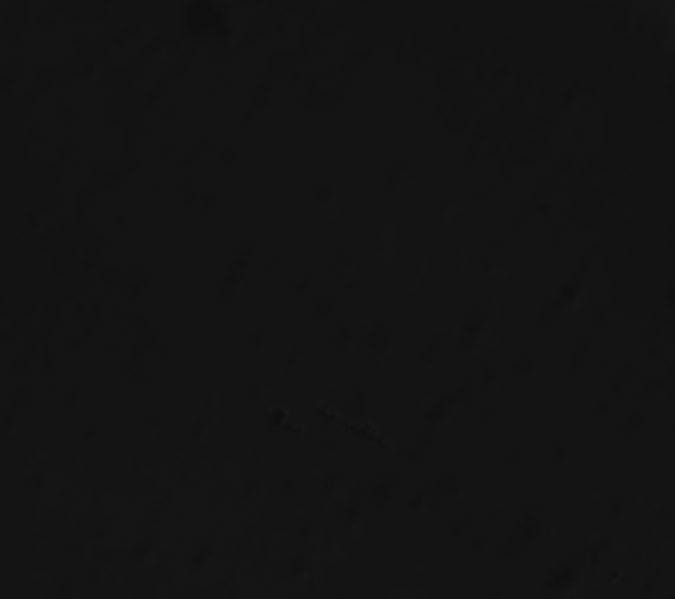

Supplement: Supplementary file 5 — Supplementary Code [file 41467_2023_36045_MOESM5_ESM.zip › Source Code/Untreated raw data for testing the code/PLGA NPs/Image243.jpg]

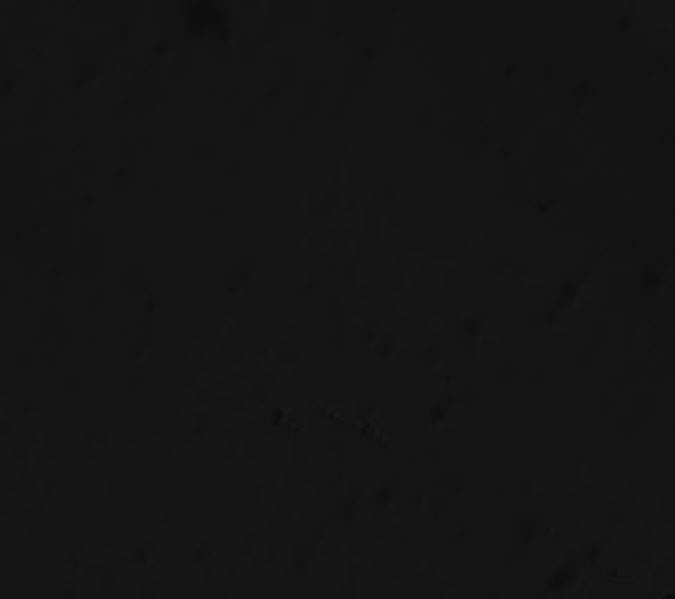

Supplement: Supplementary file 5 — Supplementary Code [file 41467_2023_36045_MOESM5_ESM.zip › Source Code/Untreated raw data for testing the code/PLGA NPs/Image525.jpg]

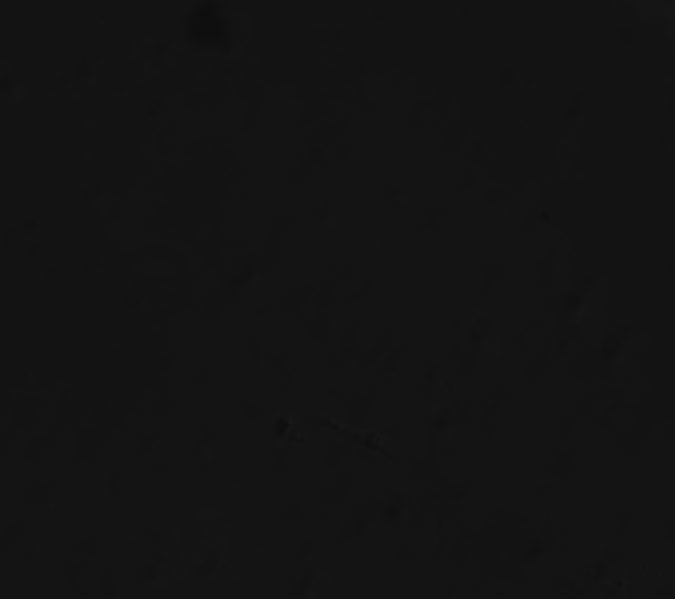

Supplement: Supplementary file 5 — Supplementary Code [file 41467_2023_36045_MOESM5_ESM.zip › Source Code/Untreated raw data for testing the code/PLGA NPs/Image76.jpg]

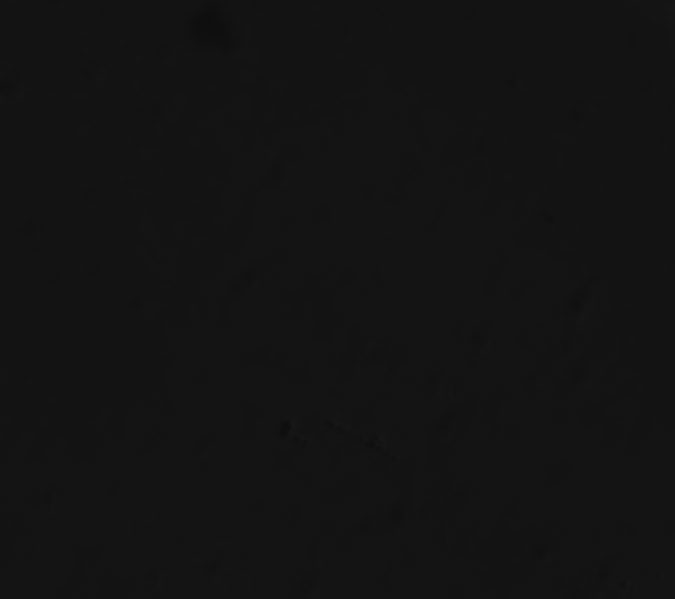

Supplement: Supplementary file 5 — Supplementary Code [file 41467_2023_36045_MOESM5_ESM.zip › Source Code/Untreated raw data for testing the code/PLGA NPs/Image62.jpg]

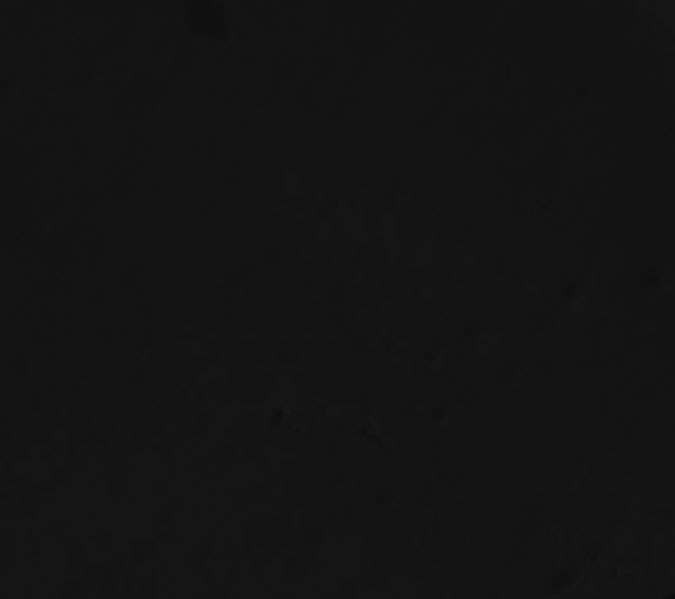

Supplement: Supplementary file 5 — Supplementary Code [file 41467_2023_36045_MOESM5_ESM.zip › Source Code/Untreated raw data for testing the code/PLGA NPs/Image519.jpg]

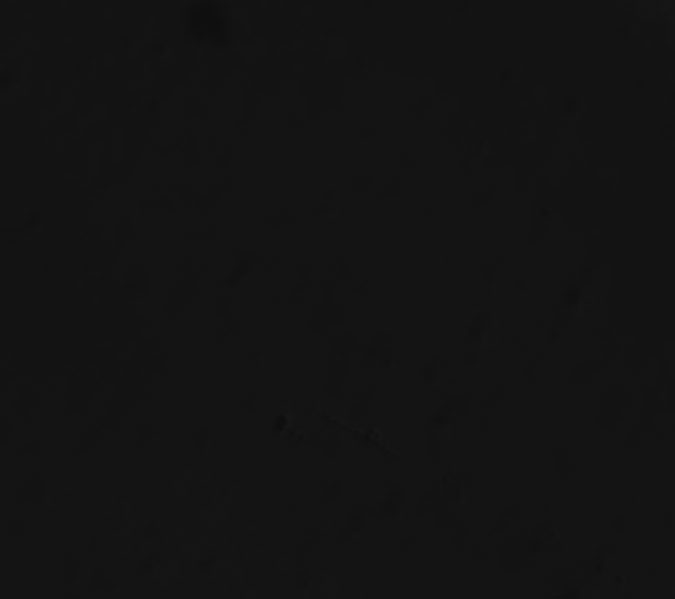

Supplement: Supplementary file 5 — Supplementary Code [file 41467_2023_36045_MOESM5_ESM.zip › Source Code/Untreated raw data for testing the code/PLGA NPs/Image121.jpg]

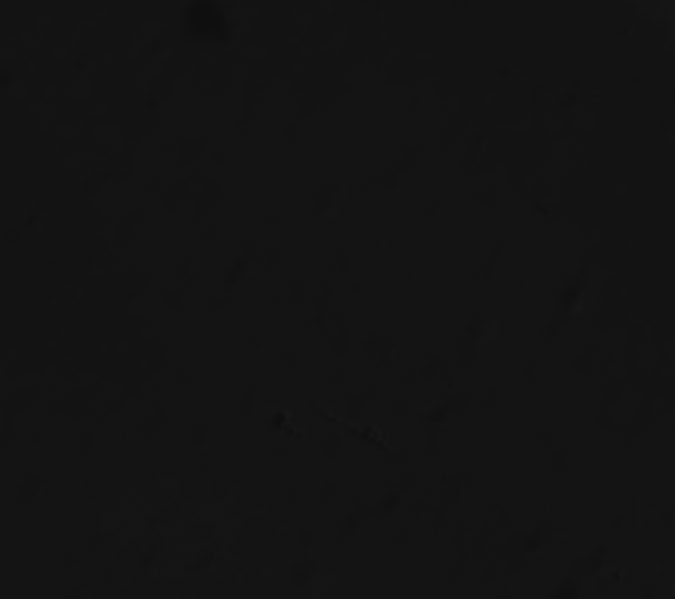

Supplement: Supplementary file 5 — Supplementary Code [file 41467_2023_36045_MOESM5_ESM.zip › Source Code/Untreated raw data for testing the code/PLGA NPs/Image135.jpg]

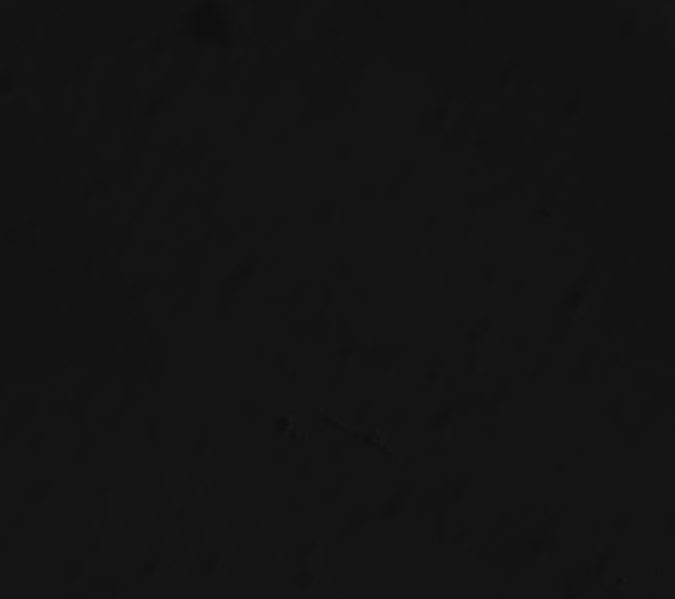

Supplement: Supplementary file 5 — Supplementary Code [file 41467_2023_36045_MOESM5_ESM.zip › Source Code/Untreated raw data for testing the code/PLGA NPs/Image109.jpg]

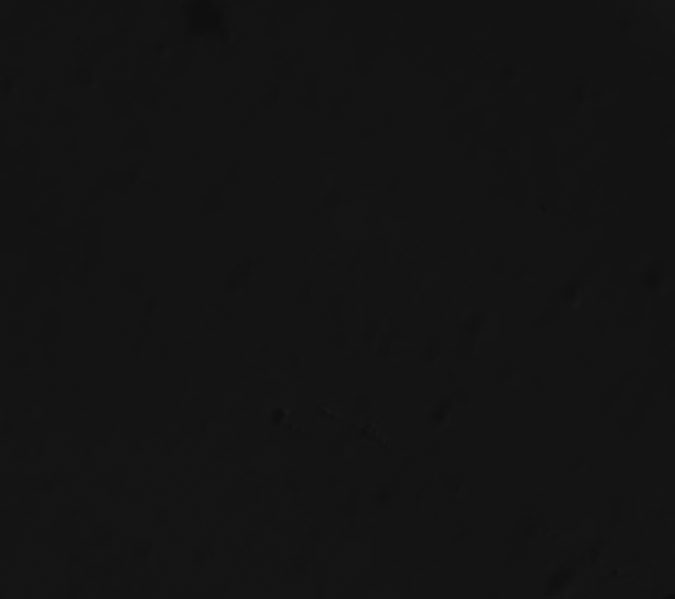

Supplement: Supplementary file 5 — Supplementary Code [file 41467_2023_36045_MOESM5_ESM.zip › Source Code/Untreated raw data for testing the code/PLGA NPs/Image486.jpg]

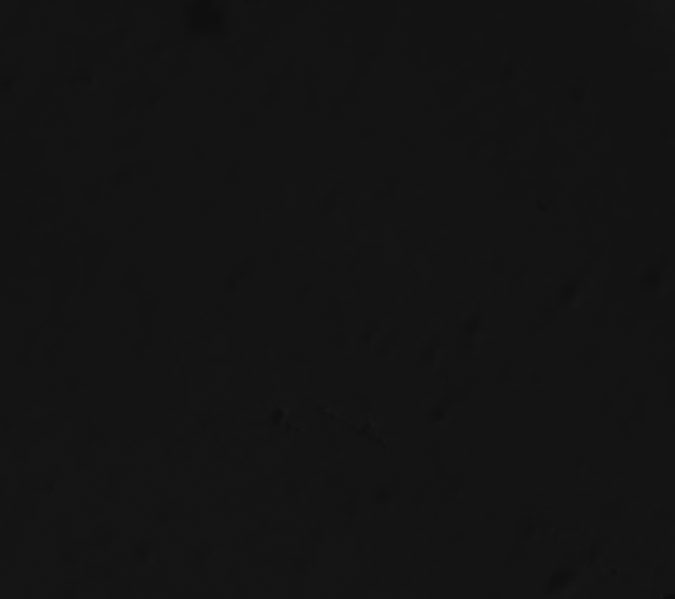

Supplement: Supplementary file 5 — Supplementary Code [file 41467_2023_36045_MOESM5_ESM.zip › Source Code/Untreated raw data for testing the code/PLGA NPs/Image492.jpg]

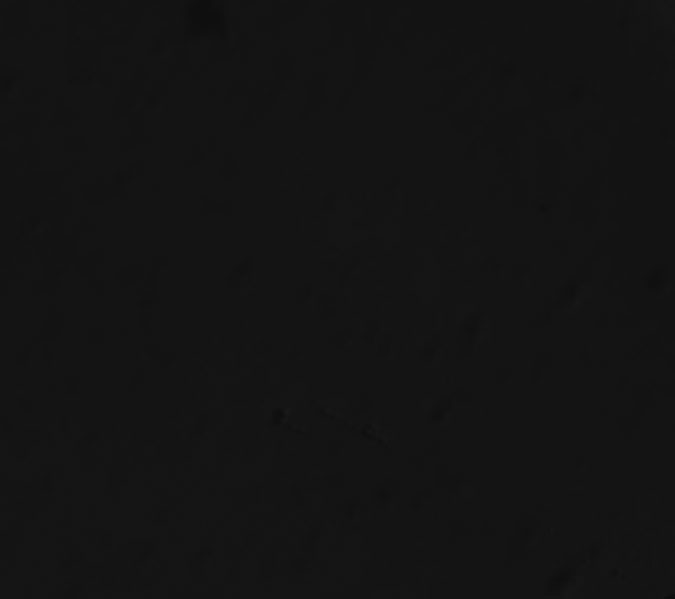

Supplement: Supplementary file 5 — Supplementary Code [file 41467_2023_36045_MOESM5_ESM.zip › Source Code/Untreated raw data for testing the code/PLGA NPs/Image445.jpg]

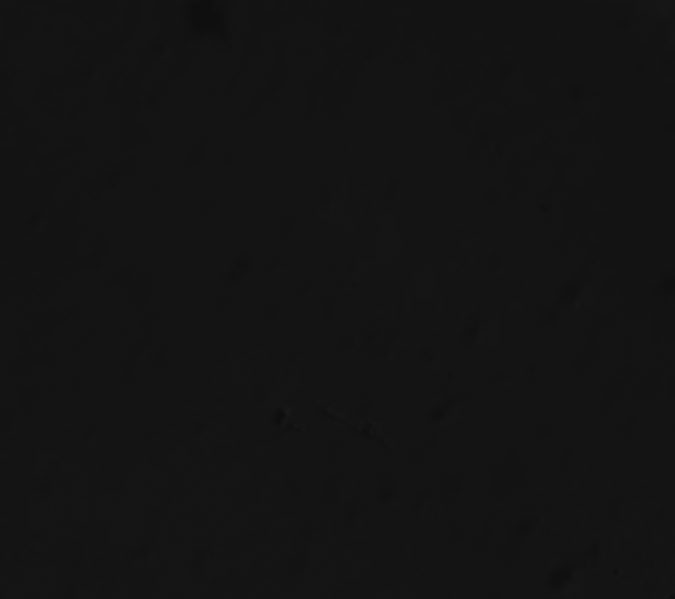

Supplement: Supplementary file 5 — Supplementary Code [file 41467_2023_36045_MOESM5_ESM.zip › Source Code/Untreated raw data for testing the code/PLGA NPs/Image323.jpg]

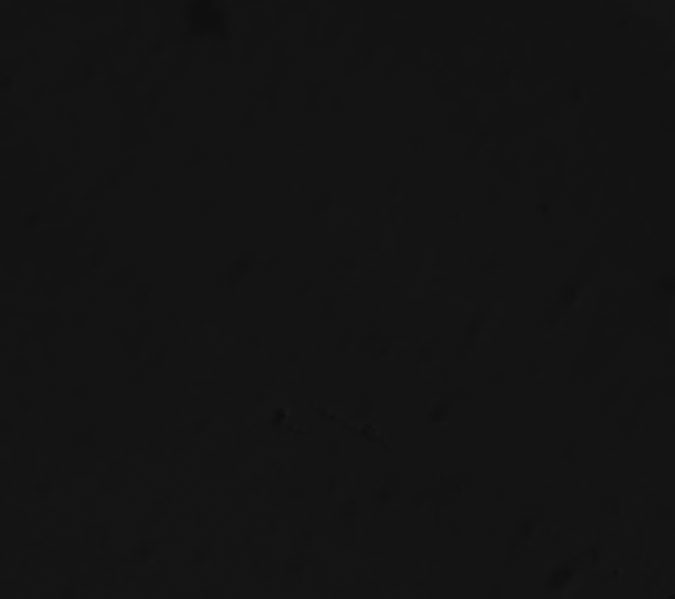

Supplement: Supplementary file 5 — Supplementary Code [file 41467_2023_36045_MOESM5_ESM.zip › Source Code/Untreated raw data for testing the code/PLGA NPs/Image337.jpg]

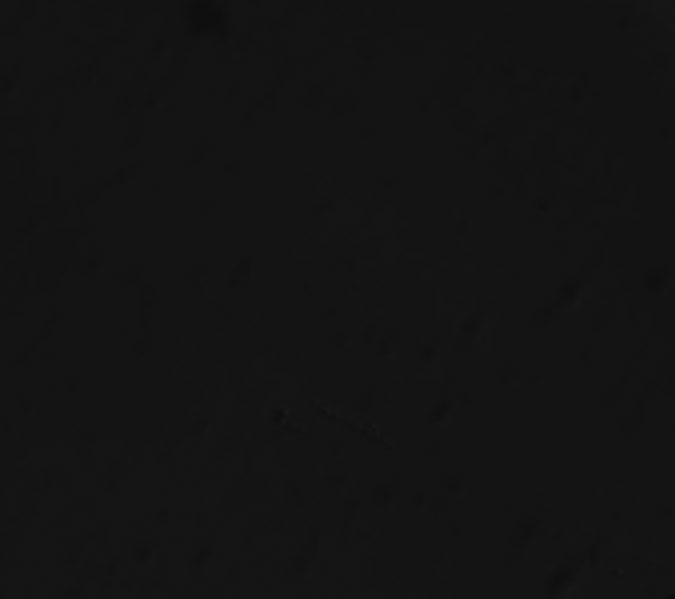

Supplement: Supplementary file 5 — Supplementary Code [file 41467_2023_36045_MOESM5_ESM.zip › Source Code/Untreated raw data for testing the code/PLGA NPs/Image451.jpg]

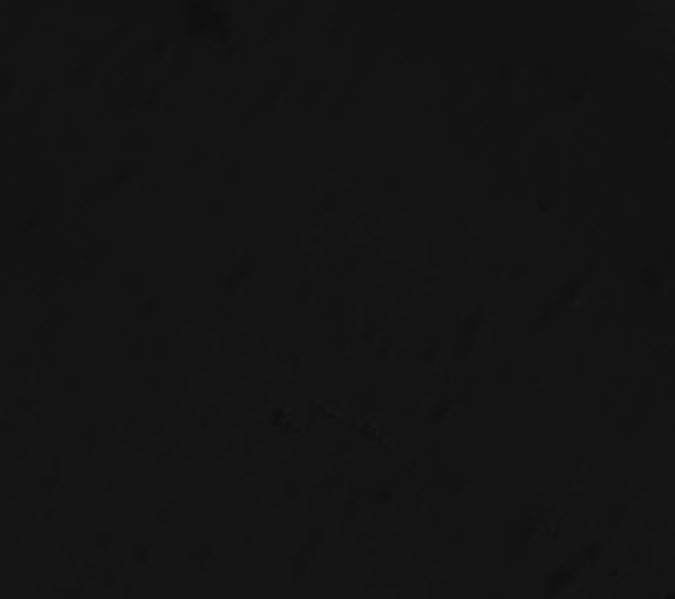

Supplement: Supplementary file 5 — Supplementary Code [file 41467_2023_36045_MOESM5_ESM.zip › Source Code/Untreated raw data for testing the code/PLGA NPs/Image479.jpg]

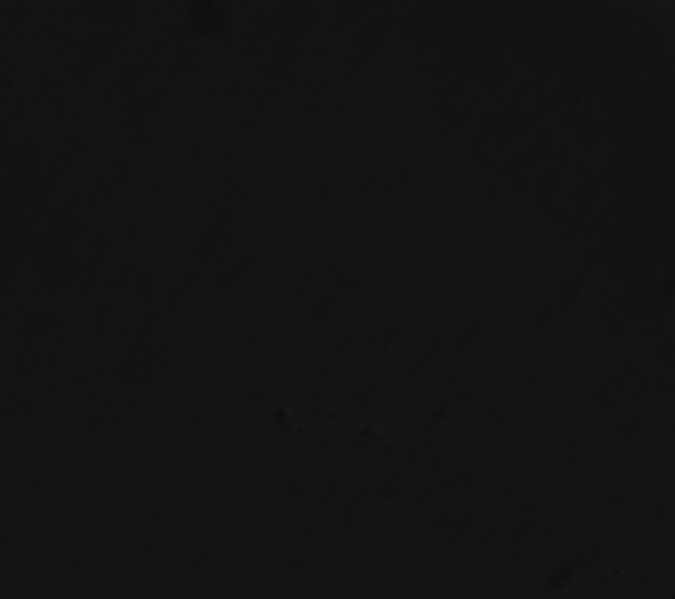

Supplement: Supplementary file 5 — Supplementary Code [file 41467_2023_36045_MOESM5_ESM.zip › Source Code/Untreated raw data for testing the code/PLGA NPs/Image309.jpg]

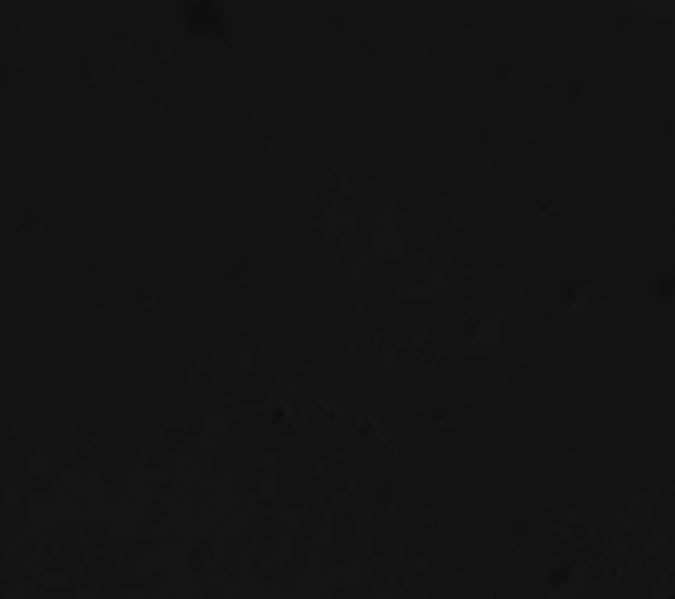

Supplement: Supplementary file 5 — Supplementary Code [file 41467_2023_36045_MOESM5_ESM.zip › Source Code/Untreated raw data for testing the code/PLGA NPs/Image321.jpg]

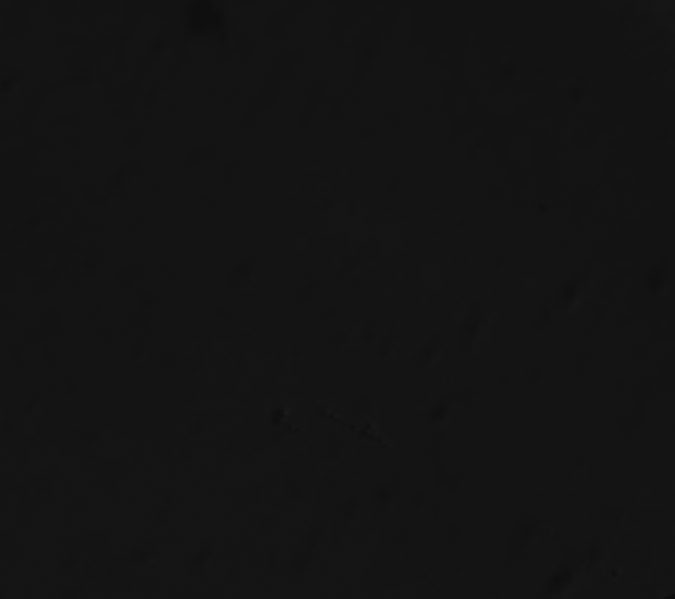

Supplement: Supplementary file 5 — Supplementary Code [file 41467_2023_36045_MOESM5_ESM.zip › Source Code/Untreated raw data for testing the code/PLGA NPs/Image447.jpg]

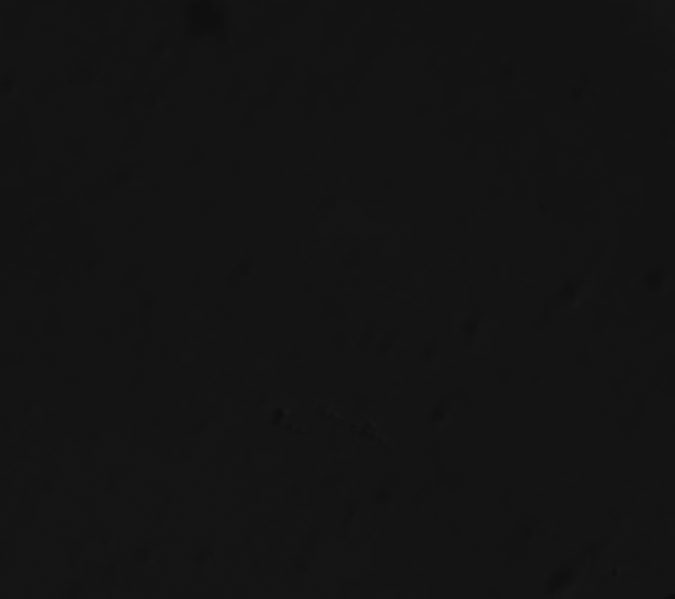

Supplement: Supplementary file 5 — Supplementary Code [file 41467_2023_36045_MOESM5_ESM.zip › Source Code/Untreated raw data for testing the code/PLGA NPs/Image453.jpg]

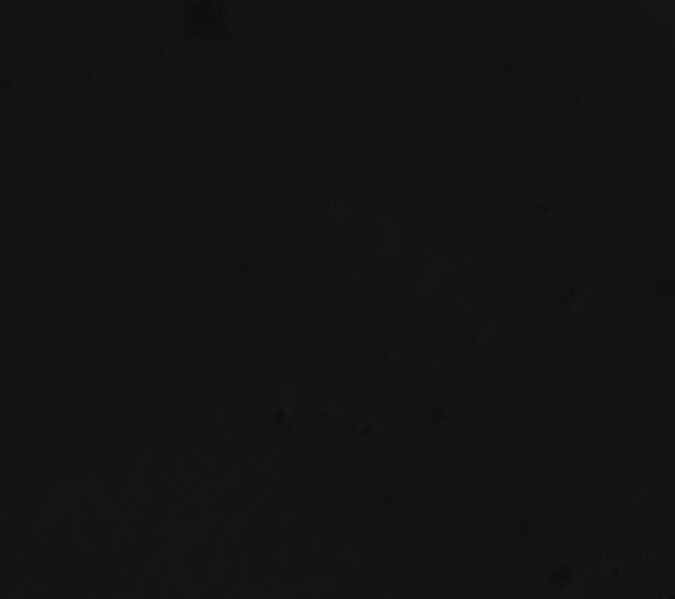

Supplement: Supplementary file 5 — Supplementary Code [file 41467_2023_36045_MOESM5_ESM.zip › Source Code/Untreated raw data for testing the code/PLGA NPs/Image335.jpg]

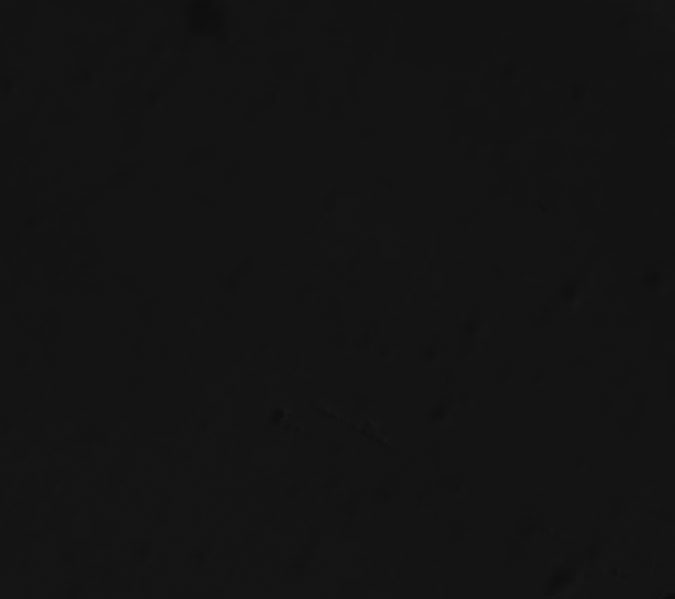

Supplement: Supplementary file 5 — Supplementary Code [file 41467_2023_36045_MOESM5_ESM.zip › Source Code/Untreated raw data for testing the code/PLGA NPs/Image484.jpg]

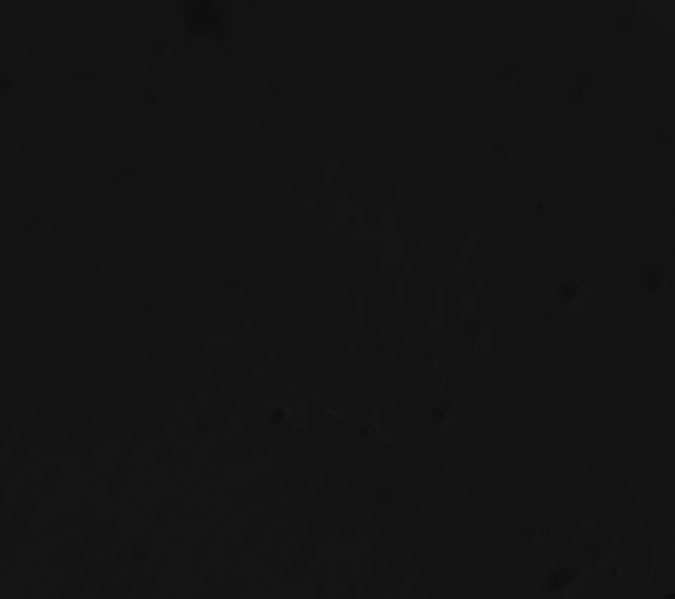

Supplement: Supplementary file 5 — Supplementary Code [file 41467_2023_36045_MOESM5_ESM.zip › Source Code/Untreated raw data for testing the code/PLGA NPs/Image490.jpg]

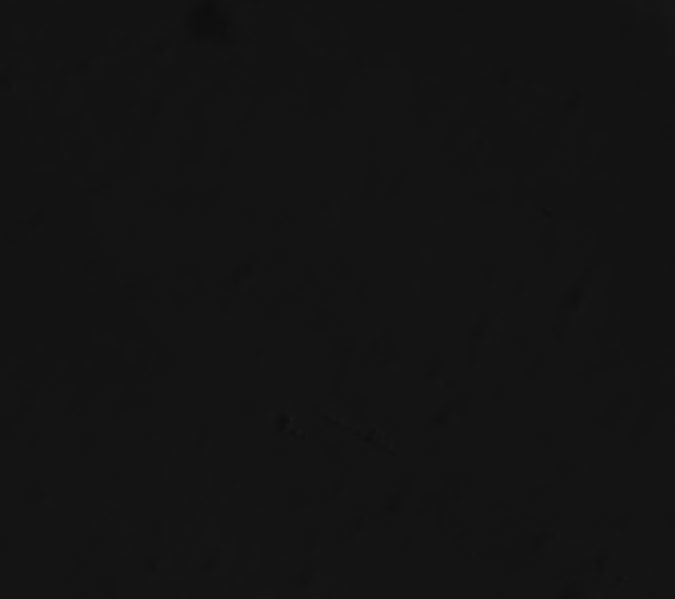

Supplement: Supplementary file 5 — Supplementary Code [file 41467_2023_36045_MOESM5_ESM.zip › Source Code/Untreated raw data for testing the code/PLGA NPs/Image123.jpg]

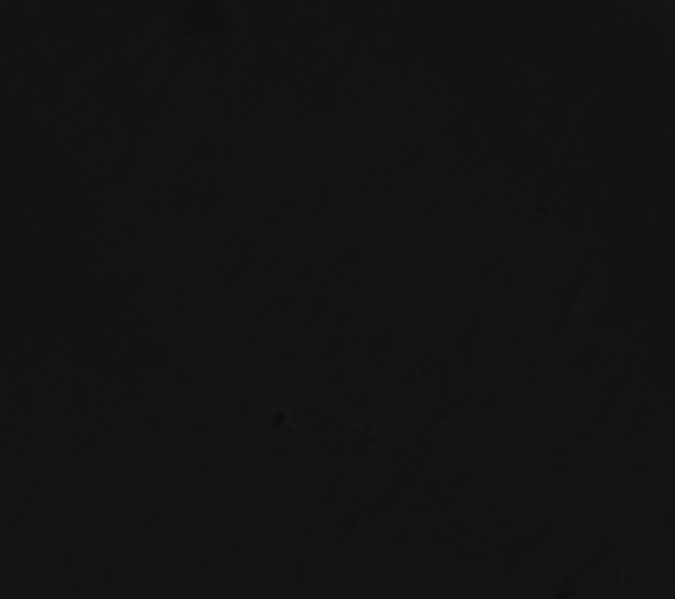

Supplement: Supplementary file 5 — Supplementary Code [file 41467_2023_36045_MOESM5_ESM.zip › Source Code/Untreated raw data for testing the code/PLGA NPs/Image137.jpg]

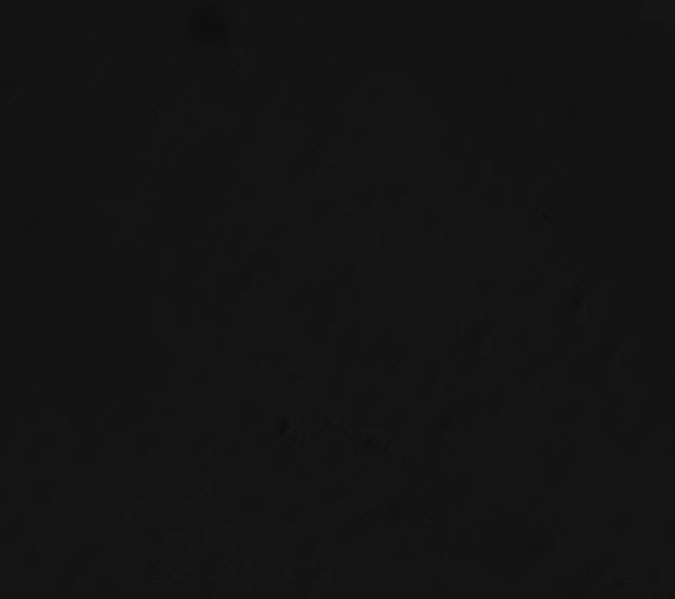

Supplement: Supplementary file 5 — Supplementary Code [file 41467_2023_36045_MOESM5_ESM.zip › Source Code/Untreated raw data for testing the code/PLGA NPs/Image74.jpg]

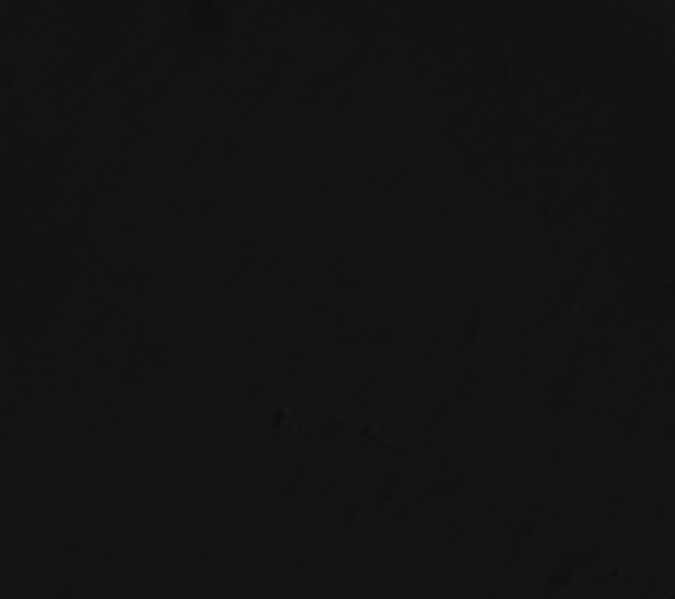

Supplement: Supplementary file 5 — Supplementary Code [file 41467_2023_36045_MOESM5_ESM.zip › Source Code/Untreated raw data for testing the code/PLGA NPs/Image269.jpg]

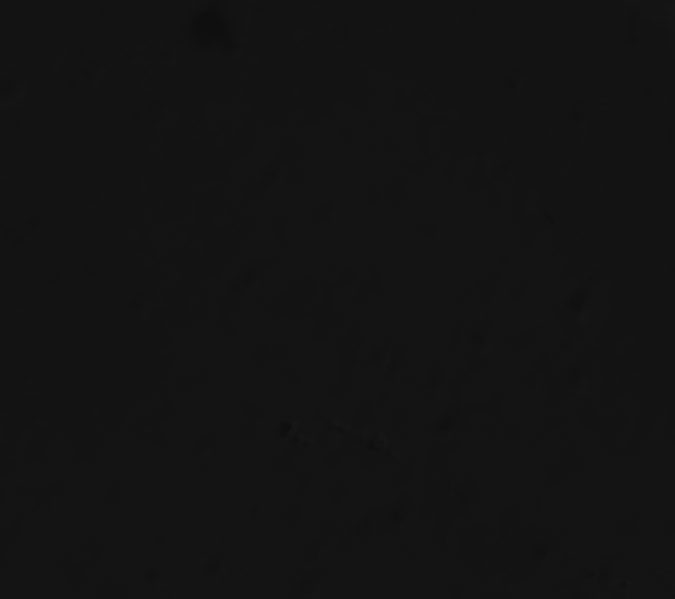

Supplement: Supplementary file 5 — Supplementary Code [file 41467_2023_36045_MOESM5_ESM.zip › Source Code/Untreated raw data for testing the code/PLGA NPs/Image60.jpg]

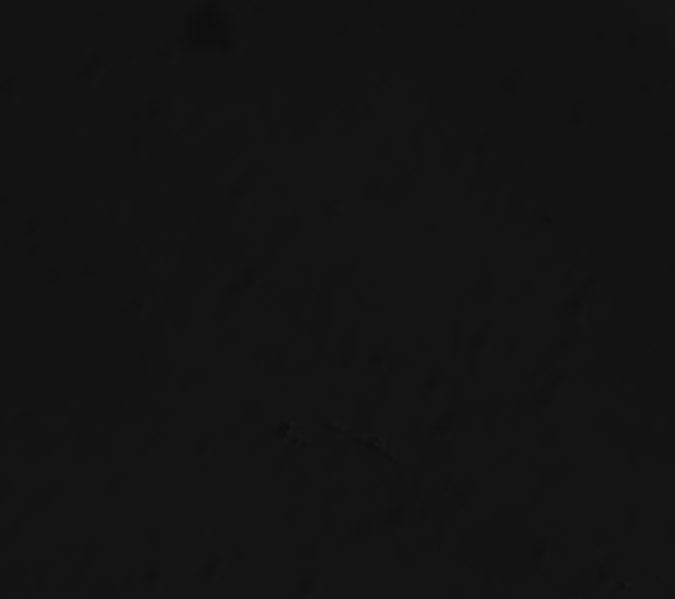

Supplement: Supplementary file 5 — Supplementary Code [file 41467_2023_36045_MOESM5_ESM.zip › Source Code/Untreated raw data for testing the code/PLGA NPs/Image48.jpg]

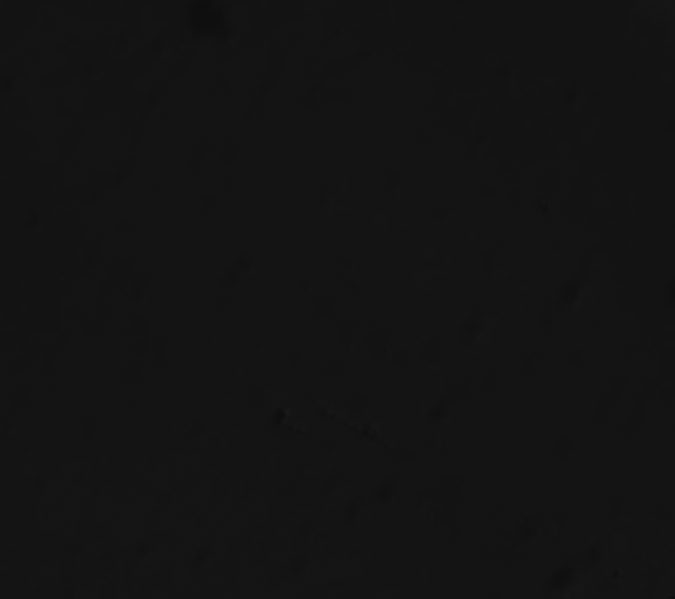

Supplement: Supplementary file 5 — Supplementary Code [file 41467_2023_36045_MOESM5_ESM.zip › Source Code/Untreated raw data for testing the code/PLGA NPs/Image255.jpg]

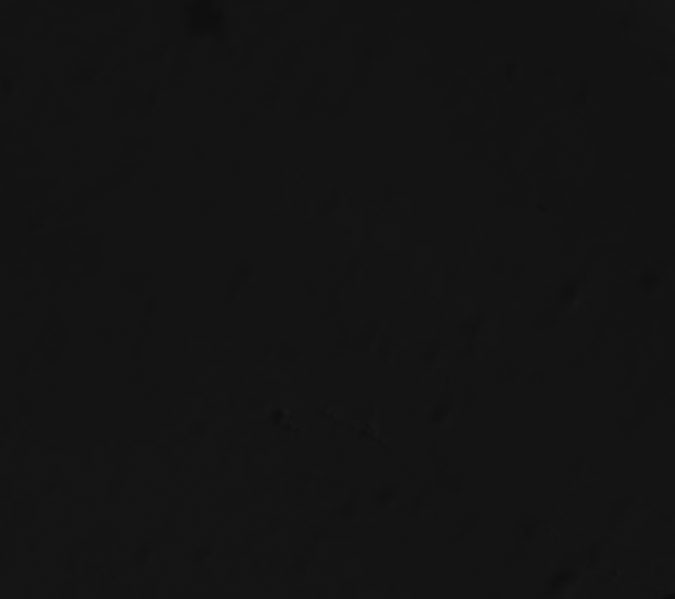

Supplement: Supplementary file 5 — Supplementary Code [file 41467_2023_36045_MOESM5_ESM.zip › Source Code/Untreated raw data for testing the code/PLGA NPs/Image533.jpg]

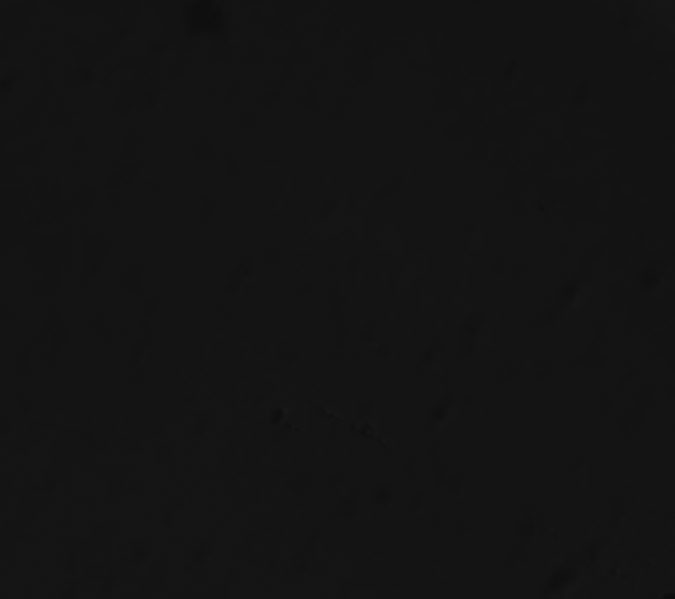

Supplement: Supplementary file 5 — Supplementary Code [file 41467_2023_36045_MOESM5_ESM.zip › Source Code/Untreated raw data for testing the code/PLGA NPs/Image527.jpg]

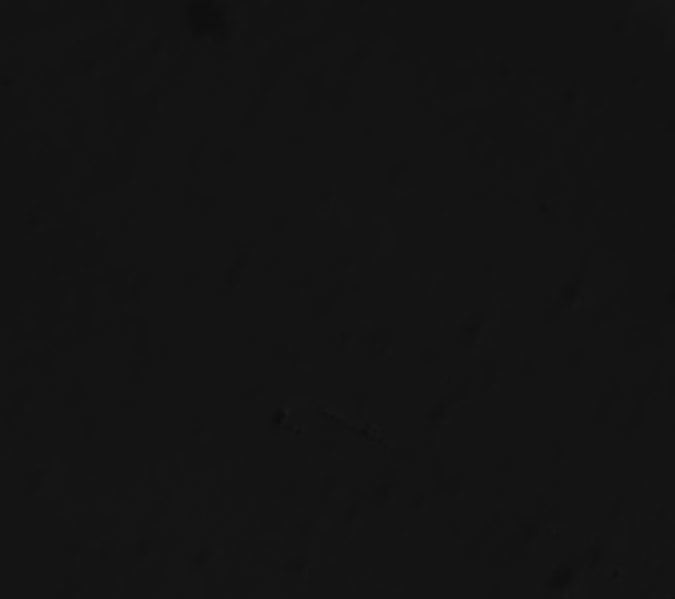

Supplement: Supplementary file 5 — Supplementary Code [file 41467_2023_36045_MOESM5_ESM.zip › Source Code/Untreated raw data for testing the code/PLGA NPs/Image241.jpg]

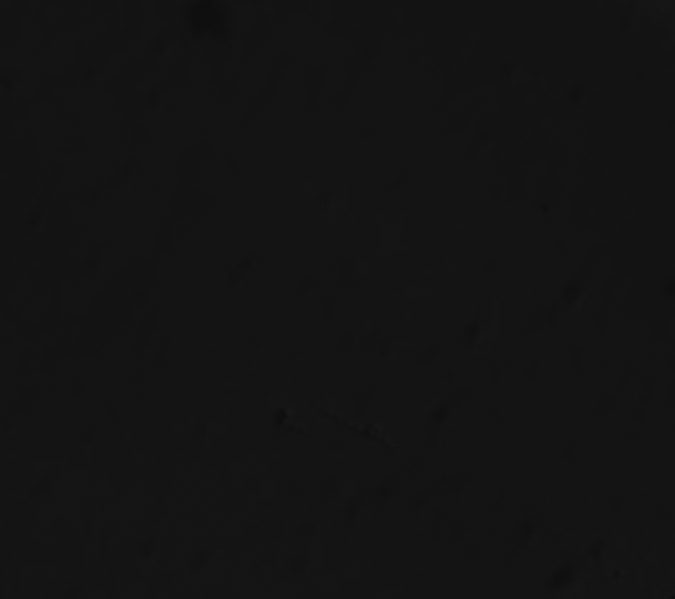

Supplement: Supplementary file 5 — Supplementary Code [file 41467_2023_36045_MOESM5_ESM.zip › Source Code/Untreated raw data for testing the code/PLGA NPs/Image296.jpg]

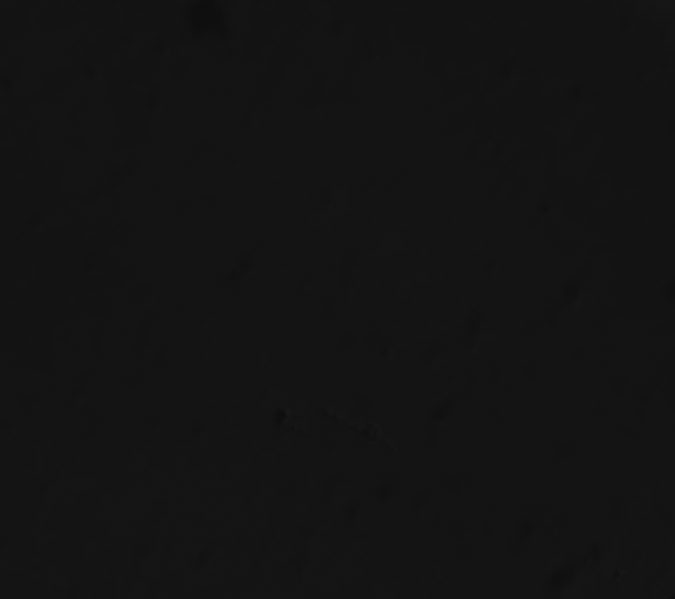

Supplement: Supplementary file 5 — Supplementary Code [file 41467_2023_36045_MOESM5_ESM.zip › Source Code/Untreated raw data for testing the code/PLGA NPs/Image282.jpg]

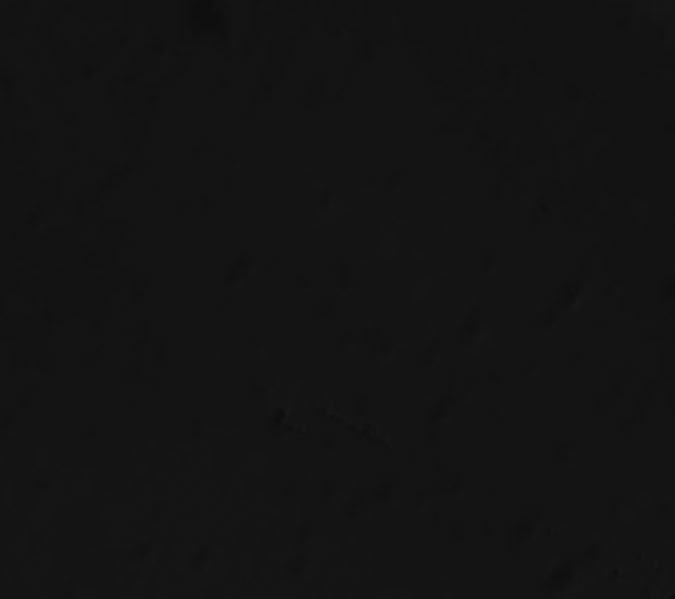

Supplement: Supplementary file 5 — Supplementary Code [file 41467_2023_36045_MOESM5_ESM.zip › Source Code/Untreated raw data for testing the code/PLGA NPs/Image283.jpg]

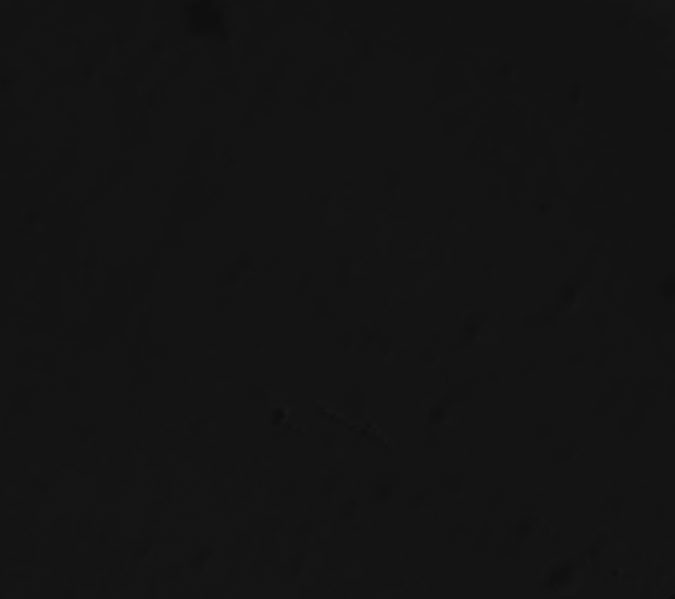

Supplement: Supplementary file 5 — Supplementary Code [file 41467_2023_36045_MOESM5_ESM.zip › Source Code/Untreated raw data for testing the code/PLGA NPs/Image297.jpg]

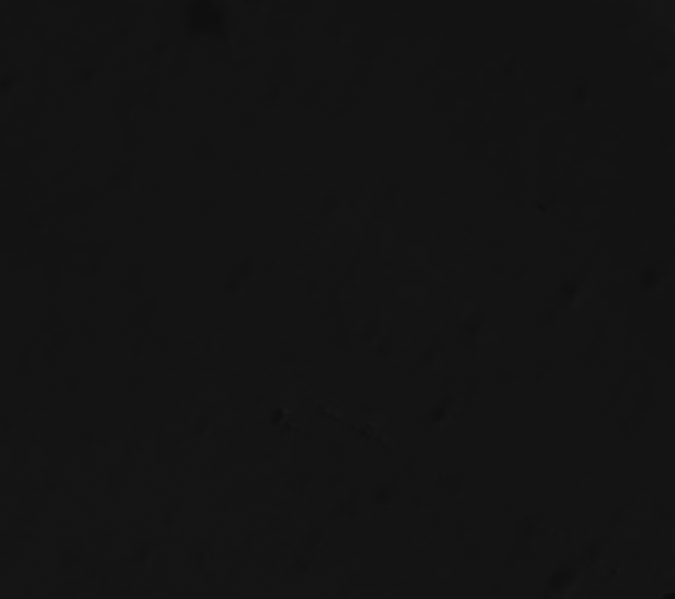

Supplement: Supplementary file 5 — Supplementary Code [file 41467_2023_36045_MOESM5_ESM.zip › Source Code/Untreated raw data for testing the code/PLGA NPs/Image526.jpg]

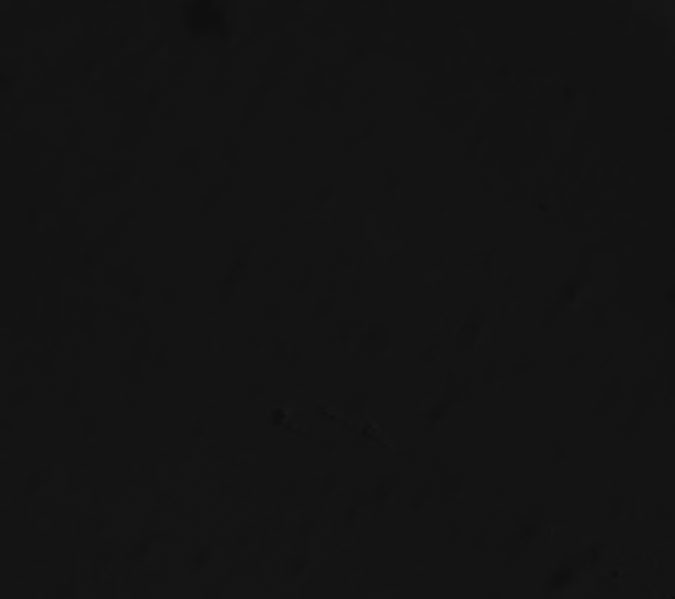

Supplement: Supplementary file 5 — Supplementary Code [file 41467_2023_36045_MOESM5_ESM.zip › Source Code/Untreated raw data for testing the code/PLGA NPs/Image240.jpg]

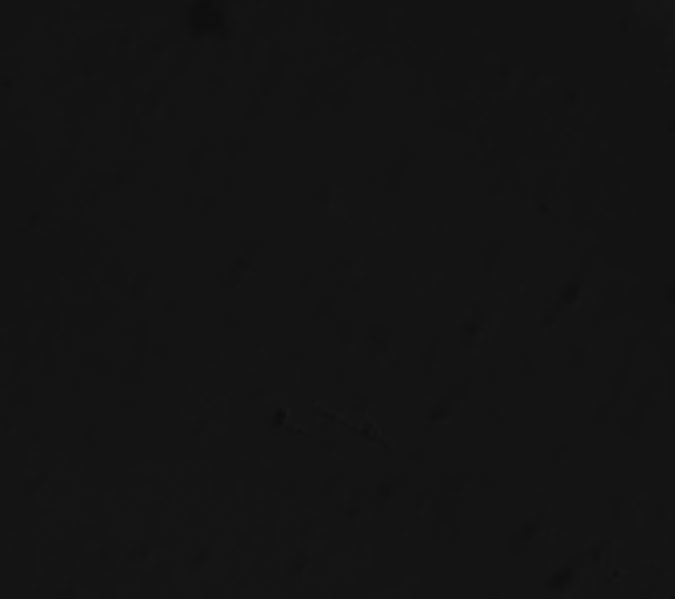

Supplement: Supplementary file 5 — Supplementary Code [file 41467_2023_36045_MOESM5_ESM.zip › Source Code/Untreated raw data for testing the code/PLGA NPs/Image254.jpg]

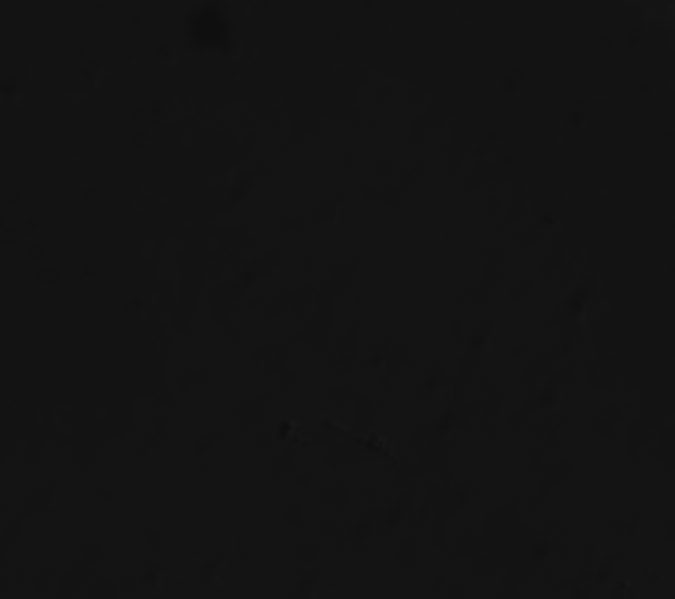

Supplement: Supplementary file 5 — Supplementary Code [file 41467_2023_36045_MOESM5_ESM.zip › Source Code/Untreated raw data for testing the code/PLGA NPs/Image49.jpg]

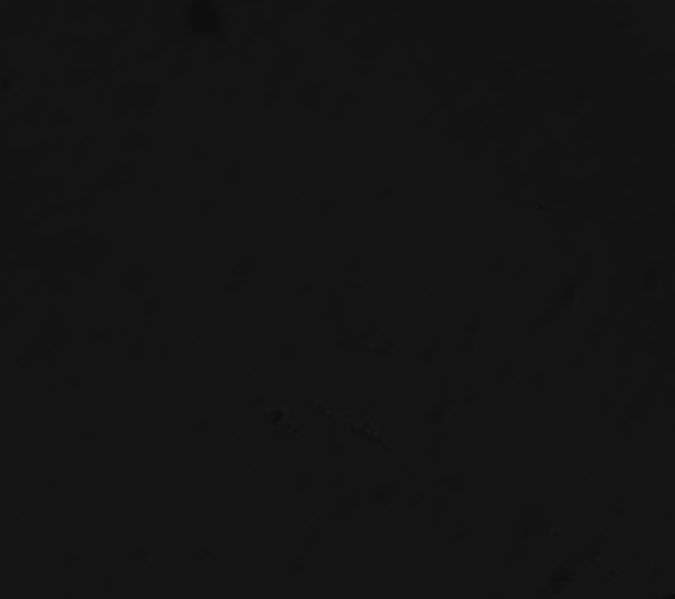

Supplement: Supplementary file 5 — Supplementary Code [file 41467_2023_36045_MOESM5_ESM.zip › Source Code/Untreated raw data for testing the code/PLGA NPs/Image532.jpg]

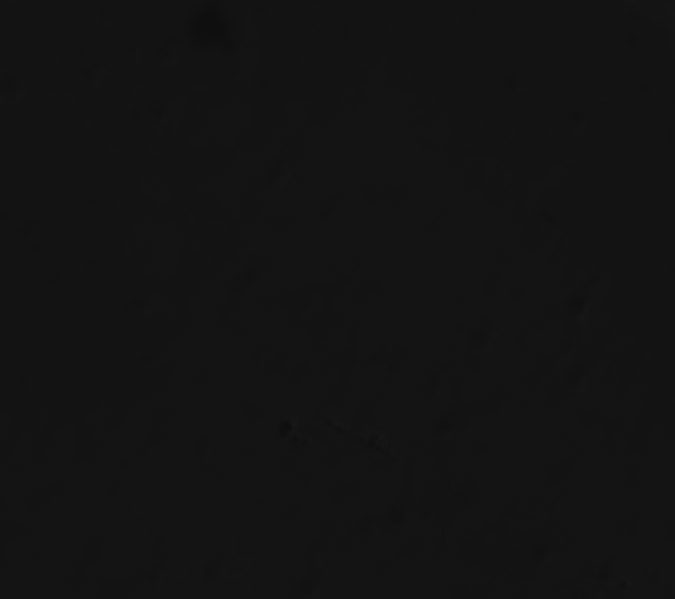

Supplement: Supplementary file 5 — Supplementary Code [file 41467_2023_36045_MOESM5_ESM.zip › Source Code/Untreated raw data for testing the code/PLGA NPs/Image61.jpg]

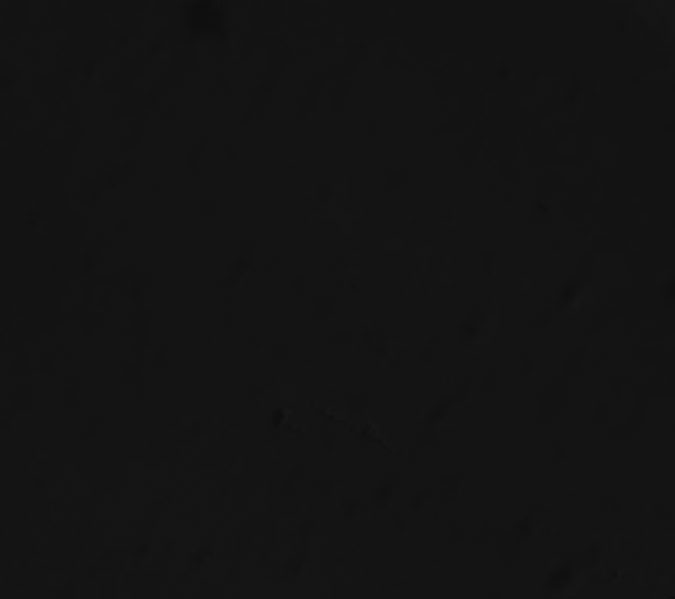

Supplement: Supplementary file 5 — Supplementary Code [file 41467_2023_36045_MOESM5_ESM.zip › Source Code/Untreated raw data for testing the code/PLGA NPs/Image268.jpg]

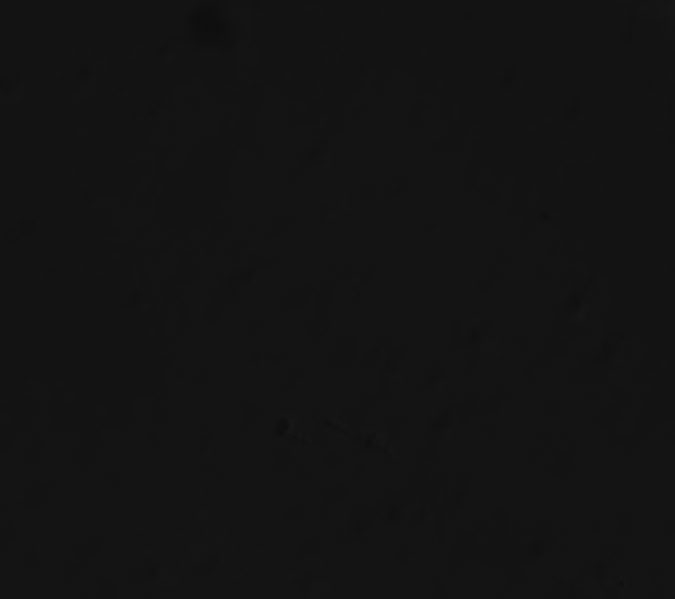

Supplement: Supplementary file 5 — Supplementary Code [file 41467_2023_36045_MOESM5_ESM.zip › Source Code/Untreated raw data for testing the code/PLGA NPs/Image75.jpg]

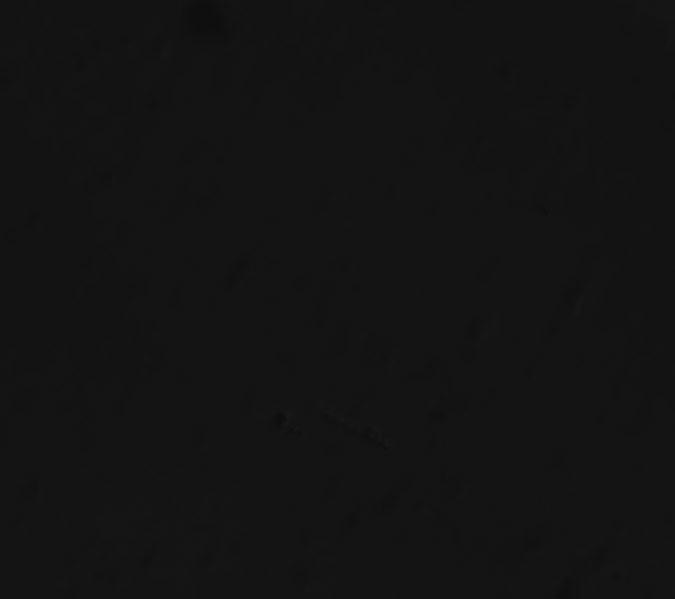

Supplement: Supplementary file 5 — Supplementary Code [file 41467_2023_36045_MOESM5_ESM.zip › Source Code/Untreated raw data for testing the code/PLGA NPs/Image136.jpg]

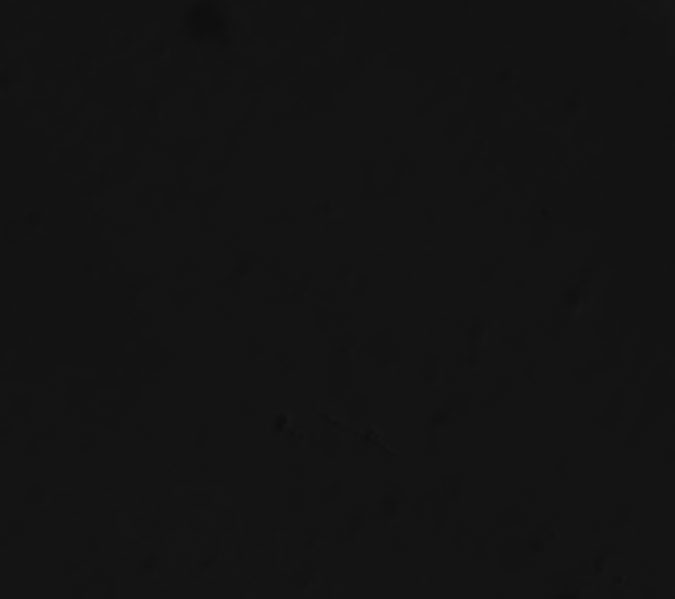

Supplement: Supplementary file 5 — Supplementary Code [file 41467_2023_36045_MOESM5_ESM.zip › Source Code/Untreated raw data for testing the code/PLGA NPs/Image122.jpg]

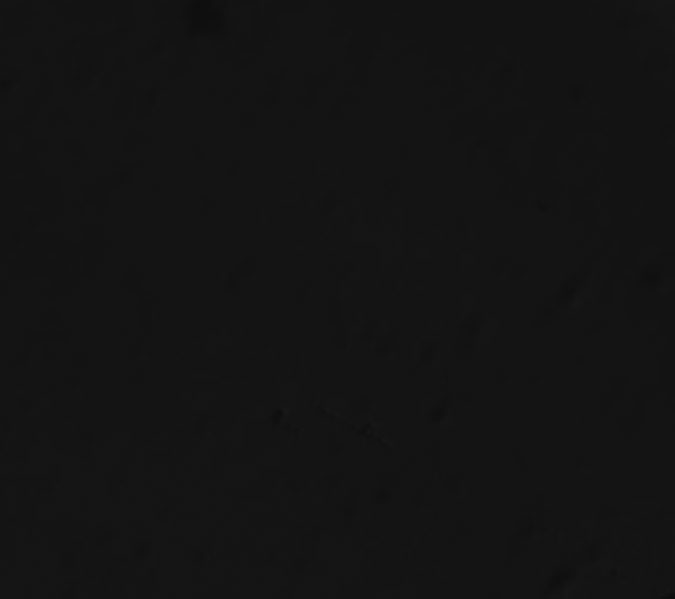

Supplement: Supplementary file 5 — Supplementary Code [file 41467_2023_36045_MOESM5_ESM.zip › Source Code/Untreated raw data for testing the code/PLGA NPs/Image491.jpg]

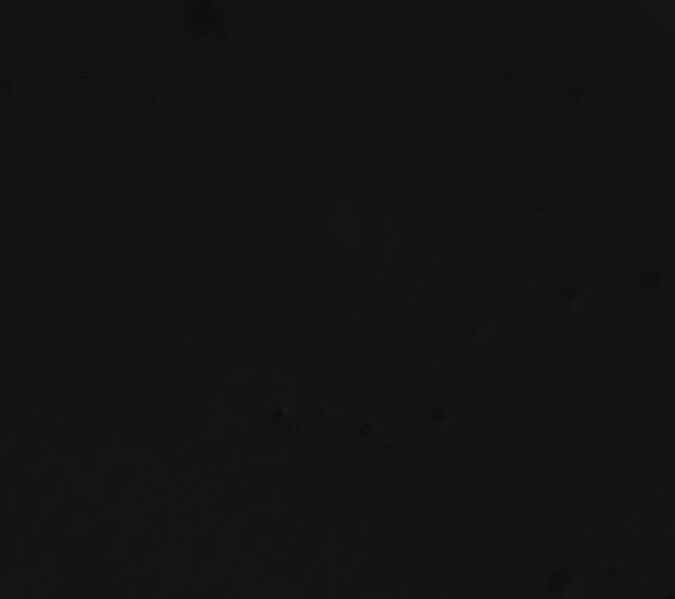

Supplement: Supplementary file 5 — Supplementary Code [file 41467_2023_36045_MOESM5_ESM.zip › Source Code/Untreated raw data for testing the code/PLGA NPs/Image485.jpg]

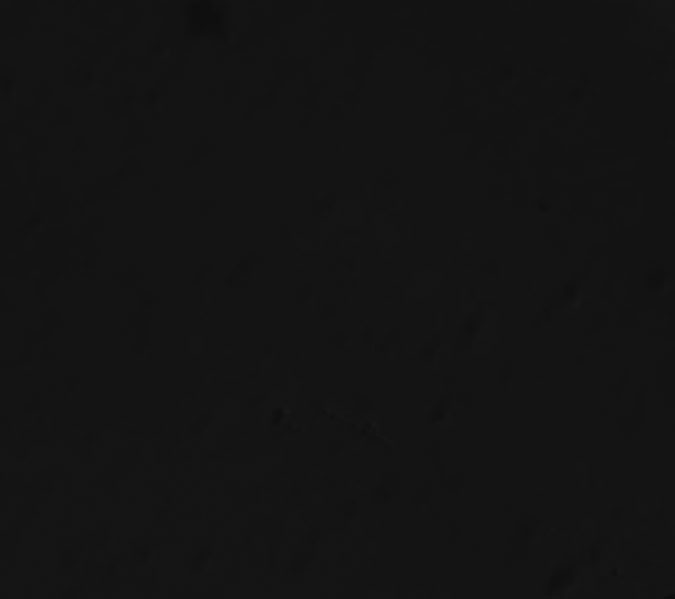

Supplement: Supplementary file 5 — Supplementary Code [file 41467_2023_36045_MOESM5_ESM.zip › Source Code/Untreated raw data for testing the code/PLGA NPs/Image452.jpg]

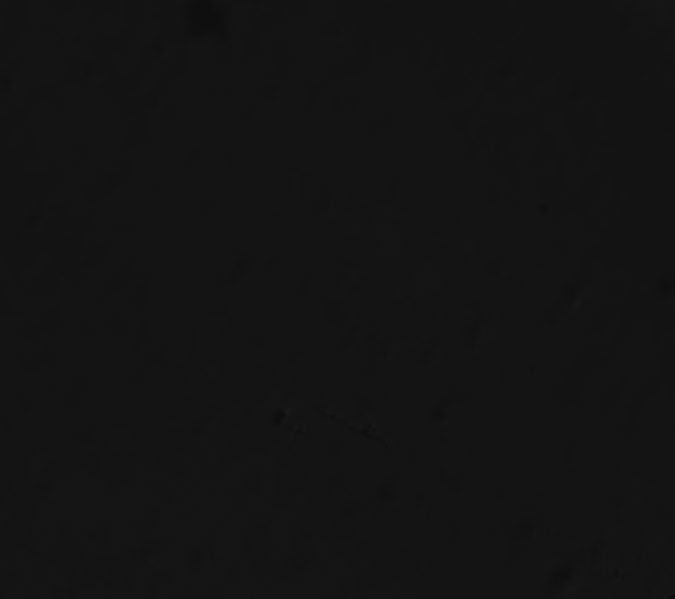

Supplement: Supplementary file 5 — Supplementary Code [file 41467_2023_36045_MOESM5_ESM.zip › Source Code/Untreated raw data for testing the code/PLGA NPs/Image334.jpg]

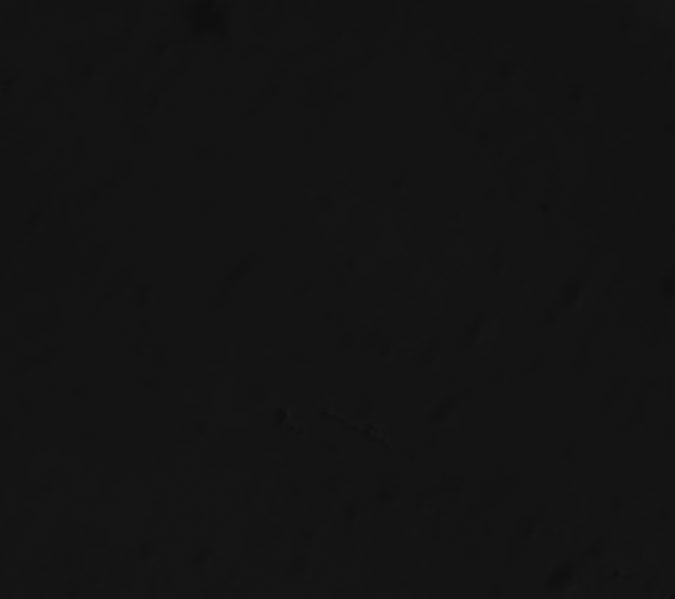

Supplement: Supplementary file 5 — Supplementary Code [file 41467_2023_36045_MOESM5_ESM.zip › Source Code/Untreated raw data for testing the code/PLGA NPs/Image320.jpg]

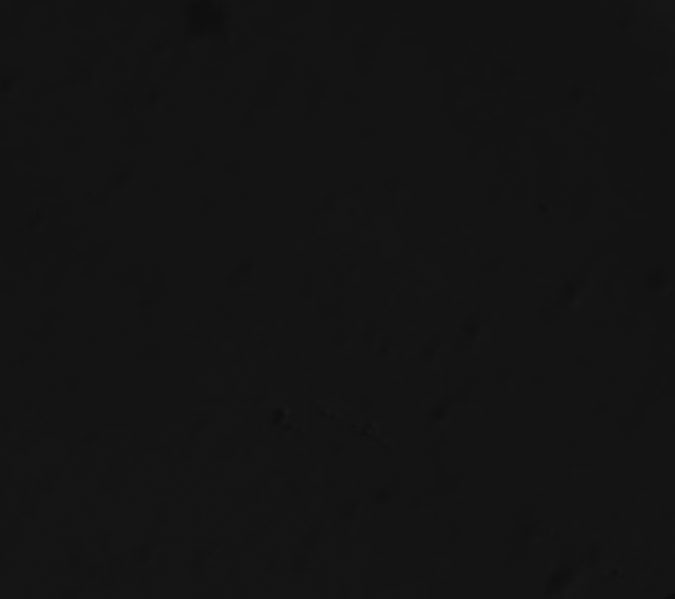

Supplement: Supplementary file 5 — Supplementary Code [file 41467_2023_36045_MOESM5_ESM.zip › Source Code/Untreated raw data for testing the code/PLGA NPs/Image446.jpg]

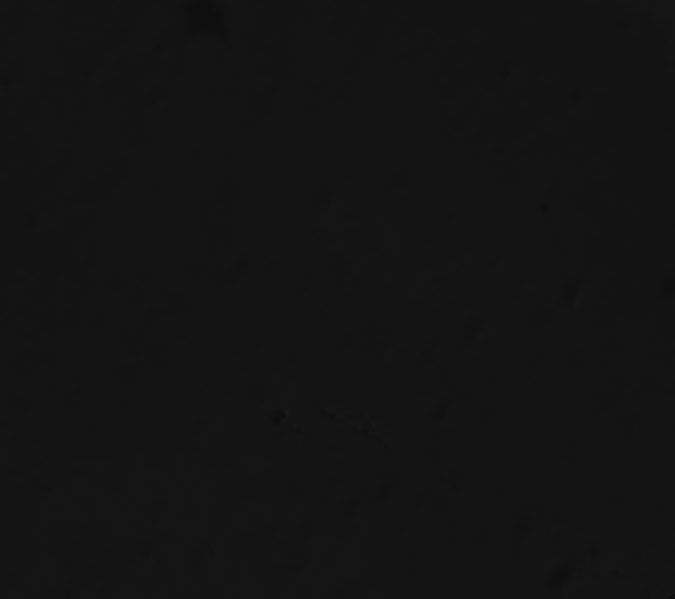

Supplement: Supplementary file 5 — Supplementary Code [file 41467_2023_36045_MOESM5_ESM.zip › Source Code/Untreated raw data for testing the code/PLGA NPs/Image308.jpg]

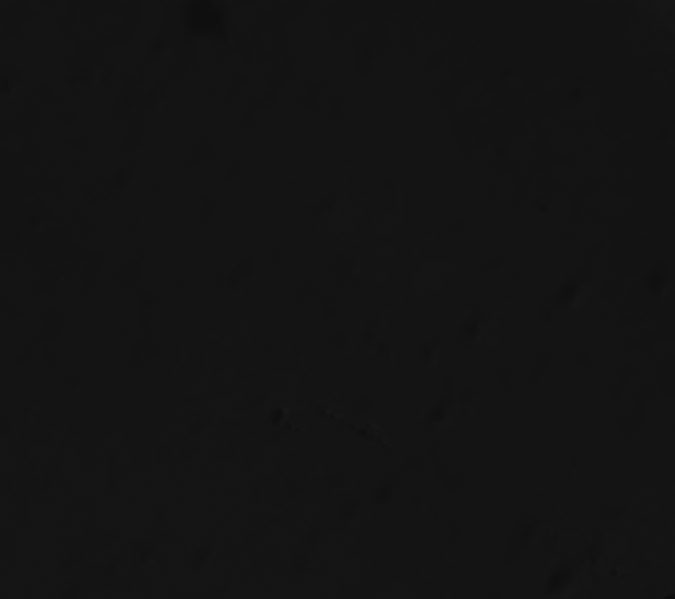

Supplement: Supplementary file 5 — Supplementary Code [file 41467_2023_36045_MOESM5_ESM.zip › Source Code/Untreated raw data for testing the code/PLGA NPs/Image442.jpg]

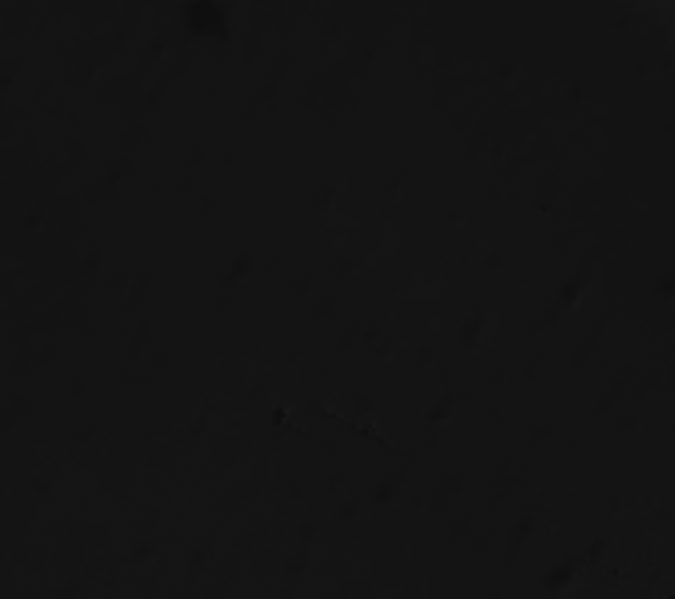

Supplement: Supplementary file 5 — Supplementary Code [file 41467_2023_36045_MOESM5_ESM.zip › Source Code/Untreated raw data for testing the code/PLGA NPs/Image324.jpg]

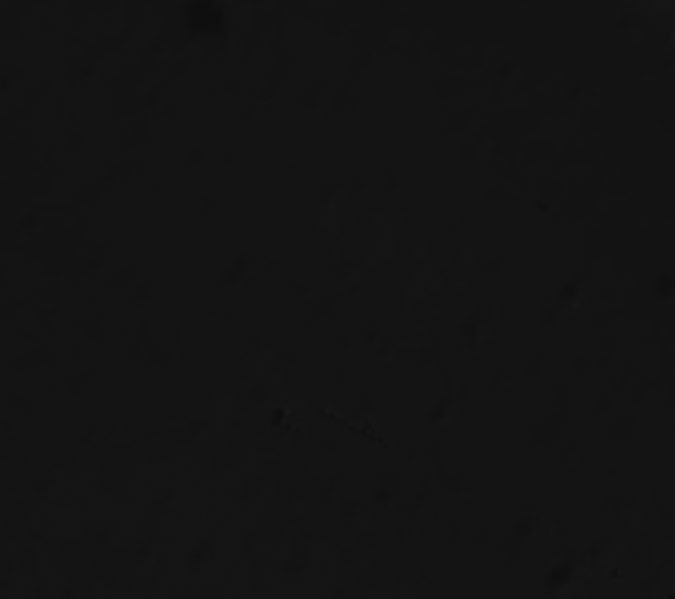

Supplement: Supplementary file 5 — Supplementary Code [file 41467_2023_36045_MOESM5_ESM.zip › Source Code/Untreated raw data for testing the code/PLGA NPs/Image330.jpg]

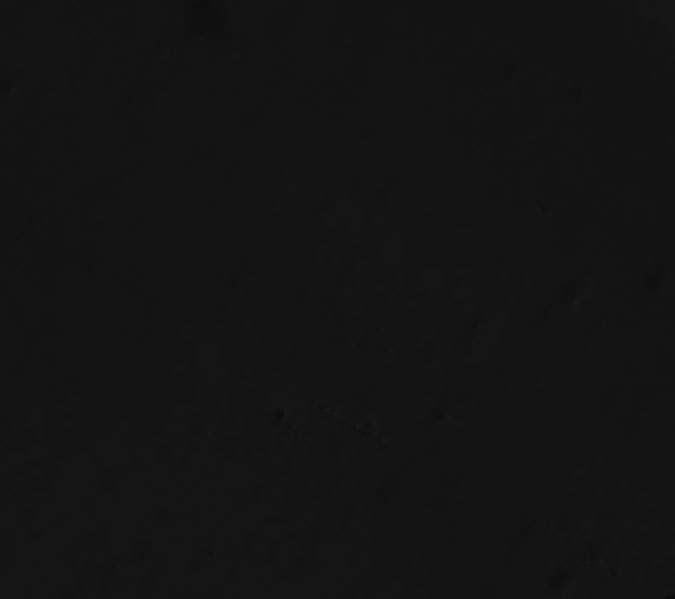

Supplement: Supplementary file 5 — Supplementary Code [file 41467_2023_36045_MOESM5_ESM.zip › Source Code/Untreated raw data for testing the code/PLGA NPs/Image456.jpg]

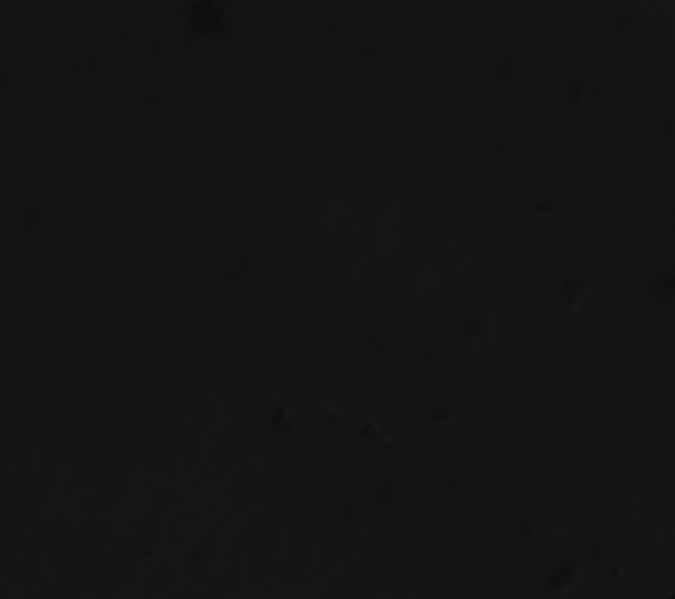

Supplement: Supplementary file 5 — Supplementary Code [file 41467_2023_36045_MOESM5_ESM.zip › Source Code/Untreated raw data for testing the code/PLGA NPs/Image318.jpg]

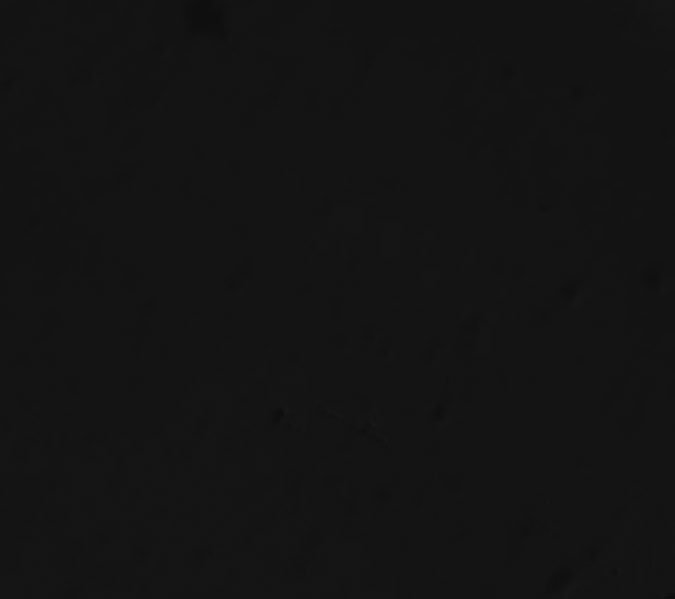

Supplement: Supplementary file 5 — Supplementary Code [file 41467_2023_36045_MOESM5_ESM.zip › Source Code/Untreated raw data for testing the code/PLGA NPs/Image481.jpg]

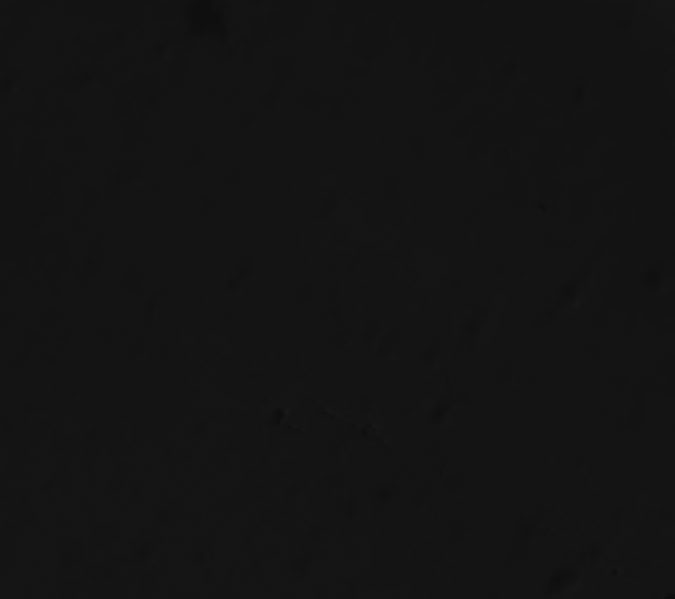

Supplement: Supplementary file 5 — Supplementary Code [file 41467_2023_36045_MOESM5_ESM.zip › Source Code/Untreated raw data for testing the code/PLGA NPs/Image495.jpg]

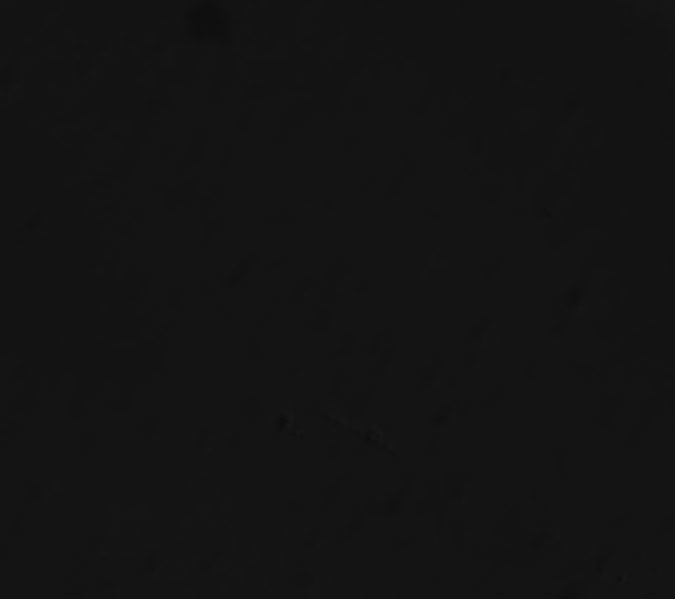

Supplement: Supplementary file 5 — Supplementary Code [file 41467_2023_36045_MOESM5_ESM.zip › Source Code/Untreated raw data for testing the code/PLGA NPs/Image126.jpg]

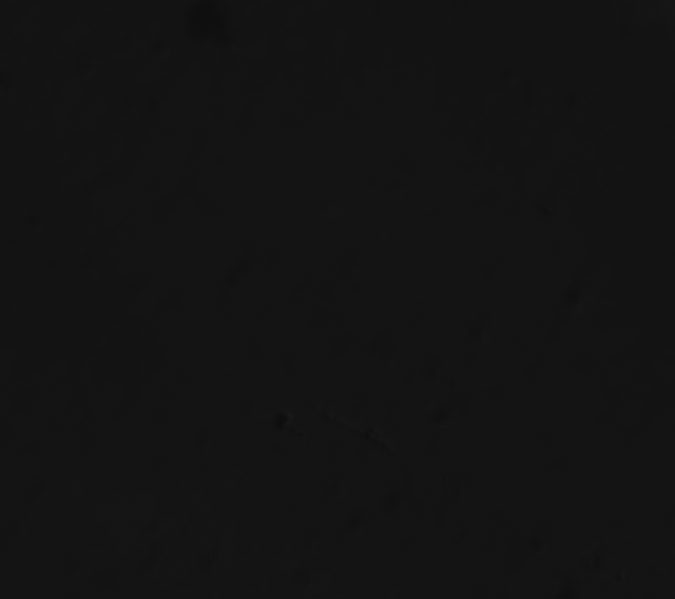

Supplement: Supplementary file 5 — Supplementary Code [file 41467_2023_36045_MOESM5_ESM.zip › Source Code/Untreated raw data for testing the code/PLGA NPs/Image132.jpg]

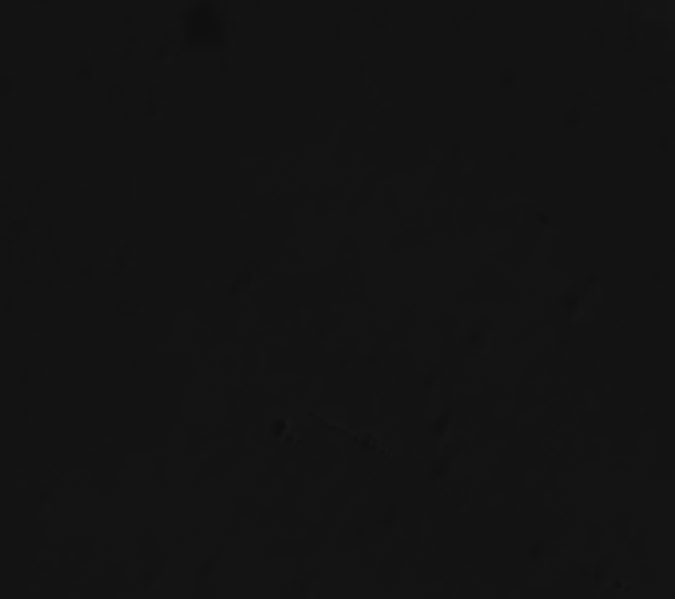

Supplement: Supplementary file 5 — Supplementary Code [file 41467_2023_36045_MOESM5_ESM.zip › Source Code/Untreated raw data for testing the code/PLGA NPs/Image8.jpg]

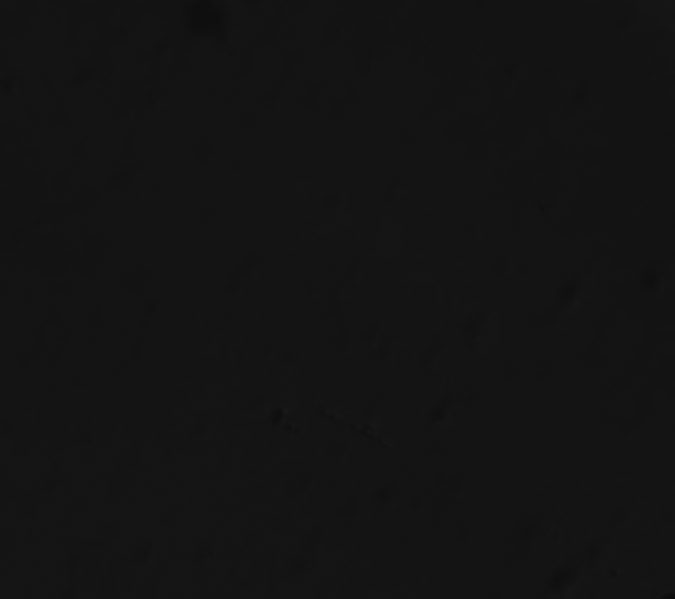

Supplement: Supplementary file 5 — Supplementary Code [file 41467_2023_36045_MOESM5_ESM.zip › Source Code/Untreated raw data for testing the code/PLGA NPs/Image536.jpg]

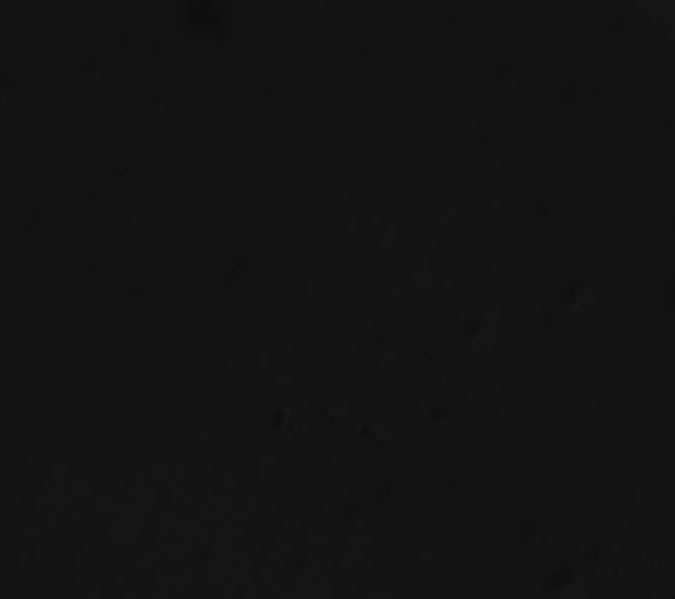

Supplement: Supplementary file 5 — Supplementary Code [file 41467_2023_36045_MOESM5_ESM.zip › Source Code/Untreated raw data for testing the code/PLGA NPs/Image250.jpg]

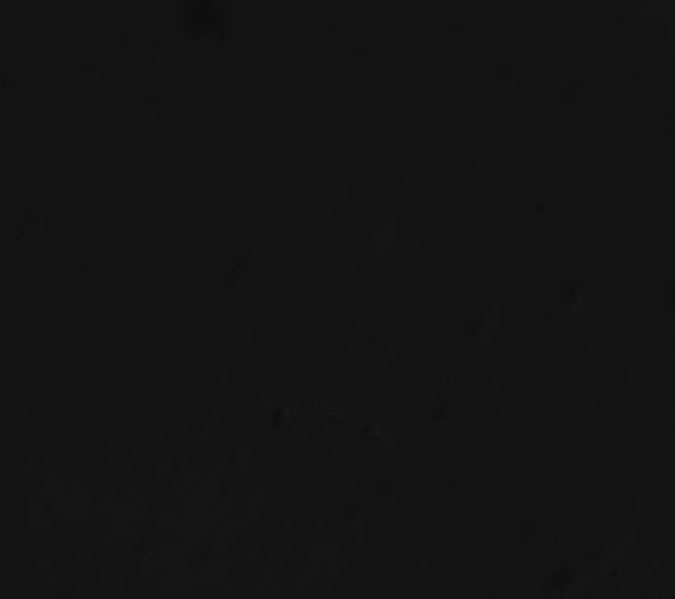

Supplement: Supplementary file 5 — Supplementary Code [file 41467_2023_36045_MOESM5_ESM.zip › Source Code/Untreated raw data for testing the code/PLGA NPs/Image244.jpg]

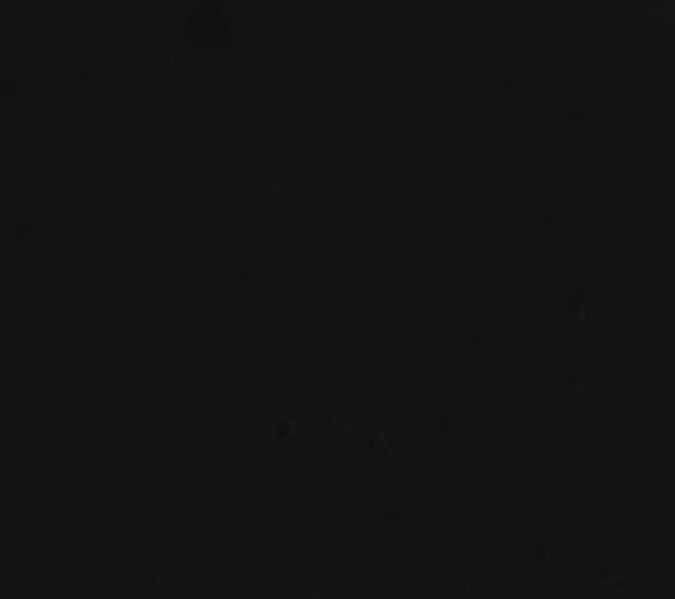

Supplement: Supplementary file 5 — Supplementary Code [file 41467_2023_36045_MOESM5_ESM.zip › Source Code/Untreated raw data for testing the code/PLGA NPs/Image59.jpg]

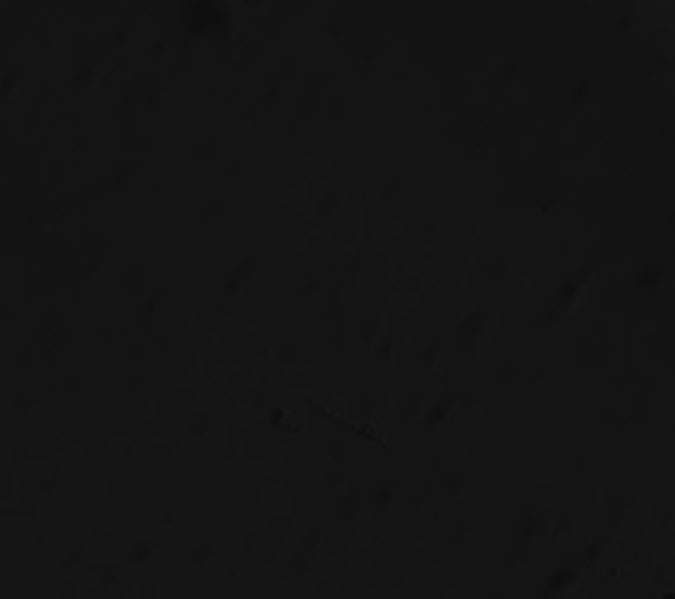

Supplement: Supplementary file 5 — Supplementary Code [file 41467_2023_36045_MOESM5_ESM.zip › Source Code/Untreated raw data for testing the code/PLGA NPs/Image522.jpg]

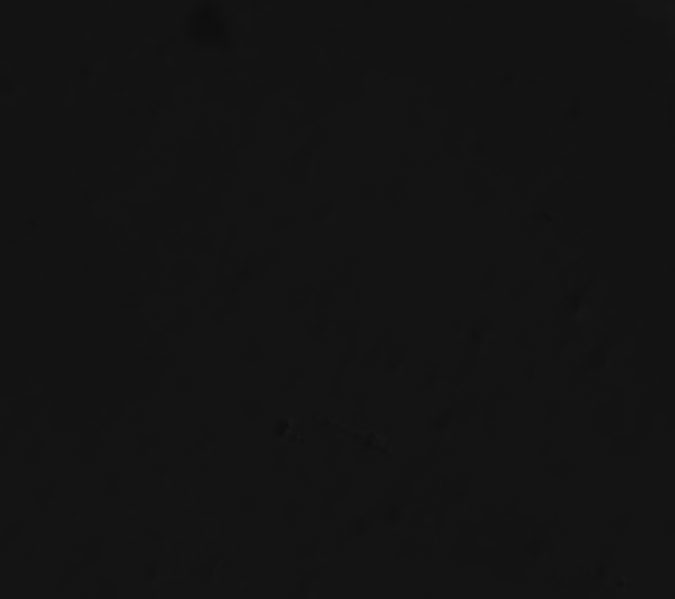

Supplement: Supplementary file 5 — Supplementary Code [file 41467_2023_36045_MOESM5_ESM.zip › Source Code/Untreated raw data for testing the code/PLGA NPs/Image71.jpg]

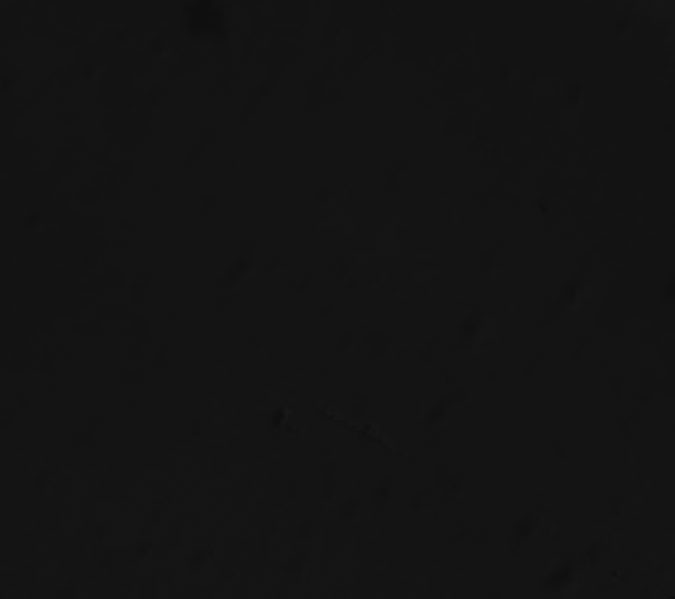

Supplement: Supplementary file 5 — Supplementary Code [file 41467_2023_36045_MOESM5_ESM.zip › Source Code/Untreated raw data for testing the code/PLGA NPs/Image278.jpg]

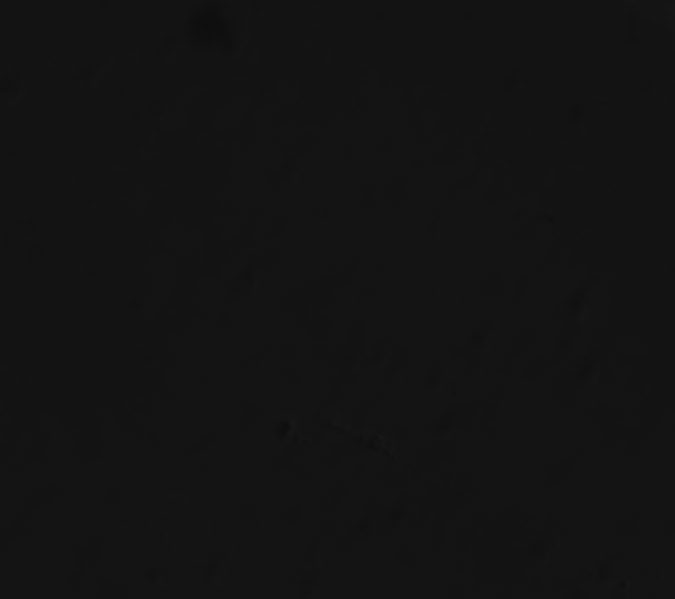

Supplement: Supplementary file 5 — Supplementary Code [file 41467_2023_36045_MOESM5_ESM.zip › Source Code/Untreated raw data for testing the code/PLGA NPs/Image65.jpg]

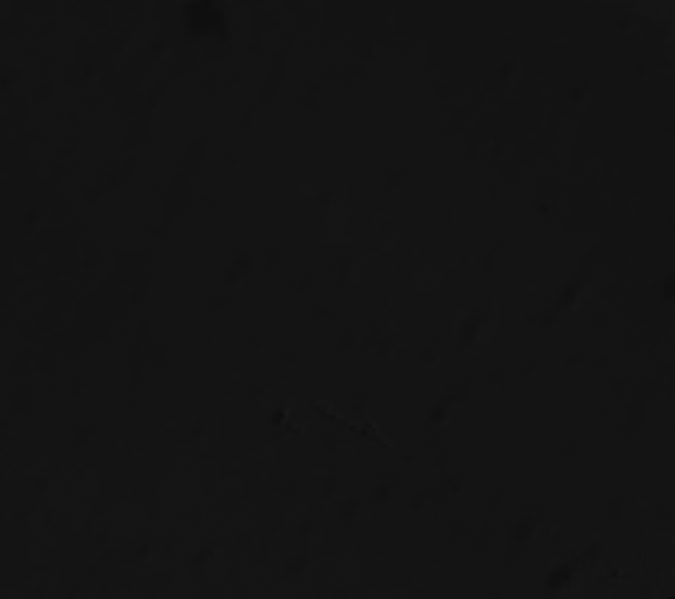

Supplement: Supplementary file 5 — Supplementary Code [file 41467_2023_36045_MOESM5_ESM.zip › Source Code/Untreated raw data for testing the code/PLGA NPs/Image293.jpg]

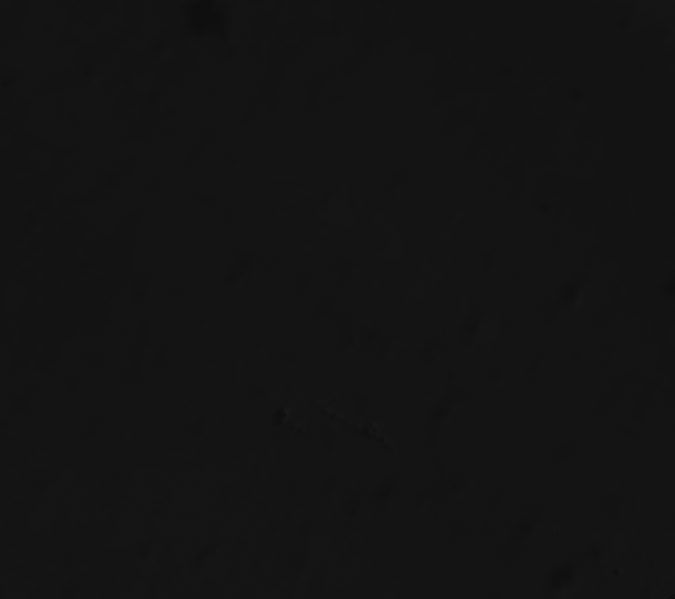

Supplement: Supplementary file 5 — Supplementary Code [file 41467_2023_36045_MOESM5_ESM.zip › Source Code/Untreated raw data for testing the code/PLGA NPs/Image287.jpg]

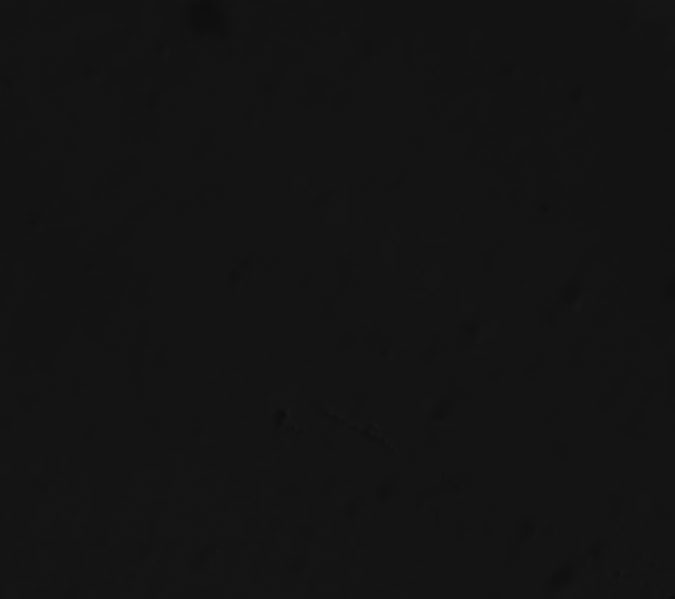

Supplement: Supplementary file 5 — Supplementary Code [file 41467_2023_36045_MOESM5_ESM.zip › Source Code/Untreated raw data for testing the code/PLGA NPs/Image286.jpg]

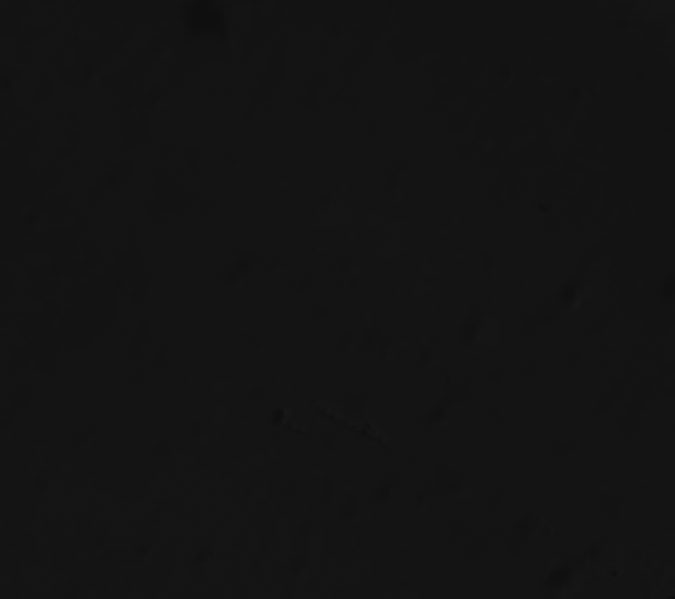

Supplement: Supplementary file 5 — Supplementary Code [file 41467_2023_36045_MOESM5_ESM.zip › Source Code/Untreated raw data for testing the code/PLGA NPs/Image292.jpg]
